# Hydrolytically Stable and Thermo-Mechanically Tunable Poly(Urethane) Thermoset Networks that Selectively Degrade and Generate Reusable Molecules

**DOI:** 10.1021/acsami.2c00485

**Published:** 2022-05-03

**Authors:** Keith B. Sutyak, Erick B. Iezzi, Grant C. Daniels, Eugene Camerino

**Affiliations:** † U.S. Naval Research Laboratory, Chemistry Division, Washington, DC 20375, United States; ‡ ASEE Post-Doctoral Fellow, U.S. Naval Research Laboratory, Chemistry Division, Washington, DC 20375, United States

**Keywords:** degradable network, polyurethane thermoset, cross-linked, cascading bond cleavages, reusable, fluoride ion

## Abstract

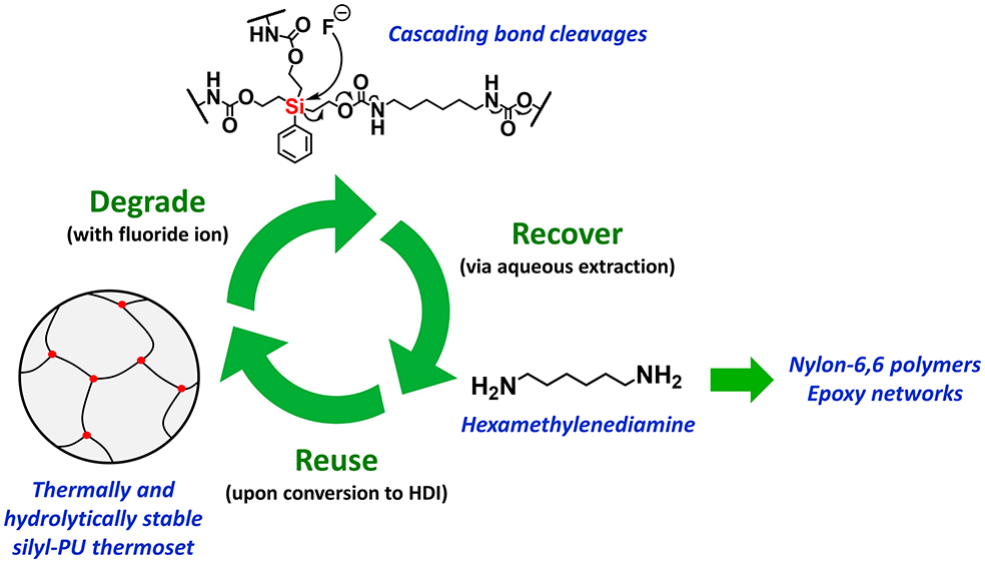

Cross-linked polymeric networks that possess tunable properties and degrade on-demand have broad applications in today’s society. Herein, we report on silyl-containing poly(urethane) (silyl-PU) thermoset networks, which are highly cross-linked stimuli-responsive materials with hydrolytic stability at 37.7 °C and 95% relative humidity, thermal stability of 280–311.2 °C, tensile properties of 0.38–51.7 MPa strength and 73.7–256.4% elongation, including storage modulus of 2268–3499 MPa (in the glassy state). However, unlike traditional (i.e., nondegradable) PU thermosets, these silyl-PUs selectively activate with fluoride ion under mild and static conditions to completely degrade, via cascading bond cleavages, and generate recoverable and reusable molecules. Silyl-PUs, as thin films, also demonstrated complete removal (within 30 min) from a strongly adhered epoxy thermoset network without altering the structure of the latter. Silyl-PU thermosets have potential applications in composite parts, vehicle and industrial coatings, and rigid plastics for personal devices, and may reduce environmental waste compared to nondegradable, single-use materials.

## 1 Introduction

Thermosetting polymer networks, commonly referred to as thermosets, are chemically cross-linked and irreversibly hardened networks that are formed from the reaction of organic molecules with functional groups. Inclusion of these cross-links produces networks with enhanced properties and durability, such as increased thermal and dimensional stability, high chemical resistivity, and improved resistance to oxidative degradation from sunlight.^1,2^ Thermosetting networks are vital to modern society and are ubiquitous in industrial and consumer materials, such as foams for refrigeration equipment,^3^ coatings for vehicles,^4^ construction adhesives,^5^ printed circuit boards, and components for personal computers,^6,7^ including lightweight composites for aircraft.^8^ However, the covalent cross-links render these networks extremely difficult to degrade without employing hazardous chemical treatments,^9,10^ mechanical processing,^11^ or pyrolysis,^12^ and they cannot be repaired, reprocessed, or solvated.^13^ This presents environmental issues due to the lack of recyclable thermosets or those that generate reusable molecules upon degradation.^14−16^ Global thermoset production is expected to surpass 65 million tons during the current decade,^17^ and the vast majority of these single-use materials will be landfilled once their end-of-life service is reached.^18^

Poly(urethane)s (PUs) represent one of the most common and versatile thermosetting materials due to the numerous polyol and isocyanate components that can be employed to tailor properties,^19,20^ yet PUs suffer from a lack of reprocessing, recycling, and degradability due to their thermodynamically stable carbamate (urethane) linkages.^21^ To address these issues, researchers have developed dynamic cross-linked PU networks that employ reversibly labile and covalent bond exchance reactions. Examples include dissociative networks based on hindered urea linkages,^22^ as well as associative networks, known as vitrimers, that enable reprocessing via catalyzed carbamate exchange or transcarbonation reations.^21,23,24^ However, these dynamic networks require high temperatures to facilitate the exchange reactions, resulting in loss of integrity for dissociative networks, and they commonly exhibit creep due to their dynamic nature, which affects long-term dimensional stability.^25−27^ Degradable PU networks have been designed by incorporating labile bonds that degrade under basic conditions,^28,29^ at elevated temperatures,^30^ or with enzymes.^31^ However, many of these linkages are thermally and/or hydrolytically unstable, which prevents their commercial use in lieu of traditional PU thermosets.

The incorporation of silyl ether (Si–O–C) cross-links in thermosetting networks has created unique hybrid materials that degrade when exposed to certain stimuli and under specific conditions. For instance, biomaterials with silyl ether cross-links demonstrated controlled degradation under the acidic conditions found in tumor tissue and diseased cells,^32^ and a small percentage of silyl ether cross-links in poly(dicyclopentadiene) composite networks were degraded with fluoride ions to recover the embedded carbon fiber.^33^ Additionally, silyl ether protected phenolic cross-linkers in adhesives were degraded under similar fluoride-rich conditions.^34,35^ However, silyl ether linkages, especially those without bulky substituents, are hydrolytically unstable and will inevitably cleave under ambient atmospheric conditions.^36−38^ Therefore, networks designed with these cross-links may experience premature degradation and a loss of integrity before the external stimulus is applied, thus rendering them impractical for commercial applications where durability is paramount.

During the past few years our research group has focused on designing stimuli-responsive molecules that possess silyl (Si–C) bonds. These bonds, often as silyl-centered ethoxycarbonyls, were designed to be hydrolytically stable, yet selectively react with fluoride ions to initiate disassembly of attached aliphatic chains via cascading bond cleavages.^39,40^ Herein, we have leveraged this knowledge to develop highly cross-linked and stimuli-responsive silyl-containing poly(urethane) (silyl-PU) thermosets that resemble the network structure of traditional, nondegrdable PU thermosets.^19,41−43^ These silyl-PUs demonstrate excellent hydrolytic and thermal stability, including tunable thermo-mechanical properties, whereas their design enabled selective degradation with a fluoride salt at room temperature to generate reusable molecules. Furthermore, we demonstrate that these silyl-PUs, when strongly adhered to an epoxy thermoset, can be selectively removed without affecting the chemical structure of the underlying network.

## 2 Results and Discussion

### 2.1 Network Synthesis and Properties

**Figure 1:**
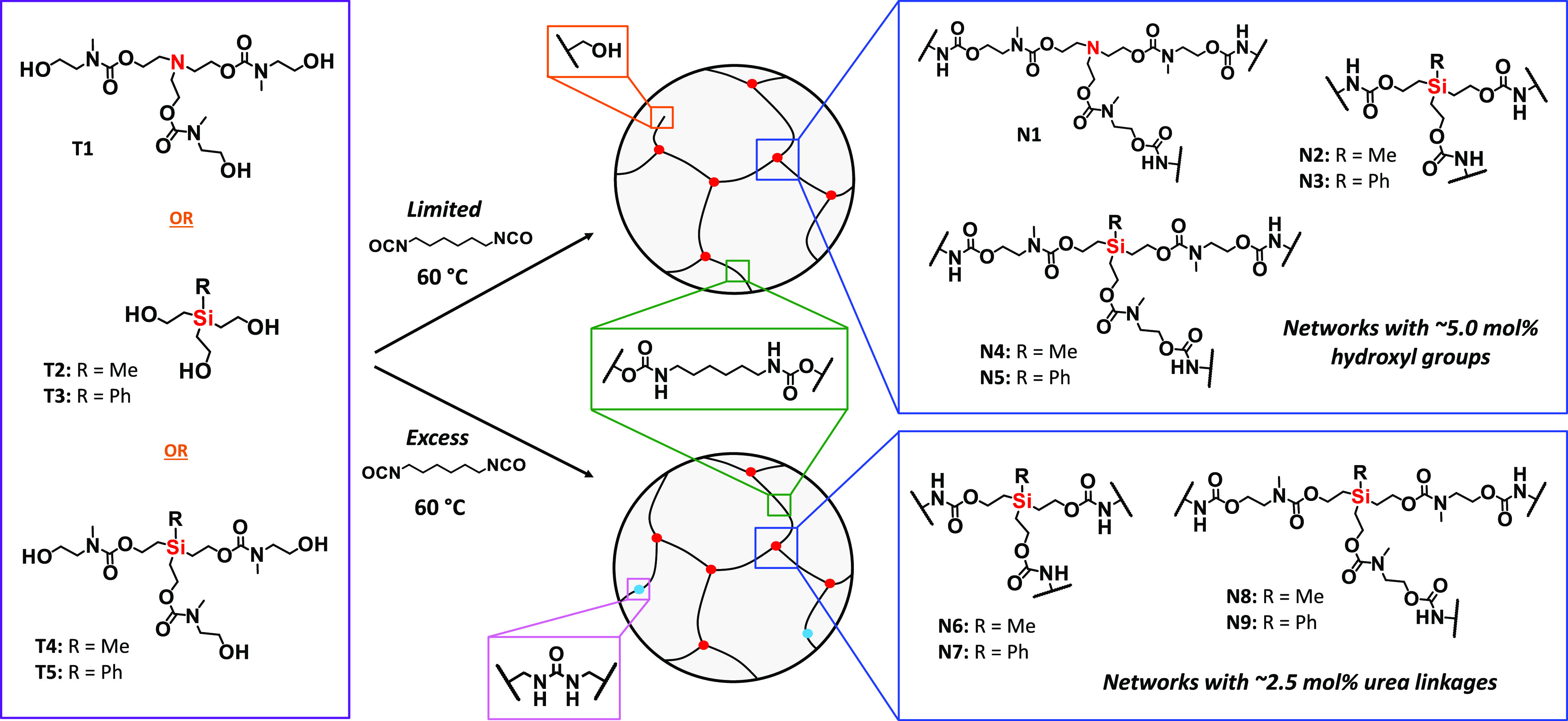
Chemical structure of silyl-centered triols (**T2**–**T5**) and extended chain triethanolamine control (**T1**) cross-linkers, including the synthesis of poly(urethane) thermoset networks **N1**–**N9** via reaction with limited or excess HDI at 60 °C. Networks **N1**–**N5** possessed a slight excess of free hydroxyl groups (orange expansion box), whereas networks **N6**–**N9** possessed a small percentage of urea linkages (pink expansion box). The central atom of the cross-linkers are highlighted in red, the blue expansion boxes show the structure of each cross-linked triol and newly formed carbamate linkages, and the green expansion box shows the aliphatic hexamethylene chains in each network.

Small molecule silyl-centered triols (**T2**–**T3**) and extended chain silyl-centered triols (**T4**–**T5**) were synthesized for use as cross-linkers in silyl-PU networks (Figure 1 and Supporting Information (SI) Scheme S1B; see Experimental Section and Supporting Information for details). These molecules were synthesized based on previous research by our group where we showed that increasing the number of cleavable Si–C bonds and the length of covalently bound appendages in a molecule resulted in an increased rate of disassembly.^40^ Herein, the silyl-centered cross-linkers should theoretically disassemble in three directions. The added dimension of disassembly is hypothesized to provide an increased rate of network degradation when the silyl-PUs are activated with a chemical stimulus. The silyl-centered triols were synthesized with a methyl or phenyl functional group covalently bonded to silicon to determine the impact of electronic and steric factors on network properties and time of disassembly. Additionally, extended chain methyl silyl-centered triol (**T4**) and extended chain phenyl silyl-centered triol (**T5**) were synthesized to determine how cross-linker chain length influenced network properties and time of degradation. Extended chain triethanolamine (**T1**) (Figure 1 and SI Scheme S1A; see Experimental Section for details), which does not contain silyl linkages, was synthesized for use as a control cross-linker for the following reasons: (1) it resembles the structure and size of triols used in numerous PU networks,^19^ (2) it lacks a central fluoride-responsive silicon trigger, and (3) it contains similar bonds and linkages as the synthesized silyl-centered triols.

**Figure 2:**
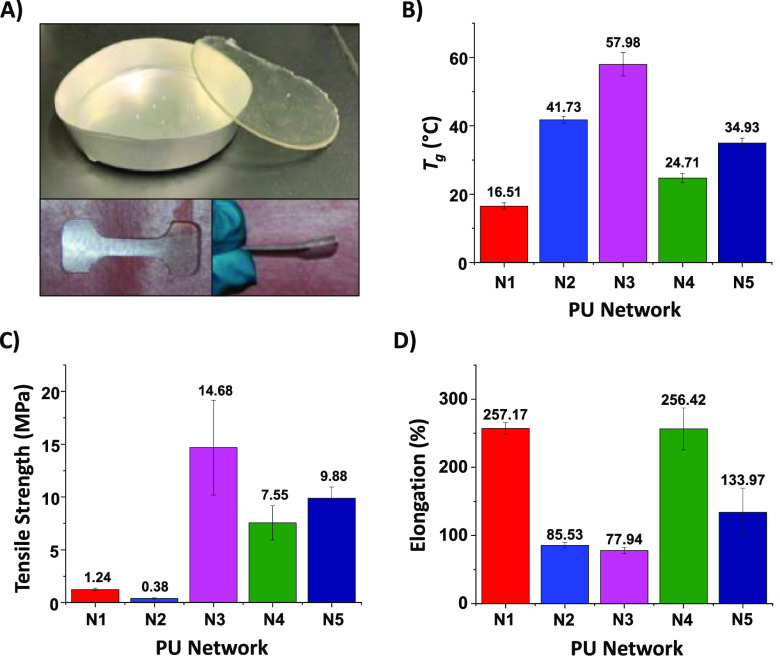
PU thermoset networks and properties. (A) Photographs of a silyl-PU network: (top) when cast in an aluminum pan and upon removal, (bottom, left) a dog bone shaped sample of 3.6 cm length and 1.4 cm width, and (bottom, right) side of dog bone shaped sample showing thickenss of about 1 mm. (B) Glass transtion temperature (*T*_g_) of networks **N1**–**N5**. (C) Ultimate tensile strength of networks **N1**–**N5**. (D) Percent elongation at break for network **N1**–**N5**.

Silyl-PU networks **N2**–**N5** were synthesized by mixing a silyl-centered triol (**T2**–**T5**) with 1,6-hexamethylene diisocyanate (HDI), which is a linear aliphatic isocyanate used in traditional PUs, heating the mixture at 60 °C (Figure 1), followed by casting into aluminum pans to form 1–2 mm thick pucks (Figure 2A; see Experimental Section and Supporting Information for details). A nonsilyl-containing PU control (**N1**) was synthesized by reacting extended chain triethanolamine (**T1**) with HDI using the same procedure (see Experimental Section for details). Networks **N1**–**N5** were synthesized using a 5% molar excess of hydroxyl (−OH) functionality, whereas networks **N6**–**N9** were synthesized using a silyl triol and a 5% molar excess of isocyanate (−NCO) functionality (see Supporting Information for details). Samples with excess isocyanate were fabricated to determine differences in silyl-PU thermal stability, mechanical properties, and time of degradation due to OH:NCO indexing, as excess isocyanate functionality can react with atmospheric moisture to form amines and subsequent urea linkages.^44^ Attempts to generate a control network with **T1** and excess isocyanate resulted in foamed samples due to tertiary amine catalysis of the excess isocyanate groups in the presence of moisture.^45^

Spectroscopic analysis provided confirmation of network formation for all PU thermosets. Attenuated total reflectance infrared (ATR-IR) spectroscopy of **N1**–**N5** and **N6**–**N9** confirmed the formation of carbamate linkages as shown by the carbonyl stretch around 1680 cm^–1^, a N–H stretch around 3300 cm^–1^, and an in-plane carbamate bend around 1520 cm^–1^ (SI Figures S11 and S12). For **N1**–**N5**, the isocyanate peak at 2270 cm^–1^ was not detected due to complete consumption as a result of the excess hydroxyl functionality. Isocyanate was also not detected in **N6**–**N9**, indicating the excess functionality had reacted with atmospheric moisture to form amines and subsequent urea linkages. For all networks, the Si–C bond stretch was located around 780 cm^–1^. Gel fraction calculations of all PU networks were 0.95 or greater, indicating the formation of highly cross-linked networks (Table 1 and SI Table S1).

**Table 1:** Label and Description, Gel Fraction, And Onset Degradation Temperature of PU Thermoset Networks **N1**–**N5**

thermoset network	central group	OH:NCO ratio	chain extension	gel fraction	onset deg. temp. (°C)
N1	amine	1.05:1.00	Y	0.95	244.6
N2	methyl-Si	1.05:1.00	N	0.97	311.2
N3	phenyl-Si	1.05:1.00	N	0.97	309.7
N4	methyl-Si	1.05:1.00	Y	0.99	284.3
N5	phenyl-Si	1.05:1.00	Y	0.97	291.4

Network thermal properties were obtained via thermogravimetric analysis (TGA) and differentical scanning calorimetry (DSC). TGA analysis showed that onset degradation temperatures for silyl-PUs **N2**–**N9** ranged from 280 to 311.2 °C (Table 1 and SI Table S1), which is excellent thermal stability and typical of highly cross-linked poly(urethane) networks,^46^ whereas the nonsilyl PU control (**N1**) had an onset degradation temperature of 244.6 °C (Table 1). The lower onset degradation temperature for **N1** is likely due to tertiary amine catalyzed hydrolysis of carbamate linkages within the cross-links upon heating. All silyl-PU networks (**N2**–**N9**) exhibited a similar degradation profile as the control (**N1**) (SI Figure S13). DSC provided the glass transition temperature (*T*_g_) of each network, which revealed the impact that structural variance (e.g., aliphatic chain length, presence of *N*-methyl carbamate linkages) had on the thermal properties of the silyl-PUs (Figure 2B). The chain-length of the silyl-centered triol had the greatest influence on the thermal *T*_g_ of the networks. Networks **N4** (*T*_g_ = 24.7 °C) and **N5** (*T*_g_ = 34.9 °C), which were based on extended chain silyl triols, had a significantly lower *T*_g_ than networks **N2** (*T*_g_ = 41.7 °C) and **N3** (*T*_g_ = 57.9 °C), which were based on nonextended silyl triols. We suspect the extended aliphatic segments enabled greater molecular motions (e.g., bond rotations) within the cross-links, which resulted in the lower *T*_g_ for networks **N4** and **N5**. Differences in *T*_g_ were also observed depending on the fourth substituent bound to the central silicon atom of each cross-link. In general, networks that possessed a phenyl group had a greater *T*_g_ than those with a methyl group. This difference is likely attributed to physical cross-links resulting from π-bonding interactions between the phenyl groups,^19^ whereas methyl groups are void of these interactions. The *T*_g_ of the nonsilyl PU control (**N1**) (*T*_g_ = 16.5 °C), which also possessed extended chains, was lower than **N4** and **N5**. However, the control lacked a fourth substituent at the center of each cross-link, which likely enabled greater molecular motion by the tertiary amine. For silyl-PUs **N6**–**N9**, methyl containing **N6** had a similar *T*_g_ to **N2**, while **N7**–**N9** demonstrated slightly greater *T*_g_ values than **N3**–**N5** (SI Figure S14). As expected, the urea linkages within **N6**–**N9**, although minute, provided increased hydrogen bonding interactions that resulted in the slightly greater glass transition temperatures.

Tensile testing of **N1**–**N9** was performed with a texture analyzer at room temperature (21 °C) to determine network strength and elongation (Figure 2C,D and SI Figures S15 and S16). The phenyl-containing silyl-PUs were the strongest of all the networks, as **N3** and **N5** demonstrated tensile values (at break) of 14.7 and 9.88 MPa, respectively, whereas **N7** and **N9** demonstrated values of 51.7 and 16.1 MPa, respectively. For these networks, we suspect that the phenyl groups provided increased toughness due to π-bonding interactions between cross-linked chains, which is common for traditional PUs with aromatic segments,^19^ whereas **N7** and **N9** exhibited additional strength due to hydrogen bonding of urea linkages between chains. Networks **N5** and **N9** possessed reduced toughness that is likely attributed to increased moleclar motions within the extended aliphatic chains. The tensile strength for the nonsilyl PU control (**N1**) was only 1.24 MPa, which is low, though not an anomaly when compared to several reported PUs.^47−49^ Conversely, networks with extended chains demonstrated greater elongation than networks without extended chains. Silyl-PUs **N2** and **N4** had values of 85.5% and 256.4%, respectively, whereas **N3** and **N5** were 77.9% and 133.9%, respectively. Similar trends were observed for **N6**–**N9**, although **N8** possessed a lower elongation than **N9**. Silyl-PU network **N4** had a similar elongation as the PU control (**N1**), yet possessed greater tensile strength, demonstrating that a silyl-PU can provide enhanced mechanical properties compared to a nonsilyl PU with similar structure. SI Table S2 provides a comparison of thermal and mechanical properties for several silyl-PUs (i.e., **N4**, **N5**, and **N7**), the nonsilyl PU control (**N1**), and several reported traditional PU networks.

**Figure 3:**
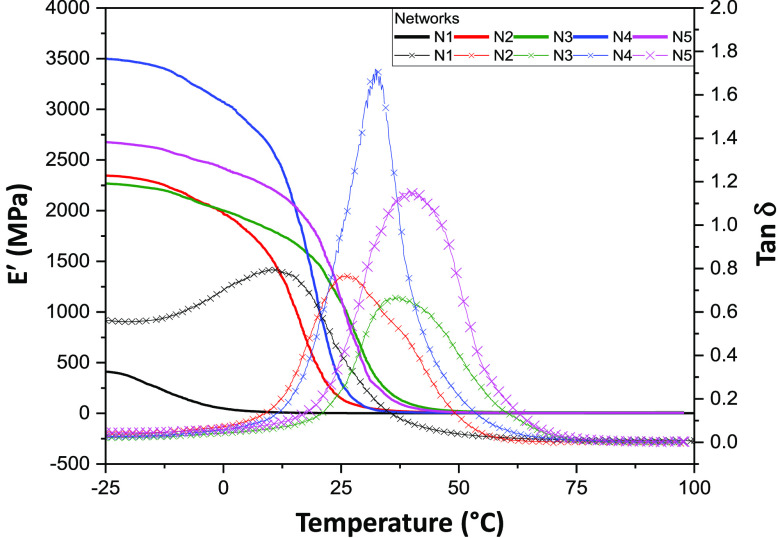
Temperature dependent storage modulus (E′) (solid line) and Tan Delta (Tan δ) (x-marked line) for thermoset networks **N1**–**N5**.

Dynamic mechanical analysis (DMA) was used to determine the storage (elastic) modulus (E′) for networks **N1**–**N5** over the temperature range −25 to 100 °C (Figure 3), and the change in network storage modulus (Δ*E*′) is provided in Table 2. The E′ for the networks in the glassy state ranged from 410.8 to 3499 MPa, whereas stiffness decreased in the rubbery state (above network *T*_g_) and was relatively constant above 75 °C, thus indicating stable cross-linked networks. Silyl-PU networks **N2**–**N5** all demostrated greater E′ values than the nonsilyl PU control (**N1**). Networks **N4** and **N5** had the greatest Δ*E*′ of the five networks, which is indicative of networks with lower cross-link density and supported by the longer aliphatic chains in these thermosets. Conversely, **N2** and **N3**, which are based on cross-linkers with shorter aliphatic chains, possessed a lower Δ*E*′ and greater cross-link density compared to **N4** and **N5**. Compared to **N2** and **N4**, we suspect the slightly greater Δ*E*′ values for networks **N3** and **N5** are attributed to intermolecular bonding between the pendant phenyl groups. The control (**N1**) possessed aliphatic chains smilar to **N4** and **N5**, yet had the lowest Δ*E*′, which is consistent with tensile results for this elastic material at room tempeature (above its *T*_g_ of 13.5 °C) (SI Table S3 and Figure 2D). The small decrease in stiffness for **N1** above its *T*_g_ is common for highly cross-linked networks, such as thermosets.^50,51^

**Table 2:** Change in Network Storage Modulus and Calculated Crosslink Density for PU Thermosets **N1**–**N5**

thermoset network	Δ*E*′ (MPa)	cross-link density (*v*_e_) (M/m^3^)
N1	322 ± 110	170
N2	2268 ± 96	1105
N3	2378 ± 249	1191
N4	3042 ± 660	623
N5	2928 ± 283	667

Network cross-link density (*v*_e_) in M/m^3^ was determined from the rubbery modulus according to the following formula:^20^
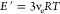
1where *E*′ is the storage modulus in the rubbery plateau above the *T*_g_, *R* is the universal gas constant (8.314 J K^–1^ mol^–1^), and *T* (at 85 °C) is the tempeature in Kelvin. As shown in Table 2, silyl-PUs **N4** and **N5** had a lower cross-link density than **N2** and **N3**, which corresponds to the longer aliphatic chains in the former, whereas the PU control (**N1**) had the lowest value of all at 170 M/m^3^. DMA analysis of networks **N6**–**N9** was not performed because a PU control with urea linkages could not be fabricated (see Section 2.1); however, we suspect these networks would produce similar results to **N2**–**N5** because they used the same network components.

### 2.2 Network Degradation via Chemical Stimuli

Poly(urethane) thermosets **N1**–**N9** were immersed in solutions of neat tetrahydrofuran (THF), 1.0 M tetrabutylammonium fluoride (TBAF) in THF, 1.0 M TBAF in acetone, and 0.5 M cesium fluoride (CsF) in THF under static conditions at room temperature to determine their degree and time of degradation. THF was used as a fluoride-free control to demonstrate the PUs are not degraded by an organic solvent, and as expected, no visible changes to **N1**–**N9** occurred after 1 week of immersion. The nonsilyl PU control (**N1**) also showed no visible change after immersion in both fluoride salt solutions for 24 h, thereby demonstrating it was not responsive to fluoride ion stimuli. This was expected for a network that resembles a traditional PU thermoset, as linear aliphatic carbamate linkages are not cleaved with fluoride ion at room temperature.^52^ All silyl-PUs with extended aliphatic chains (**N4** and **N5**, **N8** and **N9**) completely degraded (visually) after 6 h immersion in 1.0 M TBAF (THF), whereas **N3** required up to 24 h to visually degrade in the same solution. Small pieces (about 4–5 wt.%) of silyl-PU networks **N2**, **N6**, and **N7** remained after 24 h immersion in 1.0 M TBAF (THF), yet were visually degraded after 30–36 h. Immersion of **N2**–**N9** in 1.0 M TBAF (acetone) resulted in slightly slower visual degradation compared to immersion in 1.0 M TBAF (THF). Networks with extended aliphatic chains, such as **N5** and **N9**, disassembled within 6 h at room temperature, whereas all others disassembled within 24 h. The reduced time of network degradation in acetone is likely due to decreased swelling of the network compared to immersion in THF, in addition to reduced nucleophilicity of the fluoride ions due to the greater water content in acetone.^53^ None of the networks visually degraded after 24 h of static immersion in 0.5 M CsF (THF) at room temperature, and small pieces were visible even after 1 week of constant immersion, which can be attributed to the reduced concentration of fluoride ion and cesium fluoride’s limited solubility in THF. In general, silyl-PUs with urea linkages (**N6**–**N9**) showed minimal-to-no difference in time of visual disassembly compared to those with a slight excess of hydroxyl functionality (**N2**–**N5**), indicating that thermal and mechanical properties can be tailored without altering the time of network degradation.

**Figure 4:**
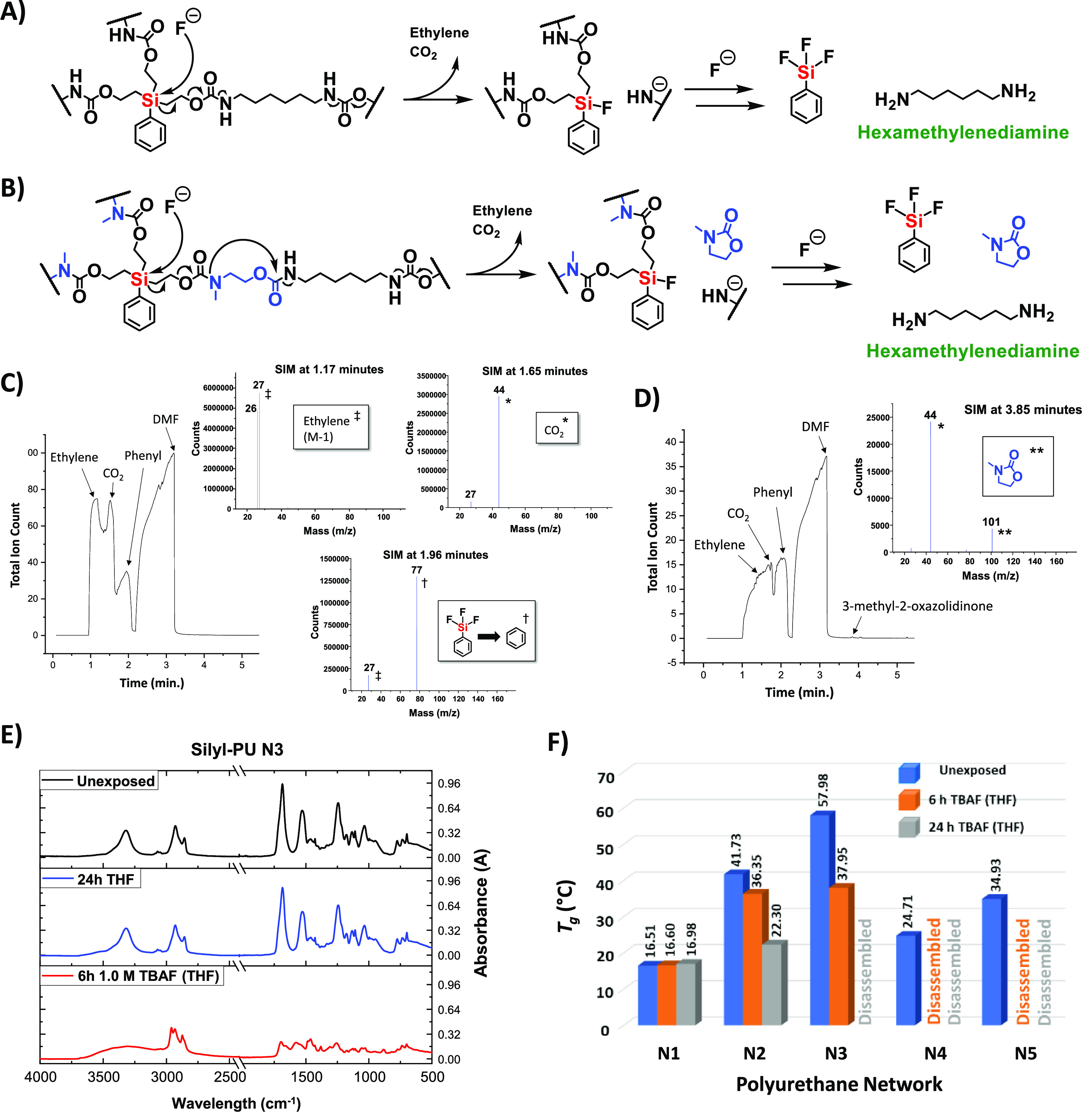
(A) Mechanism of degradation for silyl-PU **N3** and resulting byproducts. (B) Mechanism of degradation for silyl-PU **N5** and resulting byproducts. (C) HS-GC-MS of partially degraded **N3** in 1.0 M TBAF (DMF) with times of molecule elution and detected ions corresponding to ethylene, CO_2_, and phenyl (from trifluorophenysilane). (D) HS-GC-MS of partially degraded **N5** in 1.0 M TBAF (DMF) with times of molecule elution and detected ions corresponding to 3-methyl-2-oxazolidinone and coeluted CO_2_. (E) ATR-IR spectra of silyl-PU **N3**: (top) unexposed, (middle) exposed in THF for 24 h, and (bottom) exposed in 1.0 M TBAF (THF) for 6 h. (F) Comparison of glass transition temperatures for PUs **N1**–**N5** before and after static exposures: (blue bars) unexposed networks, (orange bars) PUs after 6 h of exposure in 1.0 M TBAF (THF), and (gray bars) PUs after 24 h of exposure in 1.0 M TBAF (THF).

Silyl-PU networks that possessed a phenyl group bound to silicon (e.g., **N3**) degraded faster than those with a methyl group (e.g., **N2**) due to increased electrophilicity at silicon.^40^ However, networks with extended aliphatic chains (e.g., **N4** and **N5**) likely degraded the fastest overall due to their reduced cross-link density and the greater entropic contributions resulting from increased bond cleavages and the generation of multiple degradation products. To prove this theory, the mechanism of network degradation was investigated for silyl-PUs **N3** and **N5**. As shown in Figures 4A,B, the mechanism of degradation for these networks occurred upon reaction of fluoride ion with the silyl trigger, followed by cleavage of the Si–C bond and subsequent cascading bond cleavages to generate ethylene, carbon dioxide, a fluoride bound adduct, and a primary amide ion. Three additions of fluoride ion at each trigger were required to completely disassemble the networks, whereby trifluorophenylsilane and hexamethylenediamine (HDMA) are generated as byproducts, including the formation of 3-methyl-2-oxazolidinone upon degradation of **N5**. Headspace sampling of partially degraded **N3** in 1.0 M TBAF in dimethylformamide (DMF), coupled with gas chromatography and mass spectrometry (HS-GC-MS) analysis using select ion monitoring (SIM), detected ethylene at 1.17 min, carbon dioxide at 1.65 min, and a phenyl group (from trifluorophenylsilane) at 1.96 min. (Figure 4C). Analysis of partially degraded **N5** in 1.0 M TBAF (DMF) detected the same molecules at similar times, in addition to 3-methyl-2-oxazolidione at 3.85 min. (Figure 4D and SI Figure S17). Furthermore, the visible generation of bubbles, presumably due to the evolution of ethylene and CO_2_, was observed upon immersion of these networks in a fluoride salt solution (SI Video 1). During degradation, we suspect that the primary amide ion of HMDA was protonated via a Hofmann Elimination with tetrabutylammonium ion.^54^ However, HMDA was not detected via HS-GC-MS due to its likelihood of being bound to the column, although it was recovered upon aqueous extractions (see Section 2.5). ^19^F NMR analysis of **N3** and **N5** after 48 and 24 h immersion in 1.0 M TBAF (THF), respectively, showed that only a single fluorine peak was observed for trifluorophenylsilane around −120 ppm (SI Figure S18), indicating that complete network degradation had occurred. Similarly, ^13^C NMR analysis of the solutions showed trifluorophenysilane as the only species present in the aromatic region of the spectra (SI Figure S19). These results confirm the mechanism of degradation for the silyl-PU networks, and are similar to those we reported for fluoride ion initiated disassembly of silyl-centered ethoxycarbonyl small molecules.^40^ Clean ^1^H and ^13^C NMR spectra of 3-methyl-oxazolidinone could not be obtained due to peak interference from tetrabutylammonium ion.

ATR-IR analysis of silyl-PUs **N2**–**N5** immersed in 1.0 M TBAF (THF) resulted in the disappearance of the urethane carbonyl stretches at 1680 cm^–1^ and amide II N–H bend at 1520 cm^–1^, while a new signal emerged for the Si–F bond at 880 cm^–1^ (Figure 4E and SI Figures S20–S22). Signals for the urethane functional groups were significantly reduced for **N2** and **N3** after 6 h, whereas spectra of **N4** and **N5** were not recorded at 6 h because the networks had visually completely degraded. The nonsilyl PU control (**N1**) network showed no bond position changes in the infrared region when immersed in static THF and 1.0 M TBAF (THF) for 24 h, although the absorption intensity for **N1** decreased due to the presence of TBAF upon drying (SI Figure S23). No spectroscopic changes were observed for **N2**–**N5** when immersed in THF for the same time period.

DSC was used to determine the *T*_g_ of **N1**–**N5** after static immersion in 1.0 M TBAF (THF) and 1.0 M TBAF (acetone) for 6 and 24 h at room temperature (Figure 4F and SI Figure S24A), including 0.5 M CsF (THF) for 24 h at room temperature (SI Figure S24B). The nonsilyl PU control (**N1**) demonstrated minimal change in *T*_g_ after immersion in all solutions, indicating no network degradation had occurred. Silyl-PU networks **N2** and **N3** showed a decrease in *T*_g_ of 5.38 and 20.03 °C, respectively, after 6 h in 1.0 M TBAF (THF), whereas the decrease in 1.0 M TBAF (acetone) for the same time period was 15.5 and 12.5 °C, respectively. After 24 h immersion, silyl-PU **N2** in 1.0 M TBAF (THF) was the only network that had not completely visually degraded, although the small remaining piece had a *T*_g_ of 22.3 °C, which was a decrease of 19.4 °C. Conversely, silyl-PU **N5** had completely visually degraded after 6 h in both solutions, thus a *T*_g_ could not be obtained, whereas **N4** visually degraded in only 1.0 M TBAF (THF) after 6 h. The small piece of **N4** that remained after 6 h in 1.0 M TBAF (acetone) had a *T*_g_ decrease of 29.6 °C (to −4.89 °C) and was a soft, gel-like material. After 24 h in 0.5 M CsF (THF) the decrease in *T*_g_ for networks **N2** and **N3** was approximately 50% to 20.7 and 29.4 °C, respectively. However, the results for **N4** and **N5** were even more pronounced at 26.2 and 25.9 °C, respectively, equating to a decrease of over 100% and 74.1%. These findings coincide with visible observations of silyl-PU network degradation and bond changes via ATR-IR spectra. Similar changes in *T*_g_ were observed for networks **N6**–**N9** upon immersion in TBAF solutions (SI Figure S25).

### 2.3 Hydrolytic Stability of Silyl-PU Networks

Network degradation of silyl-PUs **N3** and **N5** with a nonfluoride stimulus were evaluated by immersing in a static solution of 0.1 M tetrabutylammonium hydroxide (TBAOH) (isopropanol/methanol (10:1 v/v)) for 24 h at room temperature. This stimulus was selected for two reasons: (1) the counterion was identical to that in TBAF, thus negating substantial changes in ionization energy due to differences in cation radii, and (2) to determine if hydroxyl ion, which is generated in minute quantities by TBAF·H_2_O in THF, would hydrolyze bonds and degrade the networks. For comparison, **N3** and **N5** were also immersed in a static solution of 0.1 M TBAF (THF) for 24 h. No visible change in network size was observed from exposure in 0.1 M TBAOH, and as shown in SI Table S4, there was a nominal change in the thermal *T*_g_ of each network. This indicates that hydroxyl ion in organic solvents caused limited-to-no bond cleavages and was ineffective at degrading the silyl-PUs. Similarly, silyl-PU **N5** demonstrated a negligible visible and thermal *T*_g_ change after 24 h immersion in 1.0 M NaOH (aq.) and 1.0 M HCl (aq.), which was likely due to the limited miscibility of the network chains with water. In contrast, **N5** showed complete visual degradation in 0.1 M TBAF (THF) at 24 h.

The silyl-PU networks demonstrated exceptional hydrolytic stability at room temperature and retained consistent thermal and mechanical properties for over a year in the laboratory without visually degrading. This contrasts the purported stability of degradable networks that contain silyl ether linkages, which is seldom discussed in the literature. To demonstrate stability differences between the two chemistries we synthesized silyl ether triol **T6** from trimethoxymethylsilane, followed by synthesis of silyl ether PU network **N10** using a similar procedure as the silyl-PUs (see SI for details). The *T*_g_ of **N10** after formation was 53.6 °C. However, after several days of exposoure to laboratory conditions (i.e., 20–22 °C, 40–60% R.H.) this network began to degrade via hydrolytic cleavage of the silyl ether linkages, which was evidenced by the continued visual whitening, increased brittleness of the material, increased size of the Si–O–Si bands around 1100 cm^–1^, and increased –OH broadening around 3500 cm^–1^ (SI Figures S26A,B). Furthermore, immersion of **N10** in static THF for 24 h at room temperature resulted in network degradation with a resulting weight loss of 44.8% and a *T*_g_ decrease to −1.6 °C. This demonstrates the instability of a degradable PU network based on silyl ether linkages, which is unacceptable for real-world applications, whereas the silyl-PU networks (**N2**–**N9**) remained sufficiently robust under these conditions for a prolongued period of time.

Hygrothermal stability of **N1**, **N3**, and **N5** was evaluated by exposing the networks at 37.7 °C and 95% relative humidity for 5 days, followed by DSC analysis after drying in a vacuum oven. As shown in SI Table S5, the thermal *T*_g_ of each network was essentially unchanged, thereby indicating the silyl-PUs possessed equivalent hydrolytically stability compared to a traditional PU network with similar structure.

### 2.4 Selective Removal of Silyl-PU from Epoxy Network

**Figure 5:**
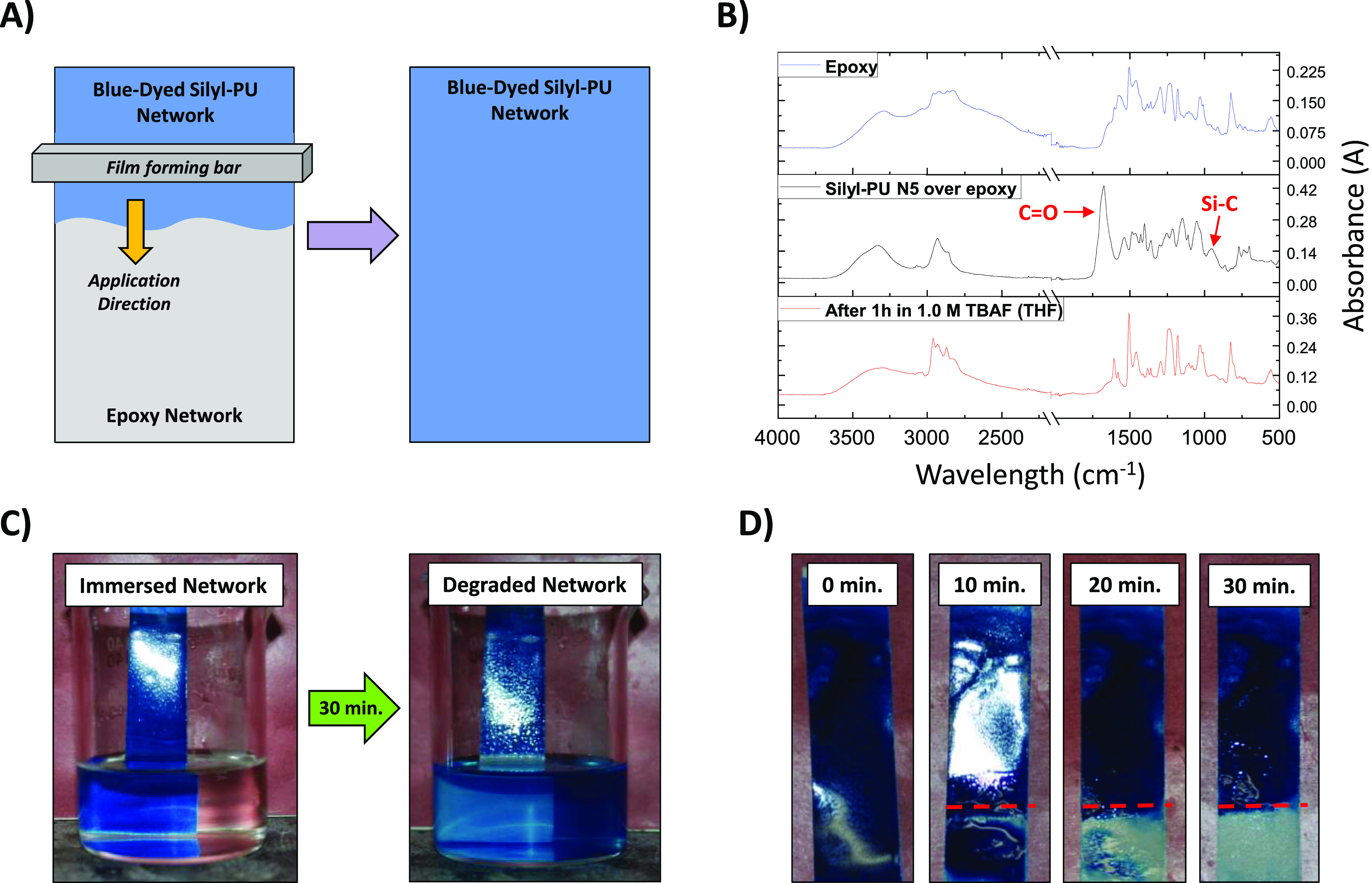
Selective removal of silyl-PU thermoset networks. (A) Illustration of blue-dyed silyl-PU application over epoxy network via film forming bar. (B) ATR-IR spectra of PU and epoxy networks: (top) epoxy on gold slide, (middle) silyl-PU **N5** on epoxy with red arrows pointing to peaks for carbonyl and Si–C bond stretches, and (bottom) gold slide after 1 h static immersion in 1.0 M TBAF (THF) showing **N5** removed and epoxy unchanged. (C) Static immersion of blue-dyed silyl-PU **N5** in 1.0 M TBAF (acetone) for 30 min and color change of surrounding solution. (D) Time-lapse removal of blue-dyed silyl-PU **N5** from epoxy after 10, 20, and 30 min of static immersion in 1.0 M TBAF (THF), where the sample at 30 min shows complete removal of **N5** and intact epoxy network. The red dashes indicate the level of sample immersion.

The selective removal of a strongly adhered polymeric network from an underlying network of similar or different chemical composition, without altering the chemical structure of the latter, has not been demonstrated in the literature and remains one of the reasons why degradable networks have yet to see commercial viability.^27^ To address this issue, a cross-linked epoxy-amine network (a.k.a. epoxy) was applied onto gold slides and pretreated aluminum panels via spin-coating or film forming bar, respectively (see SI for details). PU networks **N1**–**N5** were then spin-coated onto the epoxy-coated gold slides (SI Figure S27A,B), and blue-dyed versions of **N1** and **N5** were applied onto the epoxy-coated pretreated panels using a film forming bar (Figure 5A). All PU films showed similar IR signals as the cast versions. The coated gold slides were then immersed in a static solution of 1.0 M TBAF (THF) for 1 h at room temperature (SI Figure S27C), removed, allowed to air-dry, and analyzed both visually (SI Figure S27D) and via ATR-IR spectroscopy. Immersion of the nonsilyl PU control (**N1**) did not result in removal from the epoxy network or changes in IR signals (SI Figure S28A). However, the silyl-PUs were completely removed from the underlying epoxy network as shown by the disappearance of the carbonyl (∼1680 cm^–1^) and Si–C (∼780 cm^–1^) bond stretches, and only IR signals corresponding to the epoxy remained (Figure 5B and SI Figure S28B–D). No visible or IR changes to the epoxy network were observed, suggesting fluoride ion selectively degraded and removed only the silyl-PUs, but did not affect the chemical structure of the epoxy.

Blue-dyed versions of silyl-PU network **N5** and the nonsilyl PU control (**N1**) demonstrated excellent adhesion to the epoxy network on aluminum panels according to American Society for Testing Materials (ASTM) Method D3359 (SI Figures S29A,B; see Experimental Section for details).^55^ These samples were then exposed to static solutions of THF, acetone, 1.0 M TBAF (THF), and 1.0 M TBAF (acetone) at room temperature for up to 2 h. Exposure of both networks to neat THF and acetone resulted in no color change to the surrounding solution, indicating degradation had not occurred (SI Figures S30A,B and S31A,B). However, immersion of silyl-PU **N5** in 1.0 M TBAF (acetone) and 1.0 M TBAF (THF) for 30 min resulted in disassembly of the network and formation of a blue-colored solution (Figure 5C and SI Figure S30C). Time-lapse photographs of blue-dyed silyl-PU **N5** at 0, 10, 20, and 30 min exposures in 1.0 M TBAF (THF) showed that degrdation is relatively quick, removal is selective and complete, and the underlying epoxy network remained intact (Figure 5D). Exposure of the blue-dyed nonsilyl PU control (**N1**) over epoxy to the same fluoride ion solutions resulted in no color change to the surrounding solutions, even after 1 h of immersion (SI Figures S30D and S31C), indicating degradation did not occur. This demonstrated, once again, the ability of a silyl-PU to be selectively degraded and removed.

### 2.5 Generation of Reusable Molecules upon Degradation

**Figure 6:**
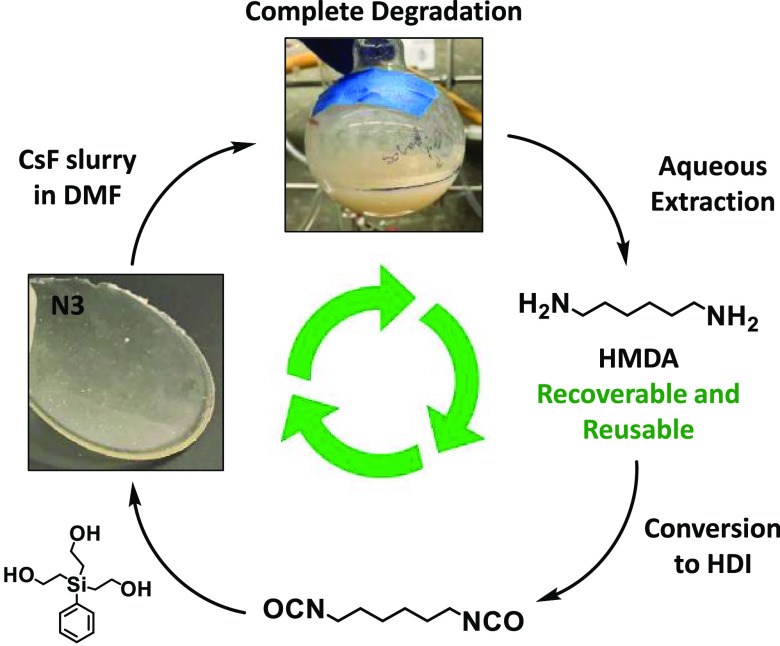
Illustration of partially recyclable silyl-PU: Degradation of silyl-PU **N3** via CsF in DMF, recovery of pure HMDA, reuse of HMDA to generate HDI, and reaction with silyl triol **T3** to reform silyl-PU **N3**.

A major component from the degradation of the silyl-PUs is hexamethylenediamine (HMDA) (Figure 4A,B), which has a global production of 2.1 million metric tons annually and an estimated value of more than $3 billion.^56,57^ HMDA is a valuable molecule used in numerous commercial applications, such as the synthesis of nylon-6,6 polymers,^58^ as a cross-linker for epoxy networks,^59^ and as a reactant with phosgene to form 1,6-hexamethylene diisocyanate (HDI) for use in PU networks.^60^ 3-methyl-2-oxazolidinone (MeOx), which is also a byproduct from the degradation of silyl-PUs **N4**, **N5**, **N8**, and **N9** (Figure 4B), is used as an electrolyte in lithium ion batteries.^61^ Although valuable, the commercial scale and usage of MeOx pales in comparison to HMDA. Herein, silyl-PU **N3** was fully degaded by stirring in a slurry of approximately 1.0 M CsF in DMF for 14 days, followed by aqueous extraction to recover 71.5% of pure HMDA (SI Figures S32B and S33B; see Experimental Section for details). The remainder of the HMDA was soluble in the organic layer as determined by ^1^H and ^19^F NMR analysis of the crude reaction mixture and a control extraction (SI Figures S32A and S33A). Attempts to recover pure HMDA from fully degraded **N3** in 1.0 M TBAF (THF) were unsuccessful due to the inability to remove all tetrabutyammonium ion. Although not conducted during this study, the recovered HMDA can be converted to HDI via established methods, such as reaction with phosgene or use of carbon dioxide through Mitsunobu Chemistry.^60,62^ This would be followed by reaction between HDI and silyl triol **T3** to reform silyl-PU network **N3** and complete the cycle (Figure 6), thereby demonstrating these networks are partially recyclable. Recovered HMDA can also be utilized to form other important materials as noted previously. The unique design of the silyl-PUs provides stability and properties that are similar to traditional PU themosets, while enabling relatively simple molecule recovery upon selective and complete degradation.

## 3 Conclusions

In summary, we have developed silyl-containing poly(urethane) thermoset networks from an aliphatic diisocyanate and synthesized silyl-centered triols with different chain lengths and structure. These triols enabled the generation of robust silyl-PU networks that exhibited greater stiffness and tensile strength compared to a nondegradable PU control with similar structure. A silyl-PU also demonstrated similar elongation to the control, even though the silyl-PUs possessed greater cross-link density. The silyl-PUs demonstrated excellent hydrolytic stability at ambient conditions and at elevated temperature and humidity, unlike a PU network based on silyl ether linkages, which degraded within a few days at ambient conditions. All silyl-PUs were activated via static fluoride salt solutions at room temperature, thereby visually degrading via cascading bond cleavages within a few hours of immersion. Silyl-PUs with phenyl-Si linkages degraded faster than those with methyl-Si due to increased electronegativity at silicon, and silyl-PUs with extended chains likely degraded faster than those with nonextended chains due to increased bond cleavages and reduced cross-link density. Furthermore, these silyl-PUs, as thin films, were selectively degraded and removed from a strongly adhered epoxy network without altering the chemical structure of the latter. Finally, the degradation of silyl-PUs generated reusable molecules, such as hexamethylenediamine, which was recovered in high yield via aqueous extractions. HMDA is produced in several million metric tons annually and is used in numerous commercial applications, thus recovery and reuse of molecules from degraded silyl-PUs can engender new materials to reduce environmental waste compared to single-use, nondegradable PU thermosets. Silyl-PUs have potential applications in packaging materials, high-performance coatings, composites, and rigid plastics. Additionally, the ability to effectively remove a silyl-PU from a nonmetallic substrate, without damaging the latter, may enable vital and sensitive substrates (e.g., anticorrosive primers, carbon-fiber reinforced composites) to remain both intact and undamaged.

## 4 Experimental Section

### 4.1 Synthesis of Nitrilotris(ethane-2-1-diyl) tris((2-hydroxyethyl)methyl)carbamate) (T1)

Triethanolamine (4.4 mL, 33.2 mmol) and triethylamine (32.4 mL, 232.4 mmol) were added to a 500 mL round-bottom flask containing 300 mL of dry acetonitrile. *N*,*N*’-disuccinimidyl carbonate (34.0 g, 132.8 mmol) was then added to the flask with a stir bar and allowed to stir for 16 h at room temperature. The reaction mixture was concentrated in vacuo to afford a yellow oil. The oil was suspended in deionized water (100 mL) and extracted using CHCl_3_ (3 × 50 mL). The organic layers were combined and concentrated in vacuo to afford a yellow oil. The oil was dissolved in dry acetonitrile (300 mL). Triethylamine (27.8 mL, 199.5 mmol) was added to the flask, followed by *N*-methylethanolamine (10.7 mL, 132.8 mmol). The reaction mixture was stirred at room temperature for 16 h. The reaction mixture was concentrated in vacuo to afford a yellow oil. Purification by column chromotography (9:1 CH_2_Cl_2_:CH_3_OH) afforded **T1** as a yellow oil (8.9 g, 59.5% yield). ^1^H NMR (400 MHz, DMSO-*d*_6_, 25 °C): δ = 4.68 (s, 3H, H1), 3.98 (t, *J* = 5.8 Hz, 6H, H5), 3.47 (m, 6H, H2), 3.23 (t, *J* = 6.0 Hz, 6H, H6), 2.85 (s, 9H, H4), 2.74 (m, 6H, H3). ^13^C NMR (100 MHz, DMSO-*d*_6_, 25 °C): δ = 156.11 (C4), 59.55 (C1), 59.92 (C5), 53.89 (C6), 51.40 (C2), 35.82 (C3). HRMS (ESI) *m*/*z*: [M + H] calcd for C_18_H_36_O_9_N_4_: 453.2561; found 453.2574. The ^1^H and ^13^C NMR spectra for **T1** are shown in SI Figures S1 and S2.

### 4.2 Synthesis of (phenylsilanetriyl)tris(ethane-2,1-diyl) tris((2-hydroxyethyl)(methyl)carbamate) (T5)

2,2′,2″-(Phenylsilanetriyl)tris(ethan-1-ol) (**T4**) (9.98 g, 42.0 mmol) and triethylamine (34.0 mL, 243.9 mmol) were added to a 500 mL round-bottom flask containing dry acetonitrile (200 mL). *N*,*N*’-disuccinimidyl carbonate (42.5 g, 165.9 mmol) was then added to the flask with a stir bar and allowed to stir for 16 h at room temperature. The reaction mixture was concentrated in vacuo to afford a yellow powder. The powder was dissolved in chloroform (200 mL) and washed with a saturated aqueous NaCl solution (3 × 50 mL). The organic layer was concentrated in vacuo to afford a yellow powder. The powder was dissolved in acetonitrile (200 mL). Triethylamine (29.3 mL, 210.0 mmol) was added to the flask, followed by *N*-methylethanolamine (13.5 mL, 168.0 mmol). The reaction mixture was stirred at room temperature for 16 h. The mixture was concentrated in vacuo to afford a yellow oil. Purification by column chromatography (9:1 CH_2_Cl_2_:CH_3_OH) afforded **T5** as a clear, colorless oil (8.8 g, 72.6% yield). ^1^H NMR (400 MHz, DMSO-*d*_6_, 25 °C): δ = 7.56 (m, 2H, H8), 7.41–7.39 (m, 3H, H7 and H9), 4.65 (m, 3H, H1), 4.08 (t, *J* = 7.8 Hz, 6H, H5), 3.44 (q, *J* = 12.4, 6 Hz, 6H, H2), 3.19 (m, 6H, H3), 2.82 (s, 9H, H4), 1.33 (t, *J* = 7.4 Hz, 6H, H6). ^13^C NMR (100 MHz, DMSO-*d*_6_, 25 °C): δ = 156.06 (C4), 134.22 (C8), 129.99 (C7), 128.50 (C9), 128.40 (C10), 62.32 (C5), 59.49 (C1), 51.30 (C2), 53.62 (C3), 14.09 (C6). HRMS (DSA) *m*/*z*: [M + H] calcd for C_24_H_41_N_3_O_9_Si: 544.2684; found 544.2697. The ^1^H and ^13^C NMR spectra for **T5** are shown in SI Figures S9 and S10.

### 4.3 Synthesis of Non-Silyl PU Control Network (N1)

The nonsilyl-containing PU control (**N1**) network was synthesized by adding nitrilotris(ethane-2–1-diyl) tris((2-hydroxyethyl)methyl)carbamate) (**T1**) (4.00 g, 0.0265 mol OH) to a 25 mL round-bottom flask, followed by the addition of dry ethyl acetate (3.16 g). Next, 1,6-hexamethylene diisocyanate (HDI) (2.12 g, 0.0252 mol NCO) was added, followed by stirring and heating at 60 °C for 1 h until the mixture became clear. The solution was poured into a 2.5-in. aluminum weighing pan, the solvent was allowed to evaporate for 30–60 min, then the pan was placed into the oven at 60 °C for 36 h to form the solid network. Network thickness was 1–2 mm.

### 4.4 Synthesis of Silyl-PU Network N5

Silyl-containing poly(urethane) **N5** was synthesized by adding (phenylsilanetriyl)tris(ethane-2,1-diyl) tris((2-hydroxyethyl)(methyl)carbamate) (**T5**) (3.00 g, 0.0165 mol OH) to a 25 mL round-bottom flask, followed by the addition of dry ethyl acetate (2.22 g). Next, HDI (1.32 g, 0.0157 mol NCO) was added, followed by stirring and heating the solution at 60 °C for 1 h. The solution was poured into a 2.5-in. aluminum weighing pan, the solvent was allowed to evaporate for 30–60 min, then the pan was placed into the oven at 60 °C for 36 h to form the solid network. Network thickness was 1–2 mm.

### 4.5 Synthesis of Blue-Dyed PUs N1 and N5 and Application on Epoxy-Coated Aluminum Panels

A blue-dyed version of the nonsilyl-containing PU control (**N1**) was synthesized by adding nitrilotris(ethane-2-1-diyl) tris((2-hydroxyethyl)methyl)carbamate) (**T1**) (3.98 g, 0.0264 mol OH) to a small plastic cup, followed by the addition of dry ethyl acetate (1.09 g). Next, HDI (2.12 g, 0.0252 mol NCO) was added, followed by the addition of Chroma-Chem 850–7340 phthalo blue green-shade colorant (0.87 g) and a 10 wt.% solution of dibutyltin dilaurate (DBTDL) in dry ethyl acetate (0.03 g). The blue-colored solution was then mixed by hand for 5–10 min, followed by applying onto 24-h cured epoxy-coated aluminum panels with a 6 mil (152.4 μm) film forming bar. The samples were allowed to cross-link under normal laboratory conditions for at least 7 days. The resulting film thickness of the blue-dyed version of PU **N1** was an average of 65 μm.

The blue-dyed version of silyl-containing PU **N5** was synthesized by adding (phenylsilanetriyl)tris(ethane-2,1-diyl) tris((2-hydroxyethyl)(methyl)carbamate) (**T5**) (4.85 g, 0.0267 mol OH) to a small plastic cup, followed by the addition of dry ethyl acetate (1.25 g). Next, HDI (2.15 g, 0.0255 mol NCO) was added, followed by the addition of Chroma-Chem 850–7340 phthalo blue green-shade colorant (1.00 g) and a 10 wt.% solution of DBTDL in dry ethyl acetate (0.04 g). The blue-colored solution was then mixed by hand for 5–10 min, followed by applying onto the 24-h cured epoxy-coated aluminum panels with a 6 mil (152.4 μm) film forming bar. The samples were allowed to cure under normal laboratory conditions for at least 7 days. The resulting film thickness of the blue-dyed version of silyl-PU **N5** was an average of 65 μm.

### 4.6 Degradation of Silyl-PU Network N3 and Recovery of Pure HMDA

Pieces of silyl-PU network **N3** (5.04 g) were added to a 500 mL round-bottom flask, followed by DMF (60 mL) and CsF (9.11 g, 0.060 mol) to form a suspension (SI Figure S31). The suspension was then stirred for 2 weeks. During this period, gases, presumably ethylene and CO_2_ based on the proposed mechanism of chain disassembly, visibly evolved from the flask as network degradation proceeded. After 2 weeks, the network had visibly degraded. Water (25 mL) was slowly added to the suspension to protonate any residual amide ion of hexamethylenediamine (HMDA) that formed during the degradation process, and the solution became hot. Solvents were removed under reduced pressure resulting in a yellow powder. The resulting powder was suspended in chloroform (25 mL) and vacuum filtered. The chloroform filtrate was collected and washed with water (3 × 25 mL). The aqueous washes were combined and water was removed under reduced pressure yielding a white solid (0.79 g). The remaining organic layer was washed with water (3 × 25 mL), and the aqueous fractions were collected. The water was removed under reduced pressure to yield a white solid (0.44 g). The total amount of HMDA recovered was 1.23 g for a yield of 71.5%. SI Figure S32 shows the ^1^H NMR purity of the recovered HMDA versus that of the crude mixture, whereas SI Figure S33 shows the ^19^F NMR purity of the recovered HMDA versus the crude mixture.

An aqueous extraction of pure HMDA was performed as a control to determine the ideal% recovery. For this experiment, HMDA (0.610 g) was dissolved in water (25 mL), followed by washing with chloroform (3 × 25 mL). The aqueous layer was separated and the water was removed under reduced pressure to yield 0.320 g of pure HMDA, which equates to 52.4% recovery. The chloroform layer was then washed with water (25 mL) a second time, followed by separating the aqueous layer and removing the water under reduced pressure to yield 0.190 g pure HMDA. The combined recovery was 83.6%, which is similar to the amount recovered from the CsF slurry degradation of silyl-PU **N3**.

## Supporting Information Available

Materials and methods, synthetic procedures, and characterization data for other triols, including synthetic procedures and experiments for all other networks (PDF)
Video S1 (MOV)

## Supplementary Material

am2c00485_si_001.pdf

am2c00485_si_002.mov

## Abbreviations

Silyl-PU: silyl-containing poly(urethane)
PU: poly(urethane)
HDI: 1,6-hexamethylene diisocyanate
ATR-IR: attenuated total reflectance infrared spectroscopy
TGA: thermogravimetric analysis
DSC: differential scanning calorimetry
DMA: dynamic mechanical analysis
*T*_g_: glass transition temperature
THF: tetrahydrofuran
TBAF: tetrabutylammonium fluoride
CsF: cesium fluoride
MeOx: 3-methyl-2-oxazolidinone
HMDA: hexamethylenediamine
NMR: nuclear magnetic resonance
DMF: dimethylformamide
