# Corrections to “Hydrolytically Stable and Thermo-Mechanically Tunable Poly(Urethane) Thermoset Networks that Selectively Degrade and Generate Reusable Molecules”

**DOI:** 10.1021/acsami.2c18024

**Published:** 2022-10-24

**Authors:** Keith B. Sutyak, Erick B. Iezzi, Grant C. Daniels, Eugene Camerino

On page 22 410 of the original article, the reported units for cross-link density and the temperature used to calculate these values are incorrect. Specifically, the units reported for cross-link density in Table 2, paragraph three, and paragraph four should be mol/m^3^, not M/m^3^, and the reported temperature for calculating these values should be 80 °C, not 85 °C, as stated in paragraph four. The calculated cross-link density values in the third column of Table 2 and discussed within the article are correct. These corrections do not alter the conclusions of the work, although the units of M/m^3^ are incorrect and misleading to someone who is not familiar with calculating cross-link density values for polymeric networks.

**Table 2:** Change in Network Storage Modulus and Calculated Cross-link Density for PU Thermosets N1–N5

thermoset network	Δ*E*′ (MPa)	cross-link density (*v*_e_) (mol/m^3^)
N1	322 ± 110	170
N2	2268 ± 96	1105
N3	2378 ± 249	1191
N4	3042 ± 660	623
N5	2928 ± 283	667

