# Supplementary material for: Hydrolytically Stable and Thermo-Mechanically Tunable Poly(Urethane) Thermoset Networks that Selectively Degrade and Generate Reusable Molecules
Source: ACS Appl Mater Interfaces. 2022 May 3;14(19):22407–17. doi: 10.1021/acsami.2c00485 (PMC9242536; doi:10.1021/acsami.2c00485)
Supplement: Supplementary file 1 — am2c00485_si_001.pdf [file am2c00485_si_001.pdf]

Supporting Information

# Hydrolytically Stable and Thermo-Mechanically Tunable Poly(Urethane) Thermoset Networks that Selectively Degrade and Generate Reusable Molecules

Keith B. Sutyak[b], Erick B. Iezzi[a,*], Grant C. Daniels[a], and Eugene Camerino[a]

[a]U.S. Naval Research Laboratory, Chemistry Division, Washington, DC 20375, USA
[b]ASEE Post-Doctoral Fellow, U.S. Naval Research Laboratory, Chemistry Division, Washington, DC 20375, USA
*Corresponding author. Email address: erick.iezzi@nrl.navy.mil

## Table of Contents


1. Materials and Methods........................................................................................................................... 3

2. Synthesis of Non-Silyl and Silyl-Containing Triol Crosslinkers.......................................................................... 5

3. Synthesis of Poly(urethane) Networks with Excess Hydroxyl Functionality (N2-N4)......................................21

4. Synthesis of Poly(urethane) Networks with Excess Isocyanate Functionality (N6-N9)……………………..…....22

5. Synthesis of Silyl Ether Poly(urethane) Network with Excess Hydroxyl Functionality (N10)…...………….......22

6. Synthesis of Epoxy-Amine Network…………...……………………..…………….……………………….23

7. Synthesis of Poly(urethane) Networks (N1-N5) over Epoxy-Coated Gold Slides…………...……………........23

8. Tables S1-S5 and Figures S11-S33…………………………………………….......……….……….…….…...23

9. References………………………………………………………………………………………….……..…49

## 1. Materials and Methods

Methyltrivinylsilane, trichloropentylsilane, triethanolamine, triethylamine (TEA), *N,N'*-disuccinimidyl carbonate (DSC), *N*-methylethanolamine, 0.5 M 9-borabicyclo[3.3.1]nonane (9-BBN) solution in tetrahydrofuran (THF), 1.0 M vinyl magnesium bromide solution in THF, dibutyltin dilaurate (DBTDL), ethyl acetate, xylenes, deuterated chloroform ($CDCl_3$), and deuterated DMSO (DMSO-$d_6$) were purchased from Sigma-Aldrich. Hydrogen peroxide solution (30 Wt.% in water) and 0.1 M tetrabutyammonium hydroxide (TBAOH) in isopropanol/methanol (10:1 v/v) were purchased from Fisher. Tetrabutylammonium fluoride (TBAF) hydrate (TBAF·$H_2O$) was purchased from TCI America. The aliphatic isocyanate, 1,6-hexamethylene diisocyanate (HDI), was purchased from TCI America. Bisphenol A / epichlorohydrin difunctional epoxy resin was purchased from Miller-Stevenson as Epon 828. All chemicals were used as received without any further purification. Solvents were used as received, except for ethyl acetate, which was dried under molecular sieves for 24 hours.

Gold slides (Cr 50Å, Au 1000Å, size 2.54 cm x 2.54 cm x 0.157 cm) were purchased from Evaporative Metal Films (EMF) Corporation. The 7.62 cm x 15.24 cm x 0.051 cm aluminum alloy panels were purchased from Q-Lab Corporation, followed by chromic acid anodization by Almag Plating Corporation. Chroma-Chem 850-7340 Phthalo Blue Green-shade colorant was purchased from Chromaflo Technologies. Thin layer chromotography (TLC) was performed on EMD silica gel 60 F254 plates from Sigma-Aldrich, while column chromotography was performed using flash grade silica gel ($SiO_2$, 32-63 μm) from Sigma-Aldrich.

NMR spectra were performed on a Bruker 400 MHz NMR spectrometer and worked-up using Topspin. $^{13}$C NMR spectra were recorded at 100 MHz. $^{19}$F NMR spectra were recorded at 376 MHz. *J* coupling values are represented in Hz. NMR data is reported as follows: chemical shift (δ), multiplicity (bs = broad singlet, bt = broad triplet, singlet = singlet, d = doublet, t = triplet, q = quartet), coupling constant(s) in Hz, and integration. High-resolution mass spectroscopy (HRMS) was performed on a PerkinElmer AXION 2 time of flight (TOF) with direct sample analysis (DSA) source in positive mode.

Gel fraction analysis was performed on each sample by weighing, soaking in THF for 24 hours, drying *in vacuo* for 24 hours, and weighing again. Gel fraction was determined as the fraction of the final mass over the initial mass.

Attenuated Total Reflection Fourier Transform Infrared (ATR-FTIR) spectroscopy was performed on a Nicolet iS50-FT-IR with iS50 ATR attachment equipped and a diamond ATR crystal from Thermo Scientific with 64 scans compiled for each spectrum. Spectra were recorded from 500-4000 cm$^{-1}$ with a resolution of 2 cm$^{-1}$, and were analyzed using the Nicolet OMNIC software suite.

Thermal analysis was performed on TA Instruments Discovery Differential Scanning Calorimeter (DSC) to determine glass transition temperature ($T_g$). Two successive ramps were performed from -50 °C to 170 °C at a rate of 10 °C per minute, from which measurements were made on the second run. Samples were run in triplicate, or greater, and standard deviation was provided for each set. Thermogravimetric analysis (TGA) was performed on a TA Instruments Discovery TGA at a heating rate of 10 °C per minute under $N_2$ from room temperature to 700

°C.  Degradation onset temperature was assigned at the temperature at which 90% mass remained.  TA Instruments Trios software was used to analyze DSC and TGA data.

Tensile testing of dog bone shaped samples (3.6 cm length x 1.4 cm width x about 1 mm thickness) was performed with a Texture Technologies TA.XT Plus Texture Analyzer at 21 °C to determine Young's modulus, ultimate tensile strength at break, and percent elongation at break.  Samples were run at 10 mm per minute and had an area of 2.8 $cm^2$.  All samples were performed in at least triplicate to obtain standard deviation.

Dynamic mechanical analysis (DMA) of dog bond shaped samples was performed using a TA Instruments Q-800.  The method equilibrated at -25 °C and held for 5 minutes, followed by a 5 °C ramp to 100 °C with a data acquisition of 1 Hz.  Network $T_g$ was calculated from the Tan Delta (Tan δ) (E′′/E′), which is the ratio of loss modulus (E′′) to the storage modulus (E′).

Headspace sampling with gas chromatography and mass spectrometry (HS-GC-MS) analysis of partially degraded networks was performed with an Agilent 7696A Headspace Analyzer connected to an Agilent 7890B GC (with a Restek Rxi-5ms 15 m and 0.25 mm ID column) and Agilent 5977B MS.  The headspace (HS) method used an oven temperature of 90 °C, a sample loop temperature of 90 °C, and a transfer line temperature of 110 °C.  The vial of partially degraded network was equilibrated for 20 minutes before injection and the sample loop was filled for 0.5 minutes.  The GC-MS method has an inlet temperature of 200 °C, initial oven temperature of 40 °C, which is held for 0.5 minutes, followed by a ramp of 20°C per minute to 250 °C, whereupon the temperature was held for 1 minute with a flow rate of 2 mL per minute.  The mass spectrometer used electron impact ionization with a mass scan range of 30-500 m/z.  After doing an initial scan the MS was changed to select ion monitoring (SIM) to provide a clearer chromatograph.  The selected ions where 26 and 27 for ethylene, 44 for carbon dioxide ($CO_2$), 77 and 162 for trifluorophenylsilane, and 101 for 3-methyl-2-oxazolidinone.  For these experiments, 1.0 M TBAF in dimethylformamide (DMF) was used instead of THF to reduce solvent volatility and prevent obscuring molecules of interest.

Hygrothermal exposure of networks was performed using a Thermotron S4-8200 chamber at 37.7 °C and 95% relative humidity for 5 days, followed by drying of networks *in vacuo* at 60 °C for 24 hours.

Immersion experiments in stimuli solutions (e.g. fluoride salts) were conducted under static conditions at room temperature using pieces of networks with dimensions of approximately 9 mm length by approximately 6 mm width by approximately 1 mm thickness, unless otherwise noted.

## 2. Synthesis of Non-Silyl and Silyl-Containing Triol Crosslinkers

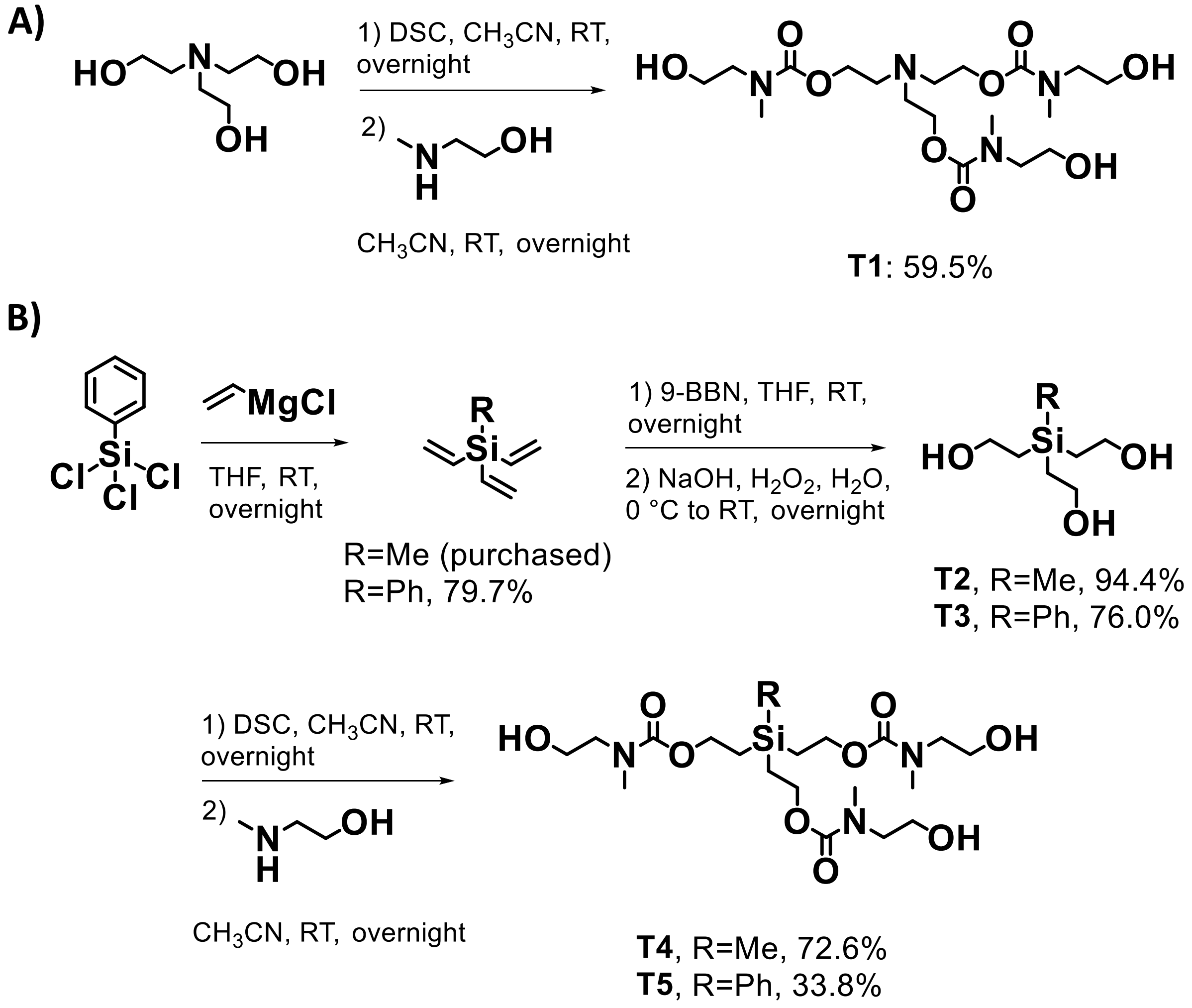


**Scheme S1.** Synthesis of triol crosslinkers for use in PU networks. (A) Synthesis of extended chain triethanolamine (**T1**). (B) Synthesis of non-extended methyl and phenyl functional silyl triols (**T2-T3**) and extended chain methyl and phenyl functional silyl triols (**T4-T5**).

Synthesis of extended chains silyl triols containing N-methylethanolamine was achieved using known methods to produce asymmetric carbamates through alkoxycarbonylation of an amine.[1]

**Phenyltrivinylsilane**:

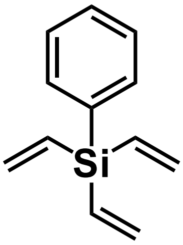

Phenyltrichlorosilane (8.6 mL, 53.0 mmol) was added to a 500 mL round bottom flask and cooled to 0 °C using an ice bath. A 1.0 M vinylmagnesium bromide solution in THF (185.5 mL, 185.5 mmol) was added to the flask while stirring. After 1 hour the cooling bath was removed and the mixture was stirred at room temperature for 16 hours. Saturated aqueous ammonium chloride (50 mL) was added, followed by dilution with deionized water (100 mL). The resulting organic layer was isolated. The aqueous layer was extracted with dichloromethane (3 x 50 mL). The organic layers were combined and dried with $MgSO_4$, filtered, and concentrated *in vacuo* to give an oil. Purification by column chromatography (hexanes) afforded the product as a colorless oil (7.9 g, 79.7% yield). $^{1}$H NMR (400 MHz, $CDCl_3$, 25 °C): $\delta$ = 7.59-7.61 (m, 2H), 7.41-7.43 (m, 3H), 6.38 (dd, $J$ = 20.0, 12.0 Hz, 3H), 6.25 (dd, $J$ = 16.0, 4.0 Hz, 3H), 5.87 (dd, 20.0, 4.0 Hz, 3H). $^{13}$C NMR (100 MHz, $CDCl_3$, 25 °C): $\delta$ = 136.06, 135.23, 134.55, 133.99, 129.59, 128.02. No mass peak could be found using ESI-MS. The spectroscopic data is in agreement with previously reported data in the literature.[2]

**Nitrilotris(ethane-2-1-diyl) tris((2-hydroxyethyl)methyl)carbamate) (T1):**

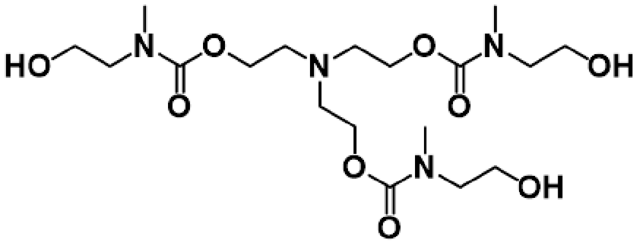


Triethanolamine (4.4 mL, 33.2 mmol) and triethylamine (32.4 mL, 232.4 mmol) were added to a 500 mL round bottom flask containing 300 mL dry acetonitrile. *N,N'*-disuccinimidyl carbonate (34.0 g, 132.8 mmol) was then added to the flask with a stir bar and allowed to stir for 16 hours at room temperature. The reaction mixture was concentrated *in vacuo* to afford a yellow oil. The oil was suspended in deionized water (100 mL) and extracted using $CHCl_3$ (3 x 50 mL). The organic layers were combined and concentrated *in vacuo* to afford a yellow oil. The oil was dissolved in dry acetonitrile (300 mL). Triethylamine (27.8 mL, 199.5 mmol) was added to the flask, followed by *N*-methylethanolamine (10.7 mL, 132.8 mmol). The reaction mixture was stirred at room temperature for 16 hours. The reaction mixture was concentrated *in vacuo* to afford a yellow oil. Purification by column chromotography (9:1 $CH_2Cl_2$:$CH_3OH$) afforded **T1** as a yellow oil (8.9 grams, 59.5% yield). $^{1}$H NMR (400 MHz, DMSO, 25 °C): δ = 4.68 (s, 3H, H1), 3.98 (t, J= 5.8 Hz, 6H, H5), 3.47 (m, 6H, H2), 3.23 (t, J= 6.0 Hz, 6H, H6), 2.85 (s, 9H, H4), 2.74 (m, 6H, H3). $^{13}$C NMR (100 MHz, DMSO, 25 °C): δ = 156.11 (C4), 59.55 (C1), 59.92 (C5), 53.89 (C6), 51.40 (C2), 35.82 (C3). HRMS (ESI) *m/z*: [M+H] calcd for $C_{18}H_{36}O_9N_4$: 453.2561; found 453.2574. The $^{1}$H and $^{13}$C NMR spectra are shown in Figure S1 and S2.

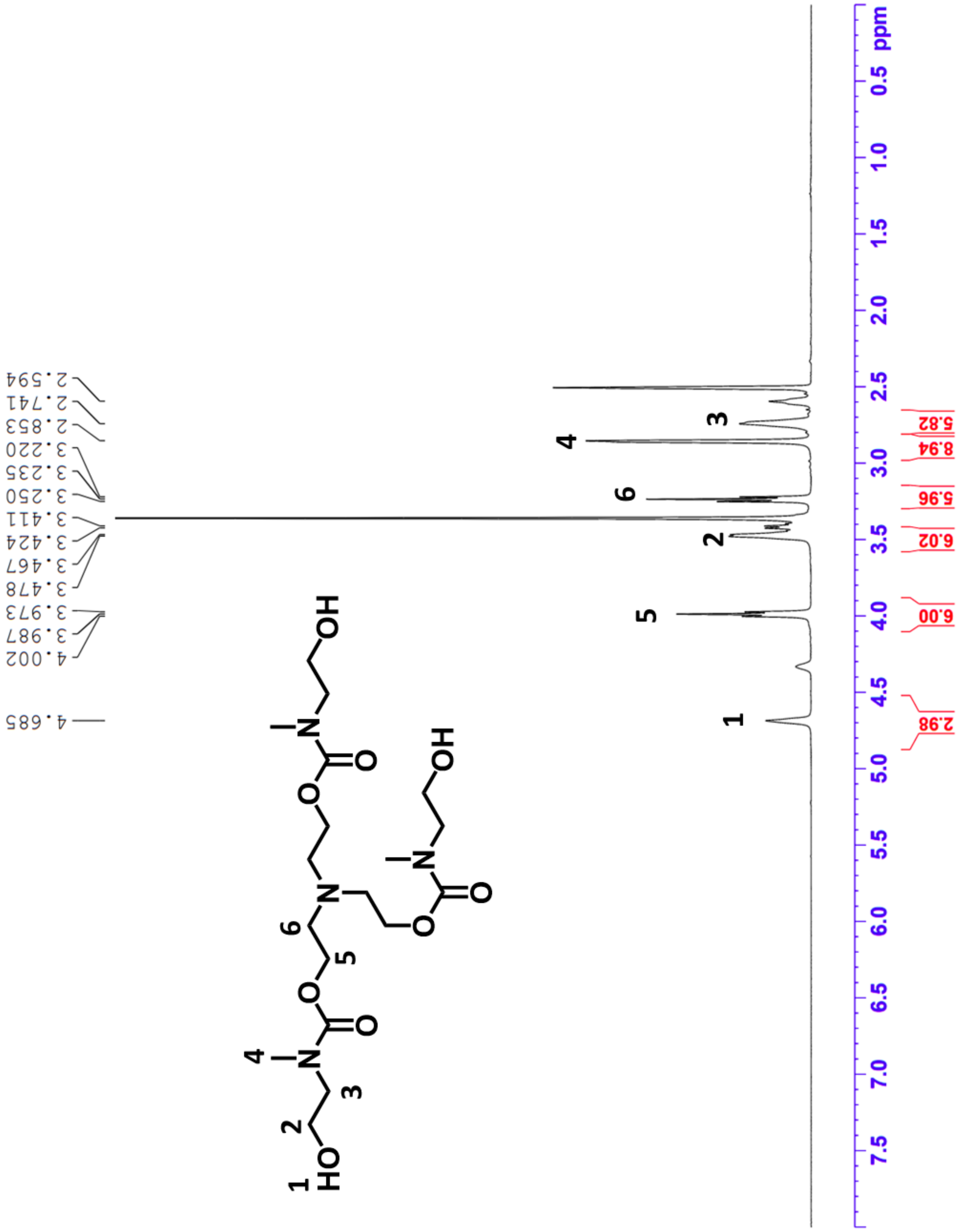


**Figure S1.** $^1$H NMR spectrum of nitrilotris(ethane-2-1-diyl) tris((2-hydroxyethyl)methyl)carbamate) (**T1**).

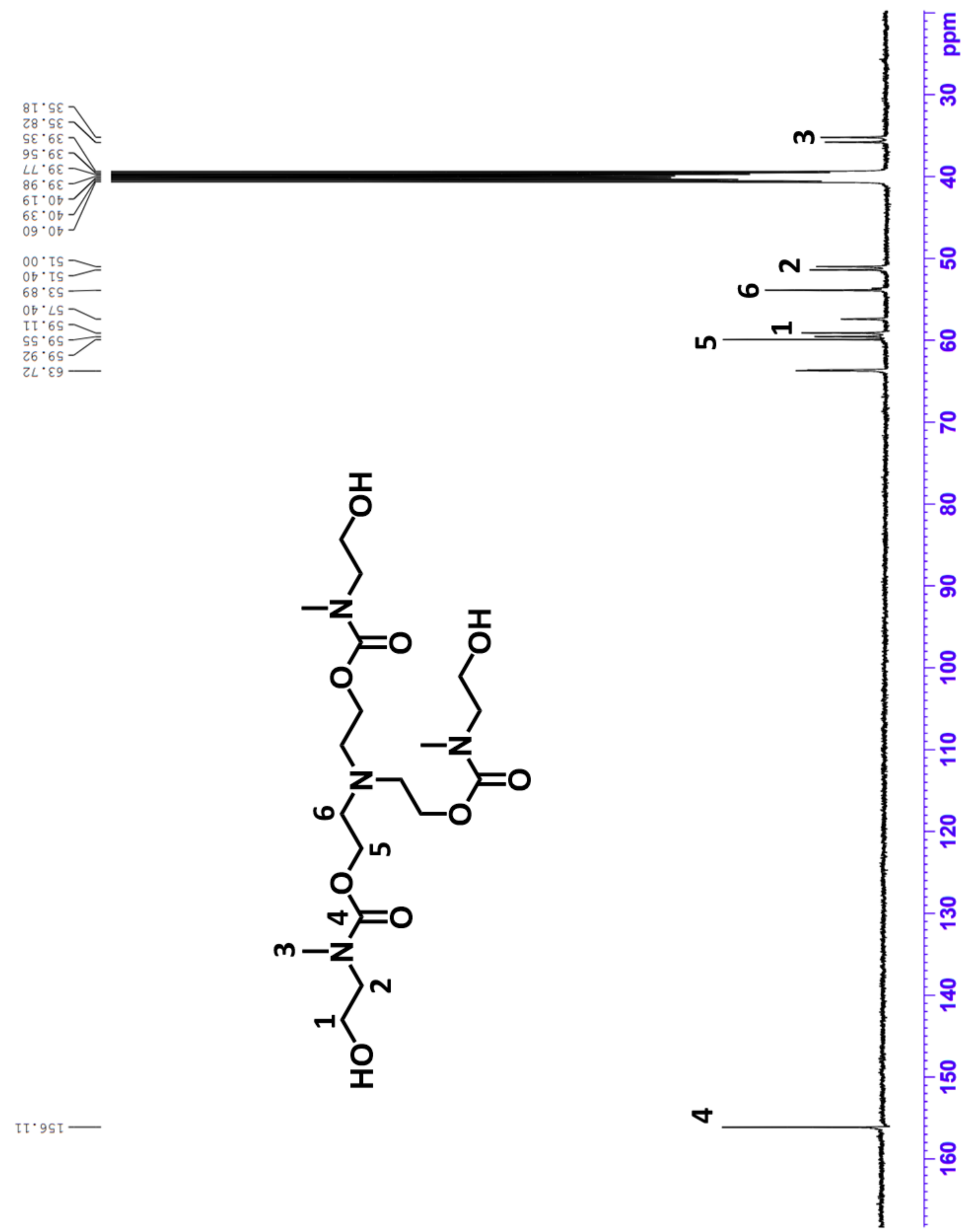


**Figure S2.** $^{13}$C NMR spectrum of nitrilotris(ethane-2-1-diyl) tris((2-hydroxyethyl)methyl)carbamate) (**T1**).

**2,2′,2′′-(Methylsilanetriyl)tris(ethan-1-ol) (T2)**:

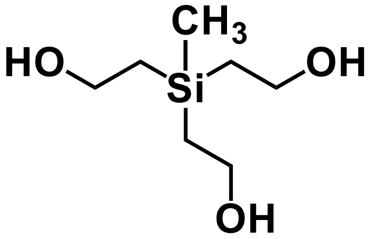


A solution of 0.5 M 9-borabicyclo[3.3.1]nonane in THF (491 mL, 245.5 mmol) was added to a 1 L three-neck round bottom flask and cooled to 0 °C using an ice bath. Methyltrivinylsilane (10.6 mL, 65.4 mmol) was added drop-wise to the flask over a period of 15 minutes after which the ice bath was removed and the reaction mixture was stirred for 4 hours. After 4 hours, water (50 mL) was added to the flask, followed by a 3.0 M aqueous sodium hydroxide solution (50 mL). Subsequently, Aqueous hydrogen peroxide solution (30 Wt.%, 50 mL) was added dropwise over 1 hour while being kept at 0 °C. The reaction mixture was heated to reflux and stirred for 3 hours. Upon cooling to room temperature, the reaction mixture was extracted with ethyl acetate (150 mL). The organic layer was dried using magnesium sulfate and concentrated *in vacuo* to afford a clear oil. Purification by column chromatography on silica gel (9:1 $CH_2Cl_2$:$CH_3OH$) furnished **T2** as a colorless oil (11.0 g, 94.4% yield). $^1H$ NMR (400 MHz, DMSO-$d_6$, 25 °C): $\delta$ = 4.33 (t, $J$ = 4.8 Hz, 3H, H1), 3.48 (m, 6H, H2), 0.81 (t, $J$ = 8.2 Hz, 6H, H3), -0.04 (s, 3H, H4). $^{13}C$ NMR (100 MHz, DMSO-$d_6$, 25 °C): $\delta$ = 58.06 (C1), 19.76 (C2), -3.71 (C3). HRMS (DSA) *m/z*: [M+H] calcd for $C_7H_{18}O_3Si$: 179.1097; found 179.1100. The $^1H$ and $^{13}C$ NMR spectra are shown in Figures S3 and S4.

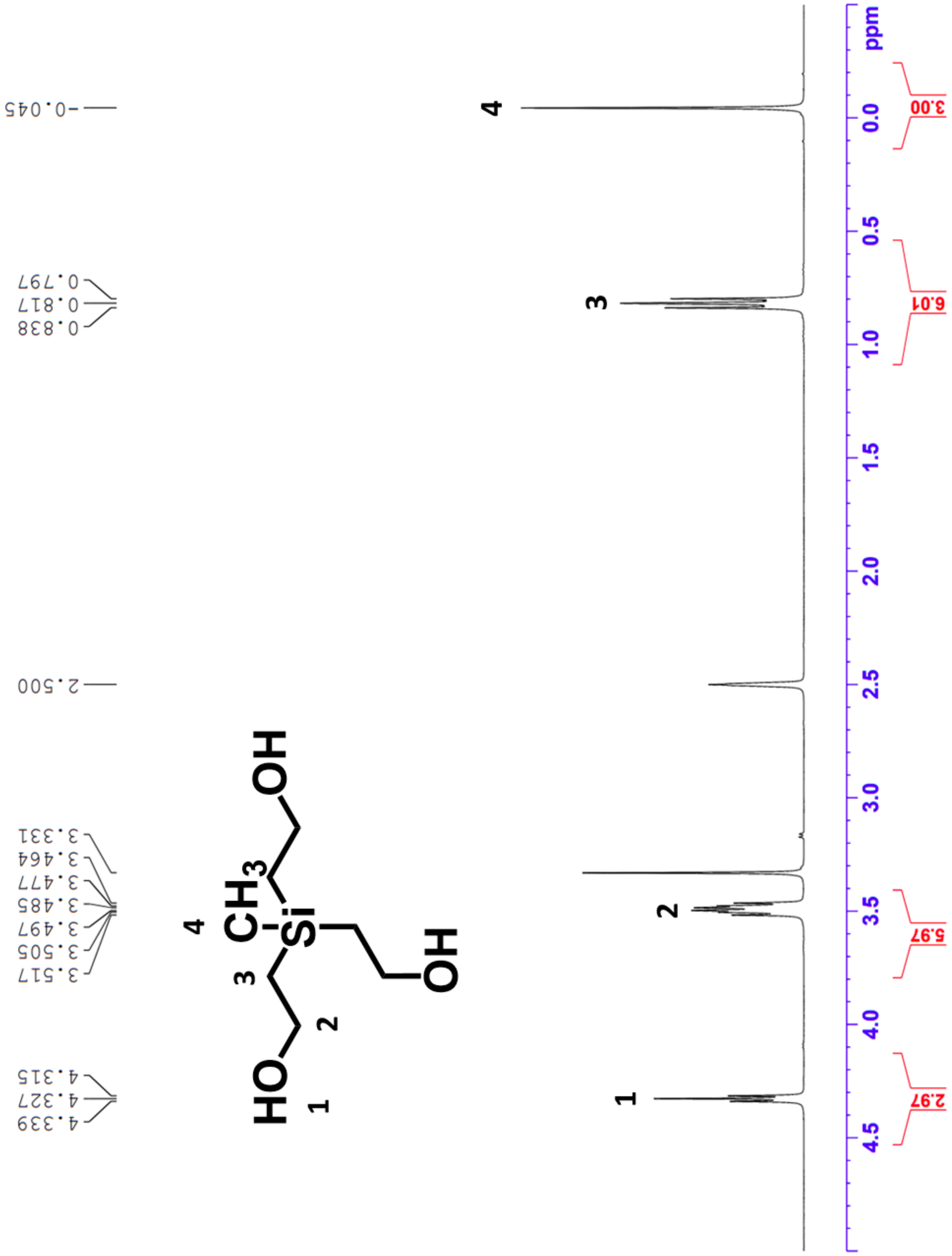


**Figure S3.** $^{1}$H NMR spectrum of 2,2′,2″-(Methylsilanetriyl)tris(ethan-1-ol) (**T2**).

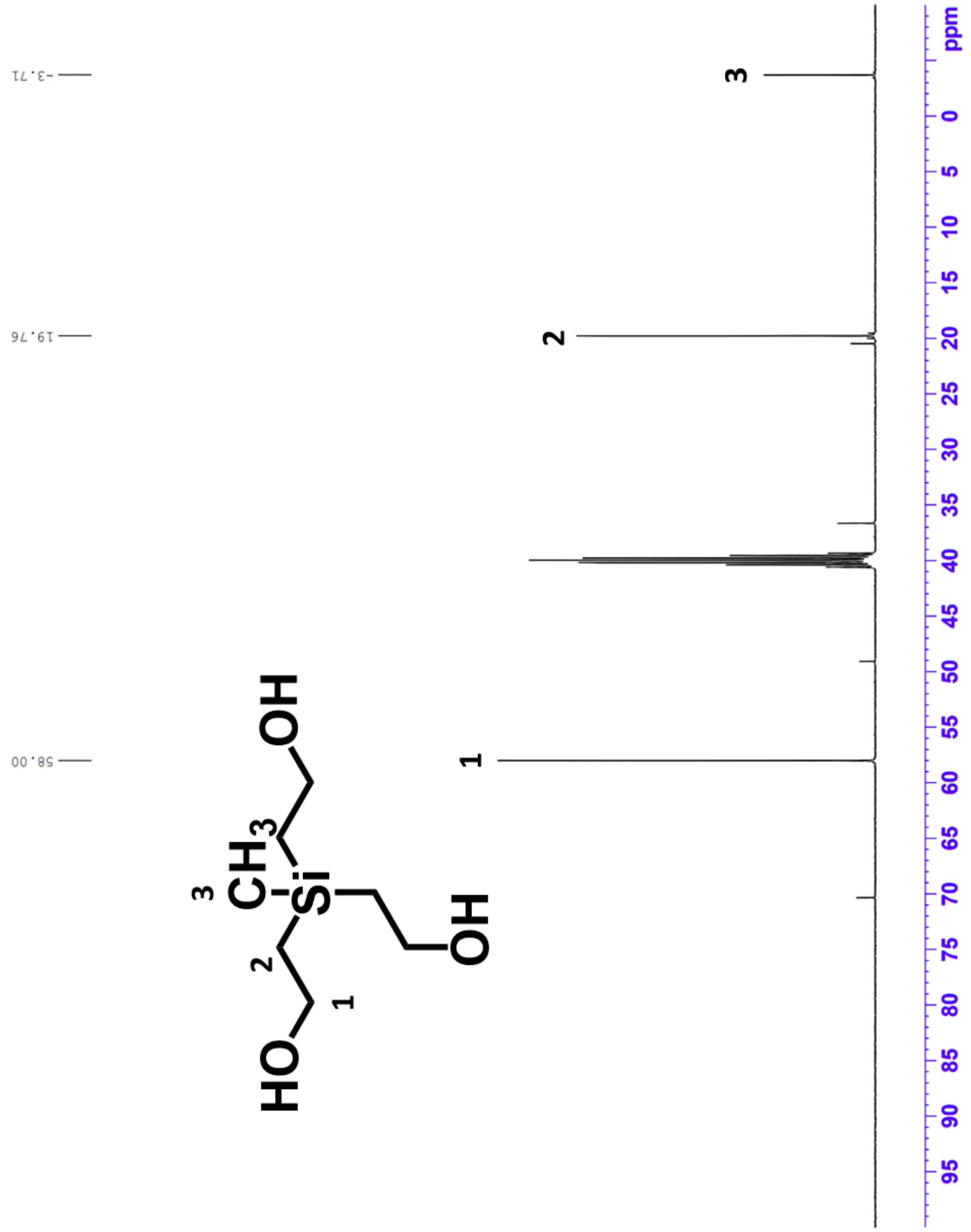


**Figure S4.** $^{13}$C NMR spectrum of 2,2′,2′′-(Methylsilanetriyl)tris(ethan-1-ol) (**T2**).

**2,2′,2′′-(Phenylsilanetriyl)tris(ethan-1-ol) (T3)**:

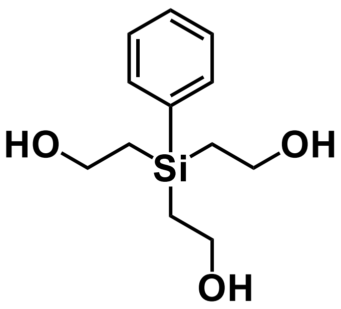


A solution of 0.5 M 9-borabicyclo[3.3.1]nonane in THF (300.6 mL, 150.3 mmol) was added to a 1 L three-neck round bottom flask and cooled to 0 °C using an ice bath. Phenyltrivinylsilane (7.5 g, 40.1 mmol) was added drop-wise to the flask over a period of 15 minutes after which the ice bath was removed and the reaction mixture was stirred for 4 hours. After 4 hours, water (50 mL) was added to the flask, followed by a 3.0 M aqueous sodium hydroxide solution (50 mL). Subsequently, aqueous hydrogen peroxide solution (30 Wt.%, 50 mL) was added dropwise at 0 °C for over a 1 hour period. The reaction mixture was heated to reflux and stirred for 3 hours. Upon cooling to room temperature, the reaction mixture was extracted with ethyl acetate (150 mL). The organic layer was dried using magnesium sulfate and concentrated *in vacuo* to afford a clear oil. Purification by column chromatography on silica gel (9:1 $CH_2Cl_2$:$CH_3OH$) furnished **T3** as a colorless oil (7.6 g, 76.0% yield). $^1$H NMR (400 MHz, DMSO-$d_6$, 25 °C): $\delta$ = 7.52 (m, 2H, H5), 7.36 (m, 3H, H4 and H6), 4.49 (s, 3H, H1), 3.54 (t, $J$ = 8.2 Hz, 2H, H2), 1.14(t, $J$ = 8.0 Hz, 3H, H3). $^{13}$C NMR (100 MHz, DMSO-$d_6$, 25 °C): $\delta$ = 137.08 (C3), 134.26 (C5), 129.40(C4), 128.24 (C6), 57.87 (C1), 18.44 (C2). HRMS (DSA) *m/z*: [M+H] calcd for $C_{12}H_{20}O_3Si$: 241.1254; found 241.1265. The $^1$H and $^{13}$C NMR spectra are shown in Figures S5 and S6.

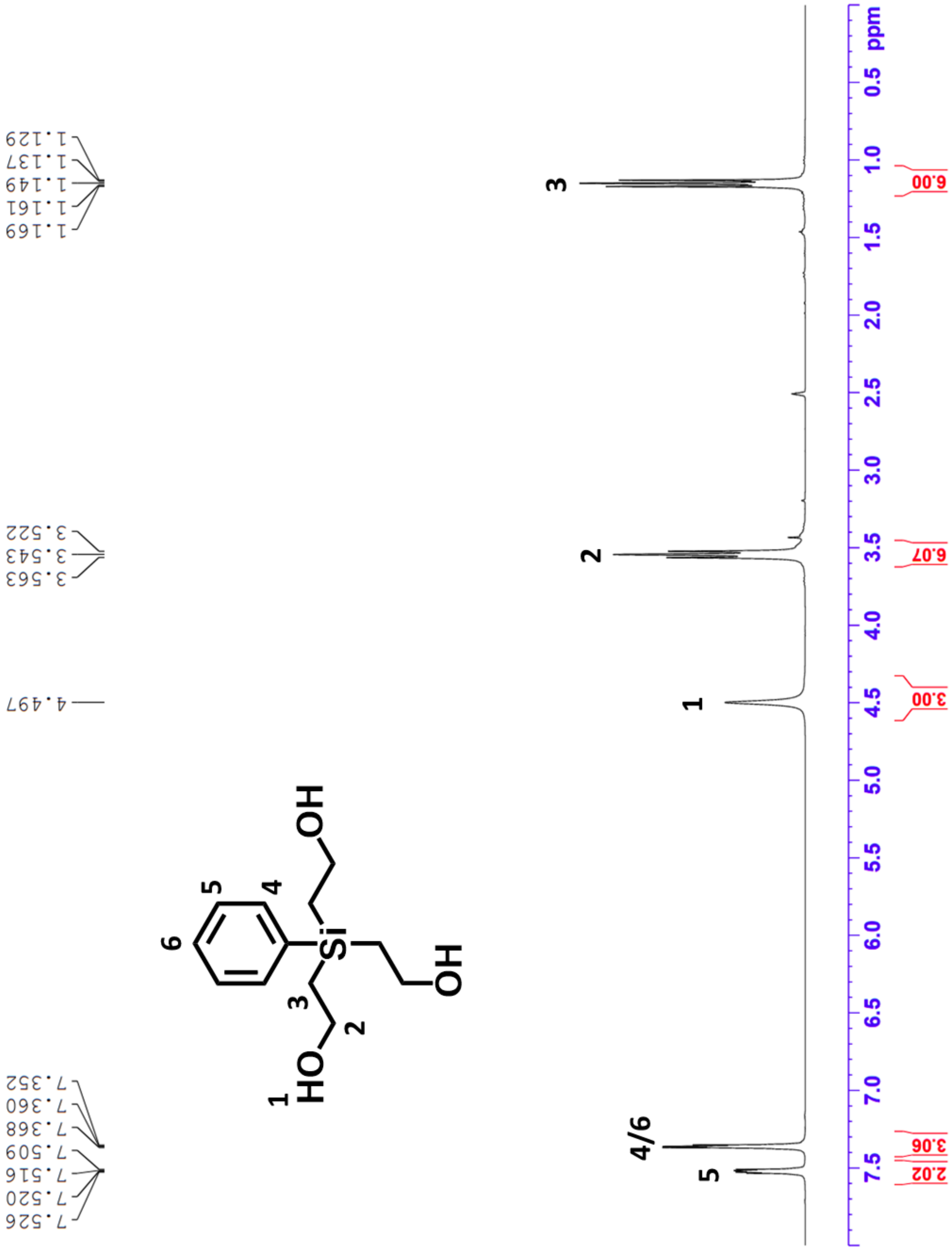


**Figure S5.** $^1$H NMR spectrum of 2,2′,2″-(Phenylsilanetriyl)tris(ethan-1-ol) (**T3**).

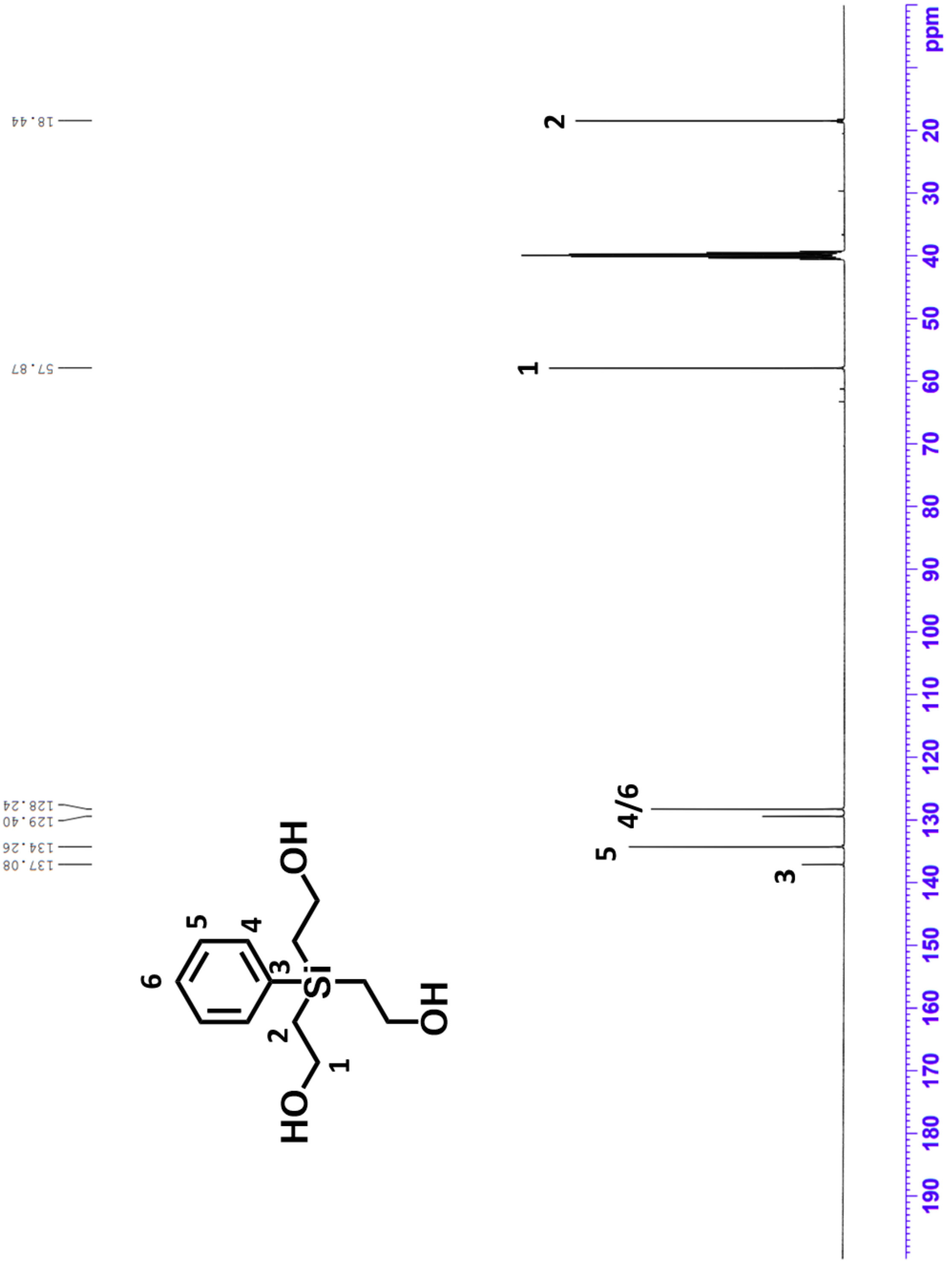


**Figure S6.** $^{13}$C NMR spectrum of 2,2′,2′′-(Phenylsilanetriyl)tris(ethan-1-ol) (**T3**).

**(Methylsilanetriyl)tris(ethane-2,1-diyl) tris((2-hydroxyethyl)(methyl)carbamate) (T4)**:

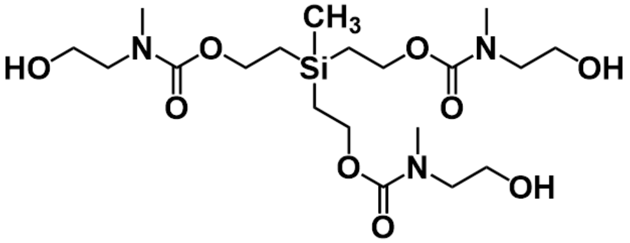

2,2′,2′′-(Methylsilanetriyl)tris(ethan-1-ol) (**T2**) (4.5 g, 25.0 mmol) and triethylamine (27.8 mL, 199.3 mmol) were added to a 500 mL round bottom flask containing dry acetonitrile (250 mL). *N,N'*-disuccinimidyl carbonate (25.5 g, 99.7 mmol) was then added to the flask with a stir bar and allowed to stir for 16 hours at room temperature. The reaction mixture was concentrated *in vacuo* to afford a yellow oil. The oil was dissolved in ethyl acetate (300 mL) and washed with a saturated aqueous NaCl solution (2 x 100 mL). The organic layer was concentrated *in vacuo* to afford a yellow oil. The oil was dissolved in methylene chloride (150 mL). Triethylamine (24.4 mL, 175.0 mmol) was added to the flask, followed by *N*-methylethanolamine (7.0 mL, 87.5 mmol). The reaction mixture was stirred at room temperature for 16 hours. The reaction mixture was concentrated *in vacuo* to afford a yellow oil. Purification by column chromatography (9:1 $CH_2Cl_2$:$CH_3OH$) afforded **T4** as a clear, colorless oil (8.8 grams, 72.6% yield). $^1H$ NMR (400 MHz, DMSO-$d_6$, 25 °C): $\delta$ = 4.66 (bs, 3H, H1), 4.07(t, $J$ = 7.6 Hz, 6H, H5), 3.47 (q, $J$ = 11.2, 5.6 Hz, 6H, H2), 3.23 (t, $J$ = 6 Hz, 6H, H3), 2.85 (s, 9H, H4), 1.020 (t, $J$ = 7.6 Hz, 6H, H6), 0.06 (s, 3H, H7). $^{13}C$ NMR (100 MHz, DMSO-$d_6$, 25 °C): $\delta$ = 156.15 (C4), 62.51 (C5), 59.47 (C1), 51.29 (C2), 35.63 (C3), 15.32 (C6), -4.28 (C7). HRMS (DSA) *m/z*: [M+H] calcd for $C_{19}H_{39}N_3O_9Si$: 482.2528; found 482.2518. The $^1H$ and $^{13}C$ NMR spectra are shown in Figures S7 and S8.

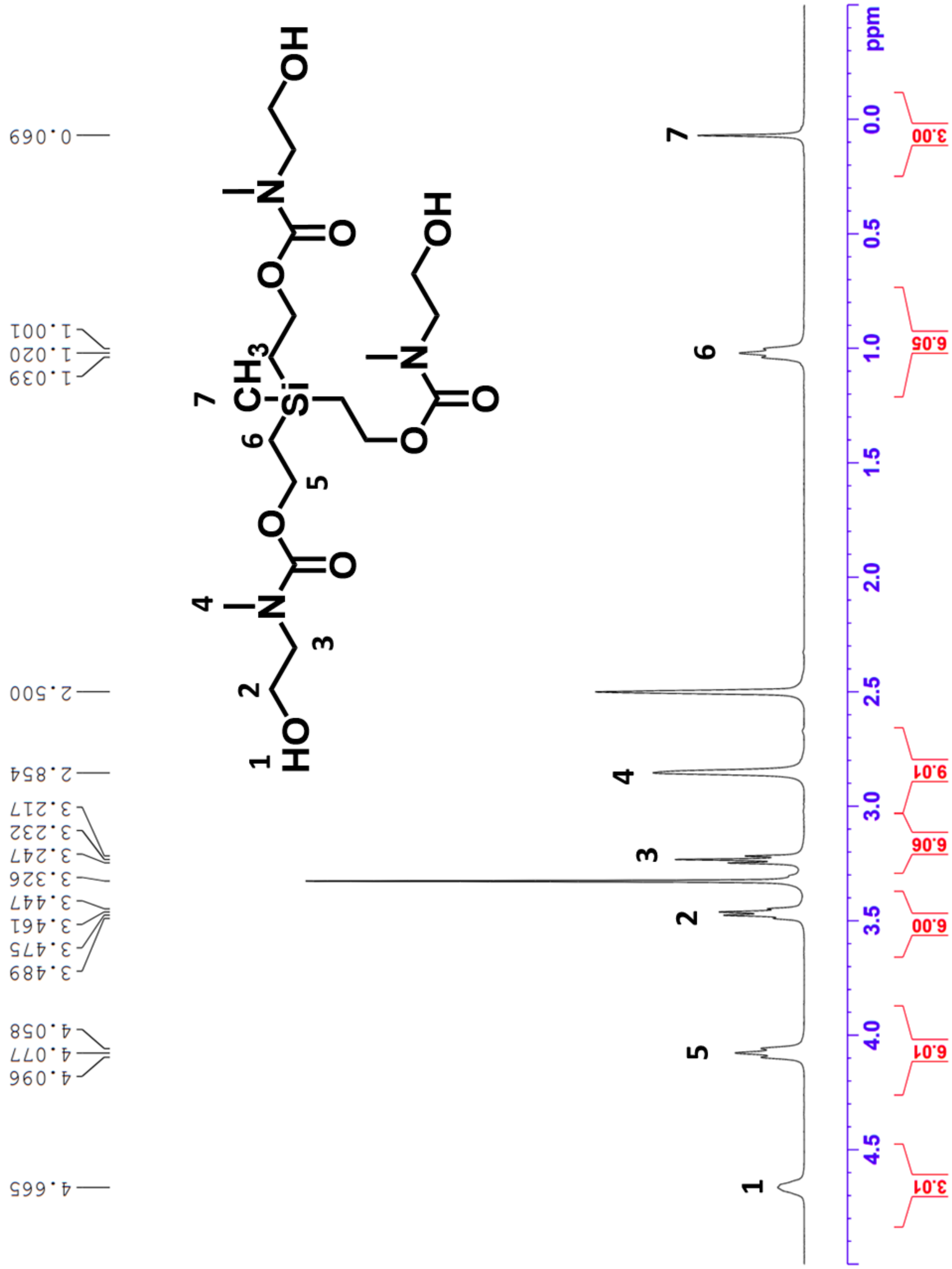


**Figure S7.** $^{1}$H NMR spectrum of (Methylsilanetriyl)tris(ethane-2,1-diyl) tris((2-hydroxyethyl)(methyl)carbamate) (**T4**).

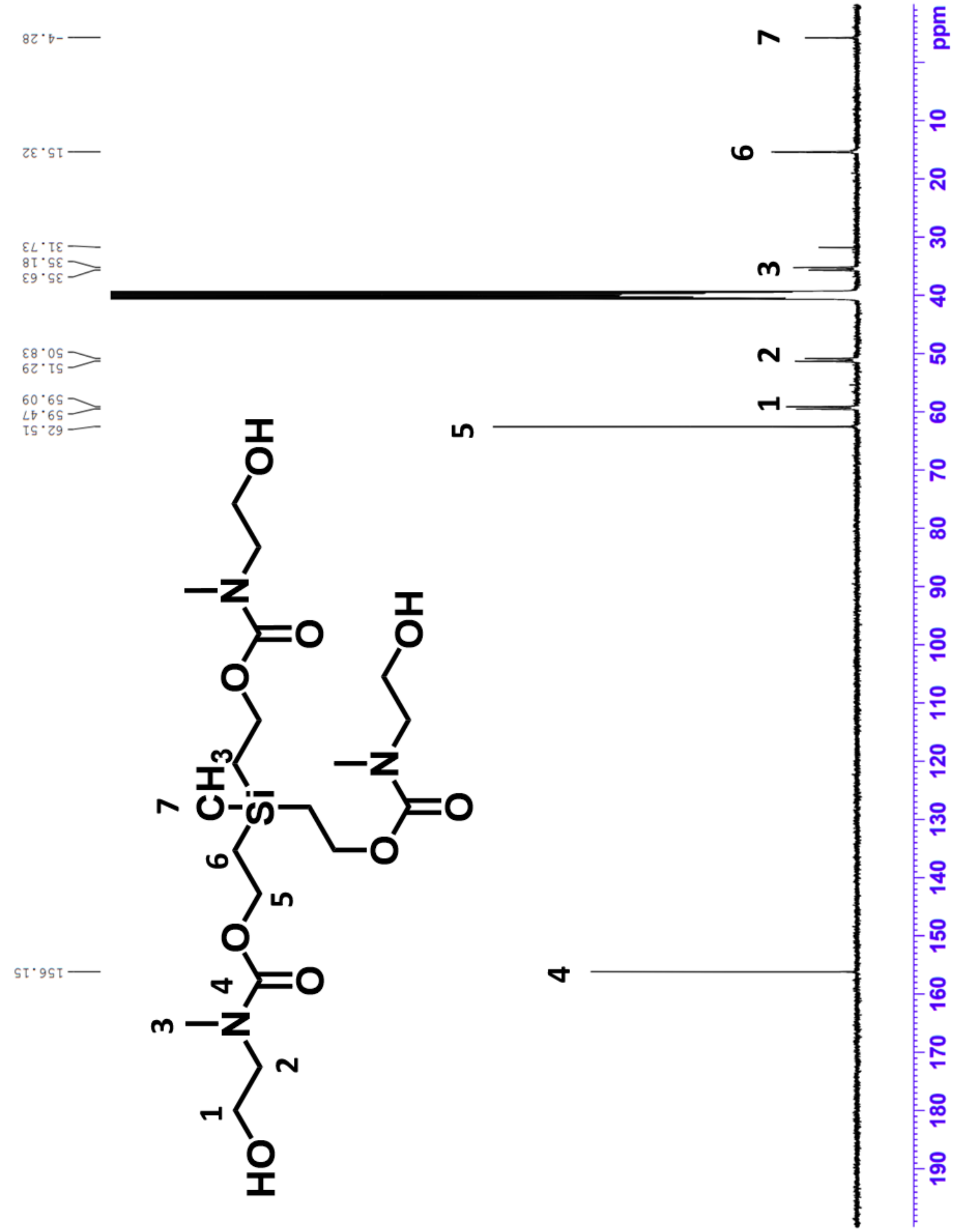


**Figure S8.** $^{13}C$ NMR spectrum of (Methylsilanetriyl)tris(ethane-2,1-diyl) tris((2-hydroxyethyl)(methyl)carbamate) (**T4**).

**(Phenylsilanetriyl)tris(ethane-2,1-diyl) tris((2-hydroxyethyl)(methyl)carbamate) (T5):**

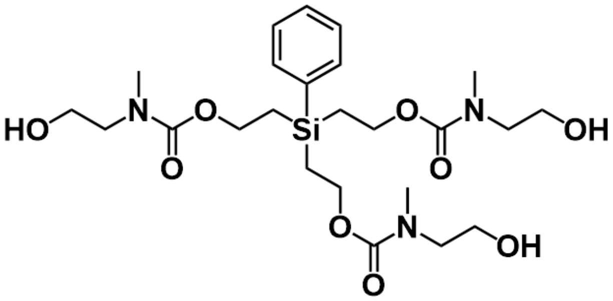


2,2′,2′′-(Phenylsilanetriyl)tris(ethan-1-ol) (**T4**) (9.98 g, 42.0 mmol) and triethylamine (34.0 mL, 243.9 mmol) were added to a 500 mL round bottom flask containing dry acetonitrile (200 mL). *N,N'*-disuccinimidyl carbonate (42.5 g, 165.9 mmol) was then added to the flask with a stir bar and allowed to stir for 16 hours at room temperature. The reaction mixture was concentrated *in vacuo* to afford a yellow powder. The powder was dissolved in chloroform (200 mL) and washed with a saturated aqueous NaCl solution (3 x 50 mL). The organic layer was concentrated *in vacuo* to afford a yellow powder. The powder was dissolved in acetonitrile (200 mL). Triethylamine (29.3 mL, 210.0 mmol) was added to the flask, followed by *N*-methylethanolamine (13.5 mL, 168.0 mmol). The reaction mixture was stirred at room temperature for 16 hours. The mixture was concentrated *in vacuo* to afford a yellow oil. Purification by column chromatography (9:1 $CH_2Cl_2$:$CH_3OH$) afforded T5 as a clear, colorless oil (8.8 grams, 72.6% yield). $^1$H NMR (400 MHz, DMSO, 25 °C): δ = 7.56 (m, 2H, H8), 7.41-7.39 (m, 3H, H7 and H9), 4.65 (m, 3H, H1), 4.08 (t, J= 7.8 Hz, 6H, H5), 3.44 (q, J= 12.4, 6 Hz, 6H, H2), 3.19 (m, 6H, H3), 2.82 (s, 9H, H4), 1.33 (t, J= 7.4 Hz, 6H, H6). $^{13}$C NMR (100 MHz, DMSO, 25 °C): δ = 156.06 (C4), 134.22 (C8), 129.99 (C7), 128.50 (C9), 128.40 (C10), 62.32 (C5), 59.49 (C1), 51.30 (C2), 53.62 (C3), 14.09 (C6). HRMS (DSA) *m/z*: [M+H] calcd for $C_{24}H_{41}N_3O_9Si$: 544.2684; found 544.2697. The $^1$H and $^{13}$C NMR spectra are shown in Figure S9 and S10.

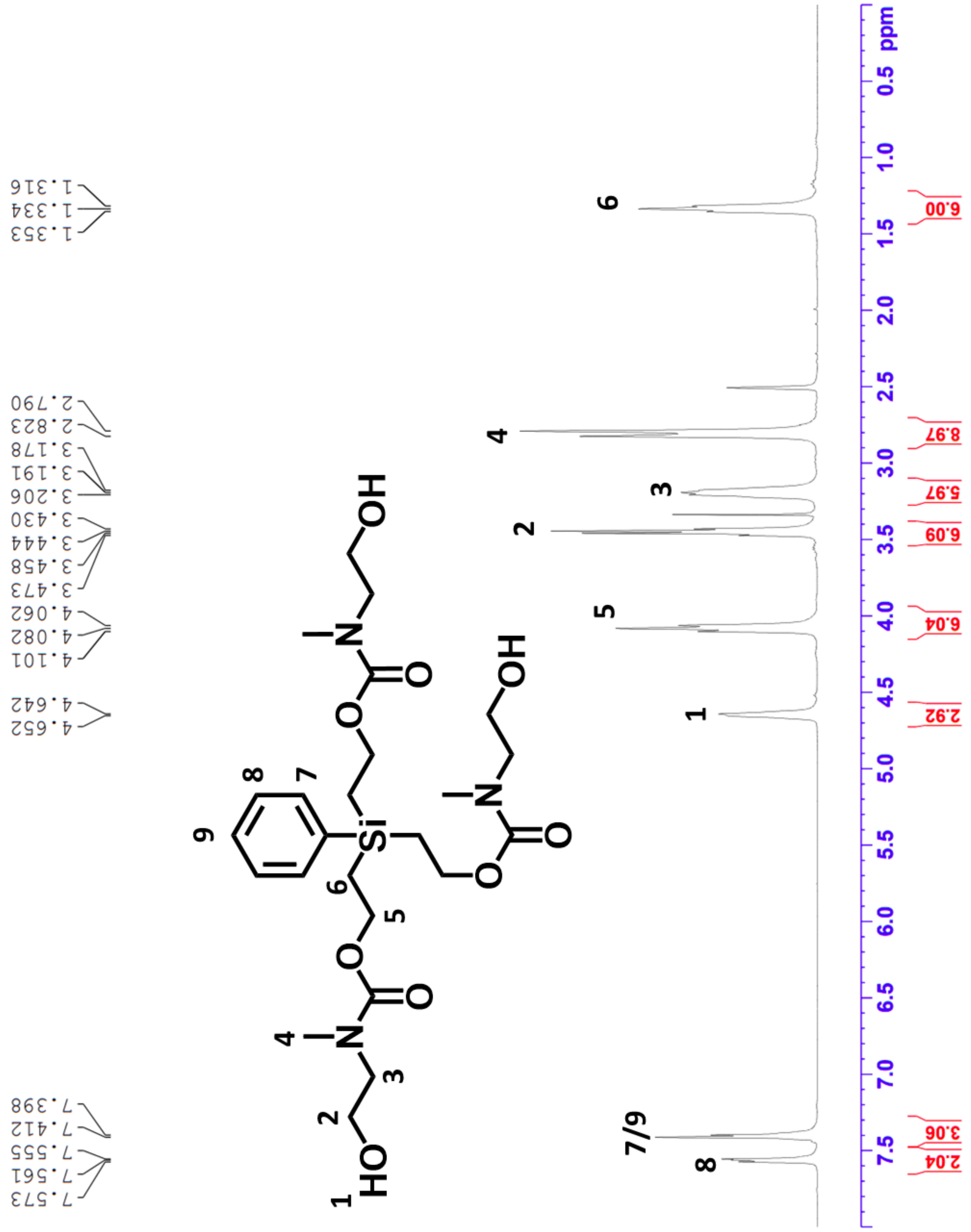


**Figure S9.** $^{1}$H NMR spectrum of (Phenylsilanetriyl)tris(ethane-2,1-diyl) tris((2-hydroxyethyl)(methyl)carbamate) (**T5**).

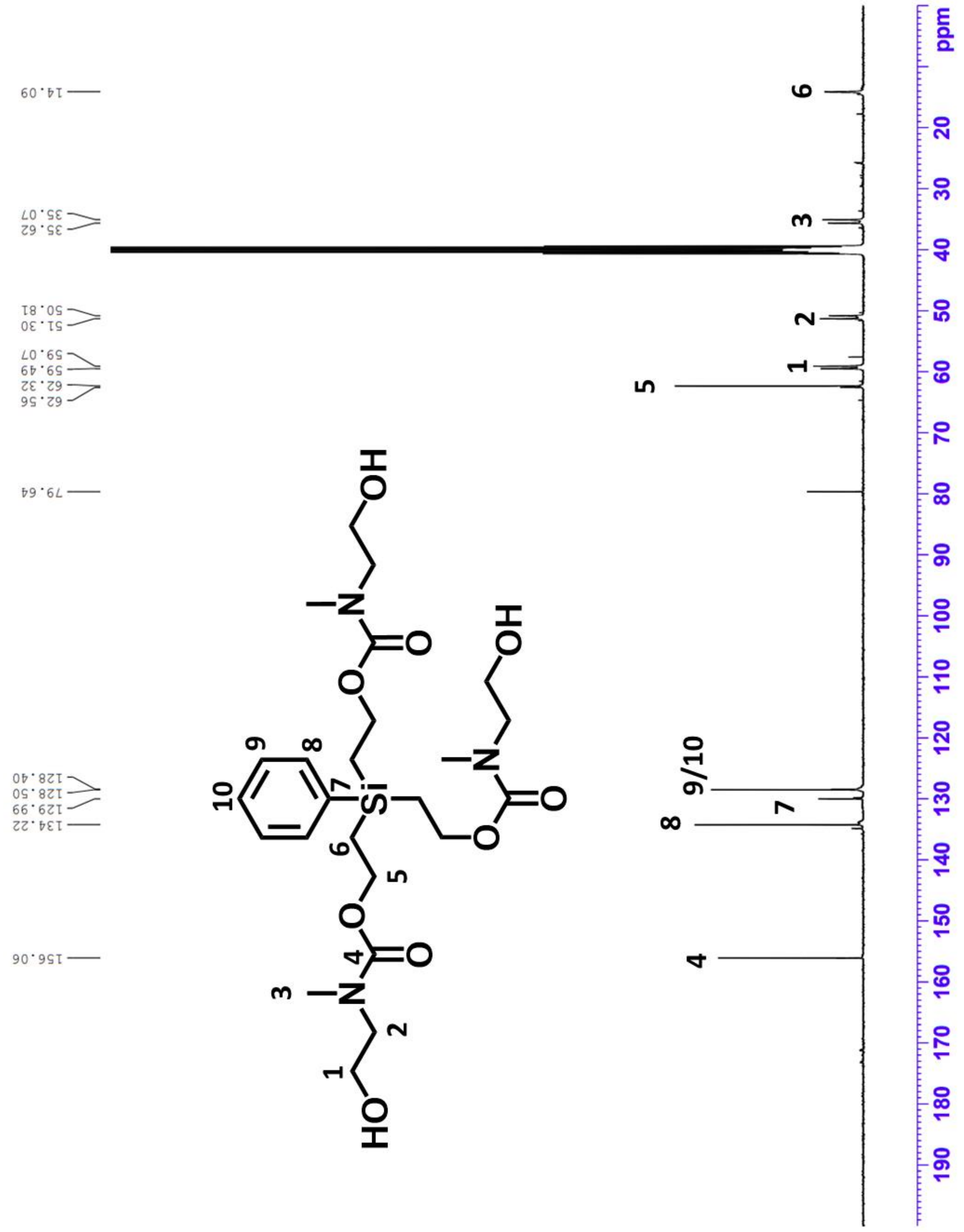


**Figure S10.** $^{13}C$ NMR spectrum of (Phenylsilanetriyl)tris(ethane-2,1-diyl) tris((2-hydroxyethyl)(methyl)carbamate) (**T5**).

**1-Methyl-tris(2-hydroxyethoxy)silane (T6)**:

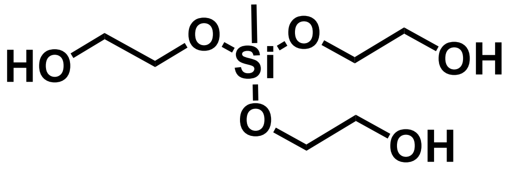


Trimethoxymethylsilane (MTMOS) (20.00 g, 146.8 mmol) and ethylene glycol (26.88 g, 433.10 mmol) were added to a round-bottom flask with a stir bar. MTMOS and ethylene glycol were not completely miscible and two distinct layers were present. A short path distillation head was attached to the RBF with a collection flask. The mixture was heated to 95°C under $N_2$ and stirred for 24 h. Methanol began to distill into the collection flask. After 24 h, the reaction was cooled to room temperature and any residual methanol was removed under reduced pressure. The product (**T6**) was isolated as a clear liquid (28.00 grams, 84.3% yield) $^{1}$H NMR (400 MHz, $CDCl_3$, 25 °C): $\delta$ = 3.89-3.87 (6H, SiOC*H*$_2$), 3.68-3.65 (6H, C*H*$_2$OH), 0.21 (3H, SiC*H*$_3$); $^{13}$C NMR (100 MHz, $CDCl_3$, 25 °C): $\delta$ = 64.65, 63.45, -7.70. Spectroscopic data agrees with reported data in the literature.[3]

**3. Synthesis of Poly(urethane) Networks with Excess Hydroxyl Functionality (N2-N4)**

Silyl-containing poly(urethane) **N2** was synthesized by adding 2,2′,2′′-(methylsilanetriyl)tris(ethan-1-ol) (2.00 grams, 0.0336 moles OH) (**T2**) to a 25 ml round bottom flask, followed by the addition of dry ethyl acetate (2.42 grams). Next, 1,6-hexamethylene diisocyanate (2.69 grams, 0.032 moles NCO) was added, followed by stirring and heating at 60 °C for 1 hour until the mixture became clear. The solution was poured into a 2.5-inch aluminum weighing pan, the solvent was allowed to evaporate for 30-60 minutes, then the pan was placed in the oven at 60 °C for 36 hours to form the solid network. Network thickness was 1-2 mm.

Silyl-containing poly(urethane) **N3** was synthesized by adding 2,2′,2′′-(phenylsilanetriyl)tris(ethan-1-ol) (2.00 grams, 0.0249 moles OH) (**T3**) to a 25 ml round bottom flask, followed by the addition of dry ethyl acetate (2.00 grams). Next, 1,6-hexamethylene diisocyanate (2.00 grams, 0.0238 moles NCO) was added, followed by stirring and heating at 60 °C for 1 hour until the mixture became clear. The solution was poured into a 2.5-inch aluminum weighing pan, the solvent was allowed to evaporate for 30-60 minutes, then the pan was placed in the oven at 60 °C for 36 hours to form the solid network. Network thickness was 1-2 mm.

Silyl-containing poly(urethane) **N4** was synthesized by adding (methylsilanetriyl)tris(ethane-2,1-diyl) tris((2-hydroxyethyl)(methyl)carbamate) (3.00 grams, 0.0187 moles OH) (**T4**) to a 25 ml round bottom flask, followed by the addition of dry ethyl acetate (2.32 grams). Next, 1,6-hexamethylene diisocyanate (1.50 grams, 0.0178 moles NCO) was added, followed by stirring and heating the solution at 60 °C for 1 hour. The solution was poured into a 2.5-inch aluminum weighing pan, the solvent was allowed to evaporate for 30-60 minutes, then the pan was placed in the oven at 60 °C for 36 hours to form the solid network. Network thickness was 1-2 mm.

## 4. Synthesis of Poly(urethane) Networks with Excess Isocyanate Functionality (N6-N9)

Silyl-containing poly(urethane) **N6** was synthesized by adding 2, 2′,2″-(methylsilanetriyl)tris(ethan-1-ol) (2.00 grams, 0.0336 moles OH) (**T2**) to a 25 ml round bottom flask, followed by the addition of dry ethyl acetate (2.69 grams). Next, 1,6-hexamethylene diisocyanate (3.23 grams, 0.0384 moles NCO) was added, followed by stirring and heating at 60 °C for 1 hour until the mixture became clear. The solution was poured into a 2.5-inch aluminum weighing pan, the solvent was allowed to evaporate for 30-60 minutes, then the pan was placed in the oven at 60 °C for 36 hours to form the solid network. Network thickness was 1-2 mm.

Silyl-containing poly(urethane) **N7** was synthesized by adding 2,2′,2″-(phenylsilanetriyl)tris(ethan-1-ol) (2.00 grams, 0.0249 moles OH) (**T3**) to a 25 ml round bottom flask, followed by the addition of dry ethyl acetate (2.16 grams). Next, 1,6-hexamethylene diisocyanate (2.19 grams, 0.0261 moles NCO) was added, followed by stirring and heating at 60 °C for 1 hour until the mixture became clear. The solution was poured into a 2.5-inch aluminum weighing pan, the solvent was allowed to evaporate for 30-60 minutes, then the pan was placed in the oven at 60 °C for 36 hours to form the solid network. Network thickness was 1-2 mm.

Silyl-containing poly(urethane) **N8** was synthesized by adding (methylsilanetriyl)tris(ethane-2,1-diyl) tris((2-hydroxyethyl)(methyl)carbamate) (2.97 grams, 0.0185 moles OH) (**T4**) to a 25 ml round bottom flask, followed by the addition dry ethyl acetate (2.37 grams). Next, 1,6-hexamethylene diisocyanate (1.63 grams, 0.0194 moles NCO) was added, followed by stirring and heating the solution at 60 °C for 1 hour. The solution was poured into a 2.5-inch aluminum weighing pan, the solvent was allowed to evaporate for 30-60 minutes, then the pan was placed in the oven at 60 °C for 36 hours to form the solid network. Network thickness was 1-2 mm.

Silyl-containing poly(urethane) **N9** was synthesized by adding (phenylsilanetriyl)tris(ethane-2,1-diyl) tris((2-hydroxyethyl)(methyl)carbamate) (2.69 grams, 0.0148 moles OH) (**T5**) to a 25 ml round bottom flask, followed by the addition of dry ethyl acetate (2.06 grams). Next, 1,6-hexamethylene diisocyanate (1.31 grams, 0.0156 moles NCO) was added, followed by stirring and heating the solution at 60 °C for 1 hour. The solution was poured into a 2.5-inch aluminum weighing pan, the solvent was allowed to evaporate for 30-60 minutes, then the pan was placed in the oven at 60 °C for 36 hours to form the solid network. Network thickness was 1-2 mm.

## 5. Synthesis of Silyl Ether Containing Poly(urethane) Network with Excess Hydroxyl Functionality (N10)

Silyl ether poly(urethane) (**N10**) was synthesized by adding 1-methyl-tris(2-hydroxyethoxy)silane (12.00 grams, 0.159 moles OH) (**T6**) to a 40 ml plastic cup, followed by the addition of dry ethyl acetate (2.74 grams). Next, 1,6-hexamethylene diisocyanate (HDI) (12.8 grams, 0.152 moles NCO) was added, followed by 10 Wt.% solution of DBTDL in dry ethyl acetate (0.10 grams). The solution was then stirred by hand for 10-15 minutes until the solution became warm. The solution was poured into a 2.5-inch aluminum weighing pan and allowed to cure under ambient conditions for 24 hours. The thickness of the silyl ether poly(urethane) network was 2-3 mm (Figure S26).

## 6. Synthesis of Epoxy-Amine Network

Epoxy-amine networks were made by adding liquid Bisphenol A / epichlorohydrin difunctional epoxy resin (Epon 828, Miller-Stevenson) (39.73 grams, 0.0421 grams per epoxy equivalence) to a plastic cup, followed by addition of xylenes (2.50 or 5.50 grams) and tris(2-aminoethyl)amine (10.27 grams, 0.0422 grams per NH equivalent). The mixtures were stirred with a magnetic stir bar for 20 minutes, followed by spin-coating the dilute solution on the gold slides and applying the concentrated solution on the chromic acid anodized aluminum alloy panels via a 3 mil (76.2 micron) film forming bar. The gold slides were placed in the oven at 60 °C for 24 hours, whereas the aluminum panels were allowed to cure under normal laboratory conditions for 24 hours. The film thickness of the cured epoxy-amine network on the gold slides was 10-15 microns, whereas the cured thickness on the pretreated aluminum panels was an average of 40 microns.

## 7. Synthesis of Poly(urethane) Networks (N1-N5) over Epoxy-Coated Gold Slides

Non-silyl and silyl-containing poly(urethane) networks **N1-N5** over epoxy-coated gold slides were formed by remaking the network formulas according to the aforementioned procedures, but instead of decanting into an aluminum pan they were spin-coated on the 24-hour cured epoxy-coated gold slides. The samples were then placed in the oven at 60 °C for 24-hours. The resulting film thickness of the poly(urethane) networks was about 10-15 microns.

## 8. Tables S1-S5 and Figures S11-S33

**Table S1**. Sample label and description, gel fraction, and onset degradation temperature of PU networks **N6-N9**.

| Thermoset Network | Central Group | OH:NCO Ratio | Chain Extension | Gel Fraction | Onset Degradation Temperature (°C) |
|---|---|---|---|---|---|
| N6 | Methyl-Si | 1.00:1.05 | N | 0.98 | 303.2 |
| N7 | Phenyl-Si | 1.00:1.05 | N | 0.99 | 301.8 |
| N8 | Methyl-Si | 1.00:1.05 | Y | 0.96 | 294.0 |
| N9 | Phenyl-Si | 1.00:1.05 | Y | 0.97 | 280.0 |

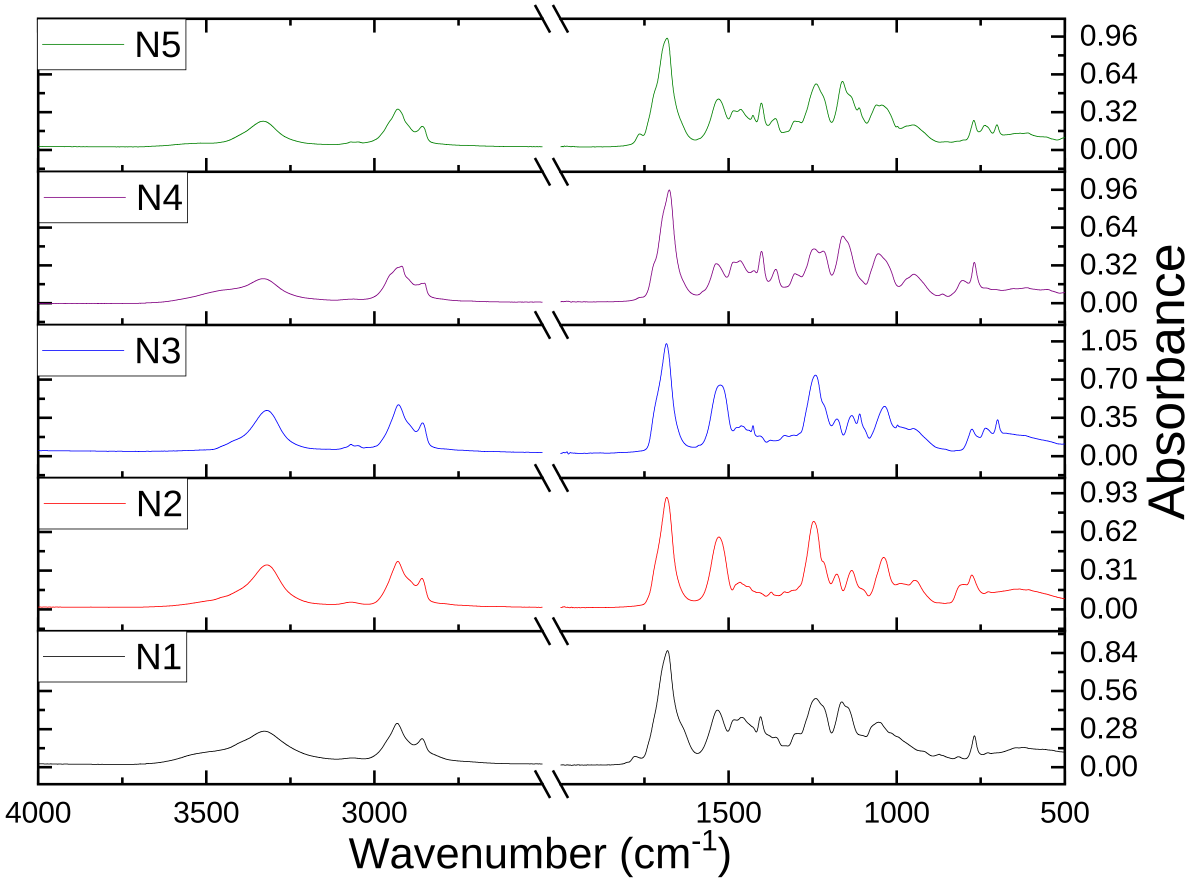


**Figure S11.** ATR-IR spectra for networks **N1-N5**.

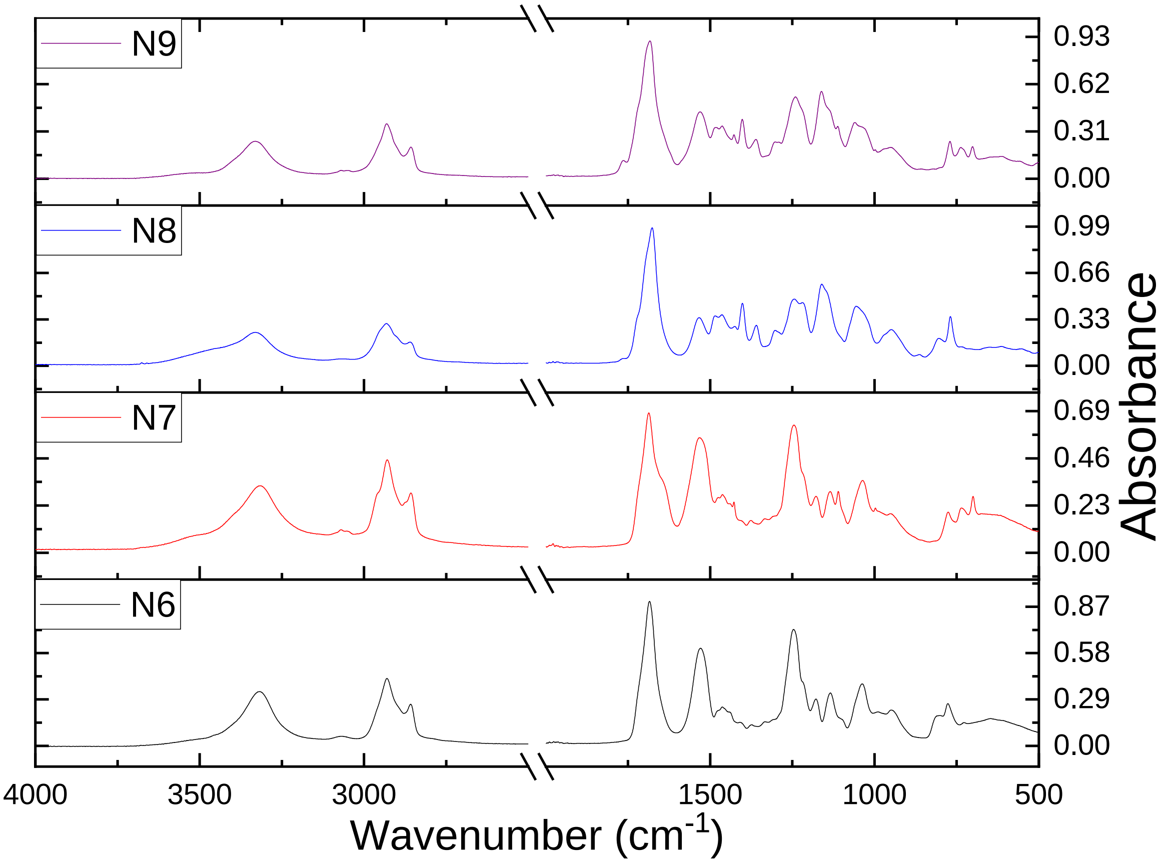


**Figure S12.** ATR-IR spectra of networks **N6-N9**.

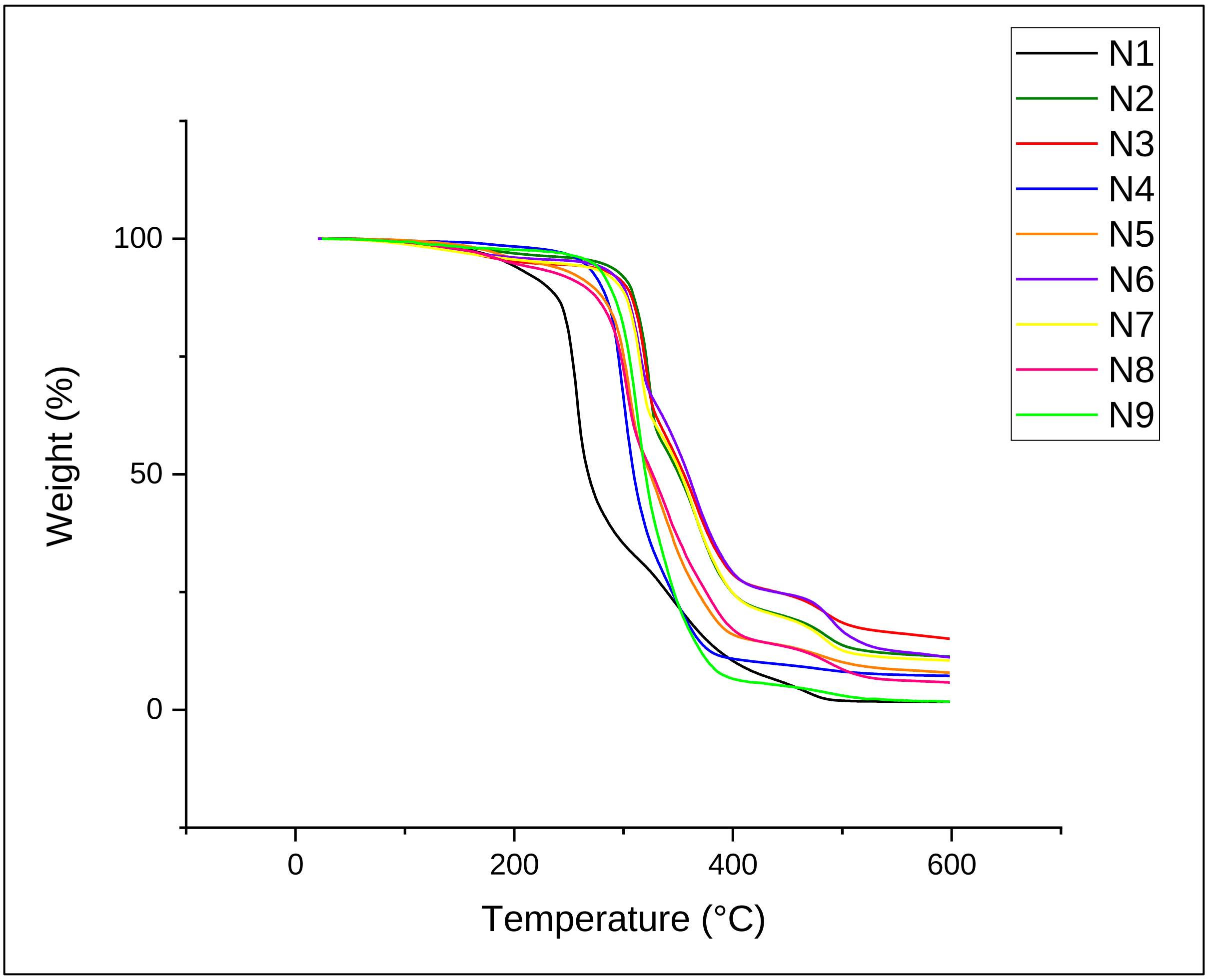


**Figure S13.** TGA degradation profiles of PU networks **N1-N9**.

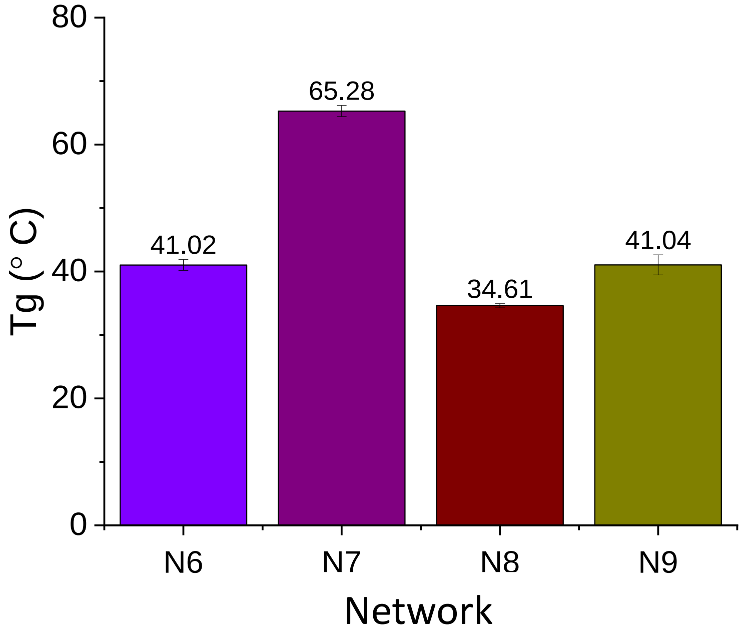


**Figure S14.** Glass transition temperature of Silyl-PU networks **N6-N9**.

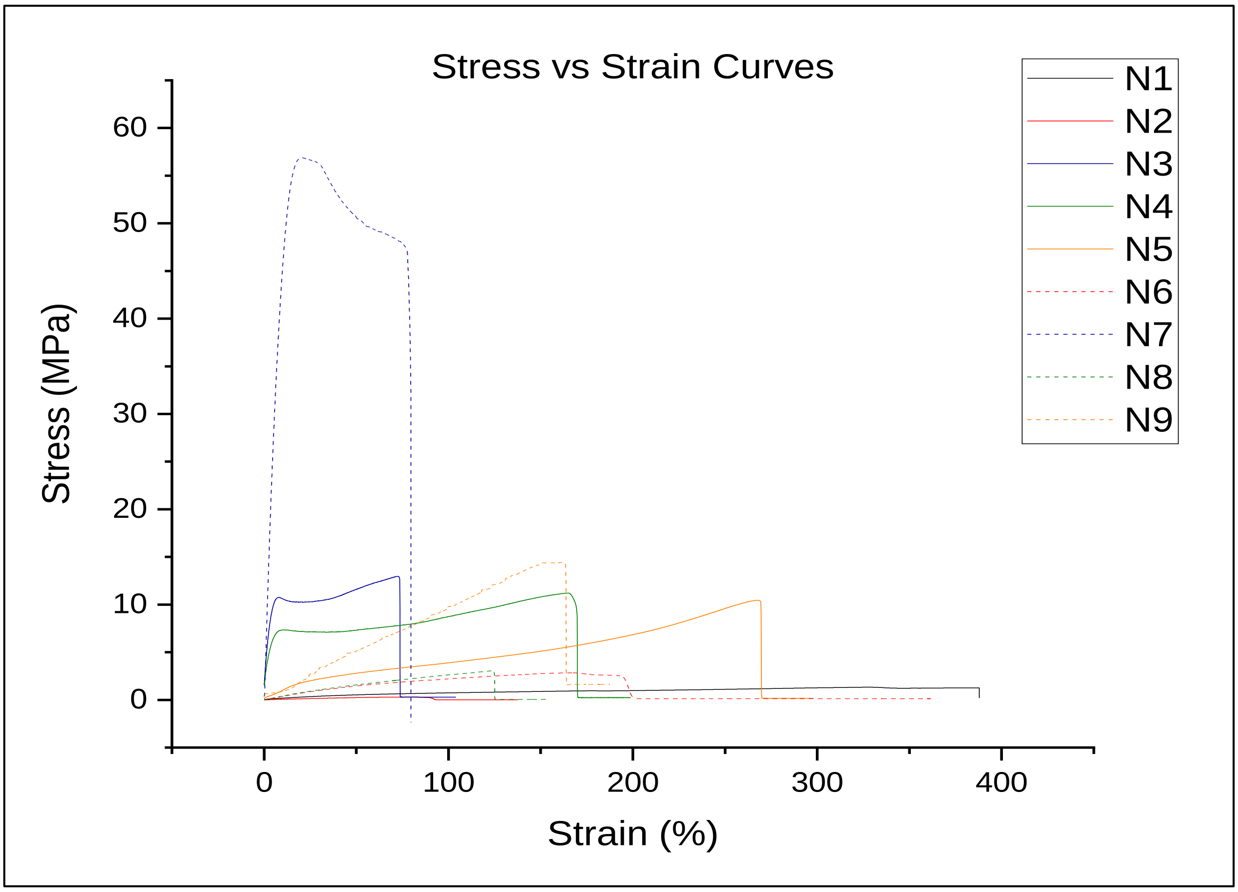


**Figure S15.** Stress vs Strain curves of PU networks **N1-N9**.

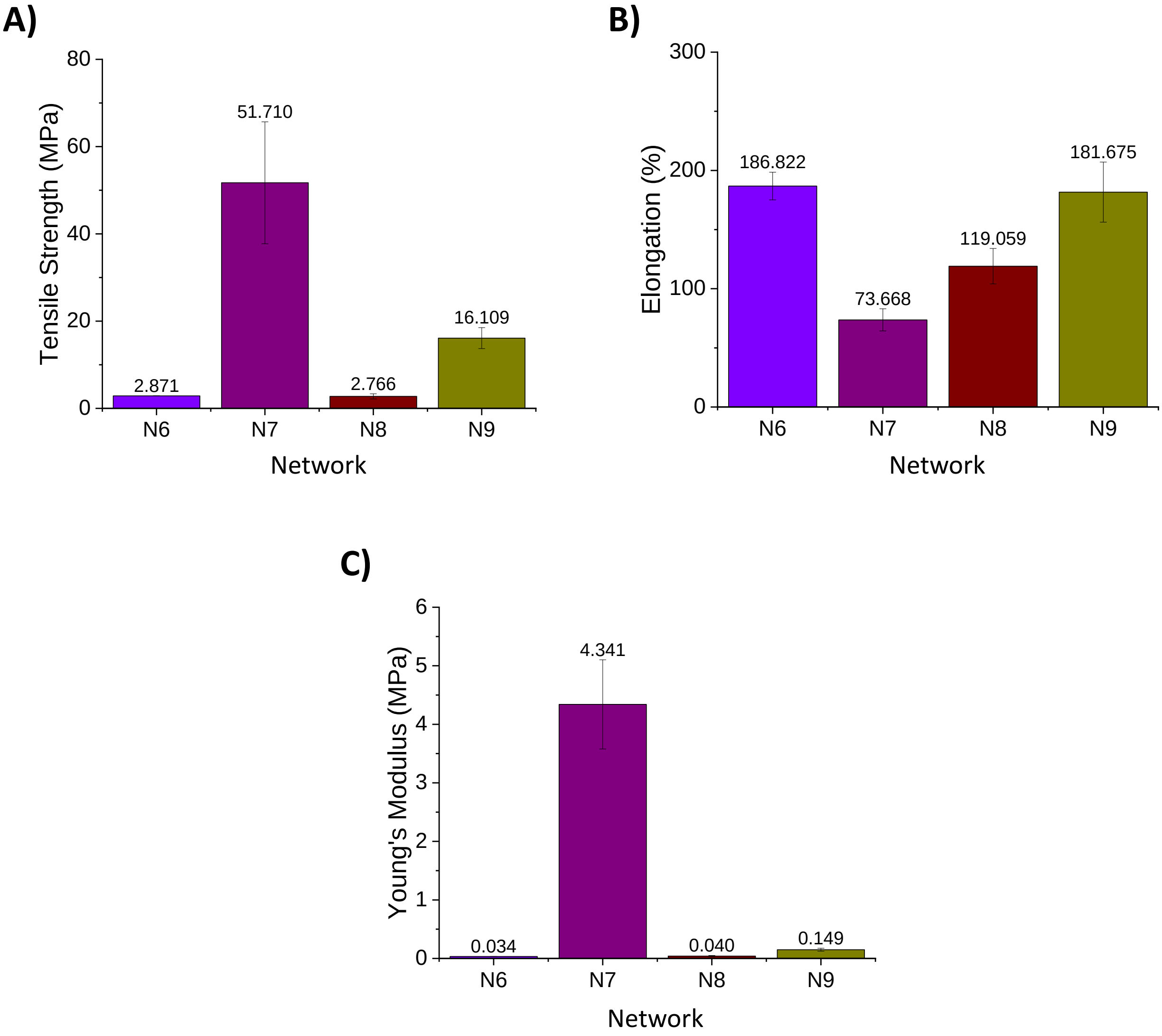


**Figure S16**. Mechanical properties of Silyl-PU networks **N6-N9**. (A) Ultimate tensile strength. (B) Percent elongation. (C) Young's Modulus.

**Table S2**. Comparison of thermal and mechanical properties for Silyl-PUs **N4**, **N5**, and **N7**, the non-silyl PU control (**N1**), and several reported traditional PU networks.

| PU Networks | $T_g$ (°C) via DSC | Onset Degradation Temp. (°C) | Tensile Strength (MPa) | Elongation (%) | Storage Modulus ($E'$) (MPa) in Glassy State |
|---|---|---|---|---|---|
| **N1** (control) | 16.5 | 244.6 | 1.24 | 257.2 | 410.8 |
| Silyl-PU **N4** | 24.7 | 284.3 | 7.55 | 256.4 | 3499 |
| Silyl-PU **N5** | 34.9 | 291.4 | 9.88 | 134.0 | 2677 |
| Silyl-PU **N7** | 65.3 | 301.8 | 51.7 | 73.7 | N/A |
| PU coatings from acrylic polyols[4] | N/A | 280.4-360.4 | 1.82-2.12 | 81-107 | N/A |
| PU from recycled component[5] | -35 to -25 | N/A | 24.6-34.8 | 293-892 | 2083-2285 |
| PU coatings from rapeseed oil/TEA polyols[6] | N/A | N/A | 10.6-39.3 | 4.3-15.1 | N/A |
| PU from PPG2000, TMP, and TDI[7] | -40.5 | N/A | 2.1 | 150 | N/A |
| PU from PTMEG1000, TMP, and IPDI[8] | N/A | N/A | 26.4 | 140 | N/A |
| PU from HTPB2600, TMP, and aromatic isocyanates[9] | -72.8 to -69.9 | 295-315 | 2.1-3.6 | 79-350 | N/A |
| PU-ureas from hyperbranched TEA esters and HMDI[10] | 75.5-89.0 | 225-236 | 15.8-37.3 | N/A | 78.7-506 |

**Table S3**. Glass transition temperature ($T_g$), as determined by DMA, for Silyl-PU thermoset networks **N1-N5** in Figure 3.

| Thermoset Network | $T_g$ (°C) via DMA |
|---|---|
| N1 | 13.5 |
| N2 | 34.9 |
| N3 | 41.7 |
| N4 | 31.0 |
| N5 | 35.7 |

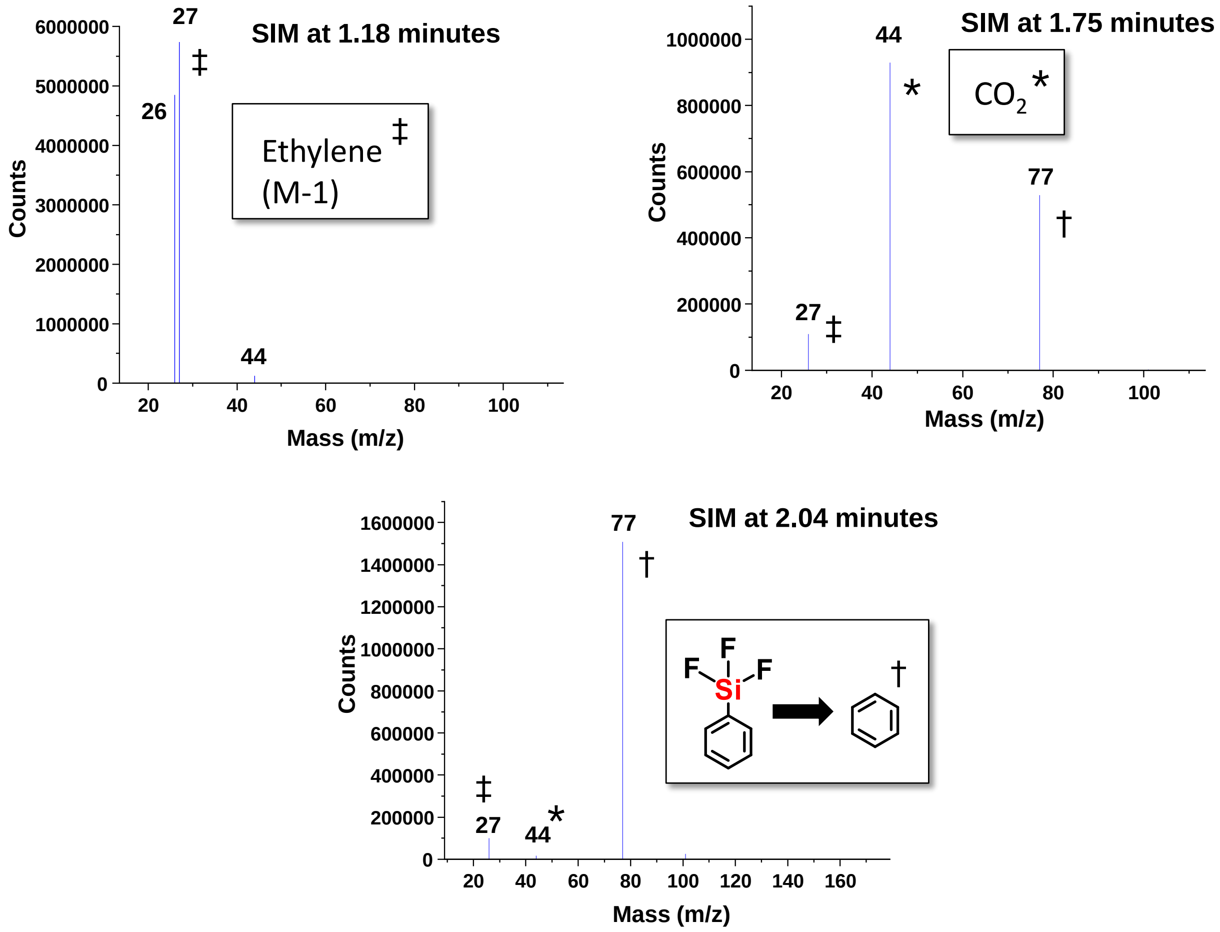


**Figure S17.** Mass spectra (from HS-GC-MS analysis) of partially degraded Silyl-PU **N5** in 1.0 M TBAF (DMF) with detected ions corresponding to ethylene, $CO_2$, and phenyl (from trifluorophenysilane). All spectra show ions corresponding to coeluted molecules.

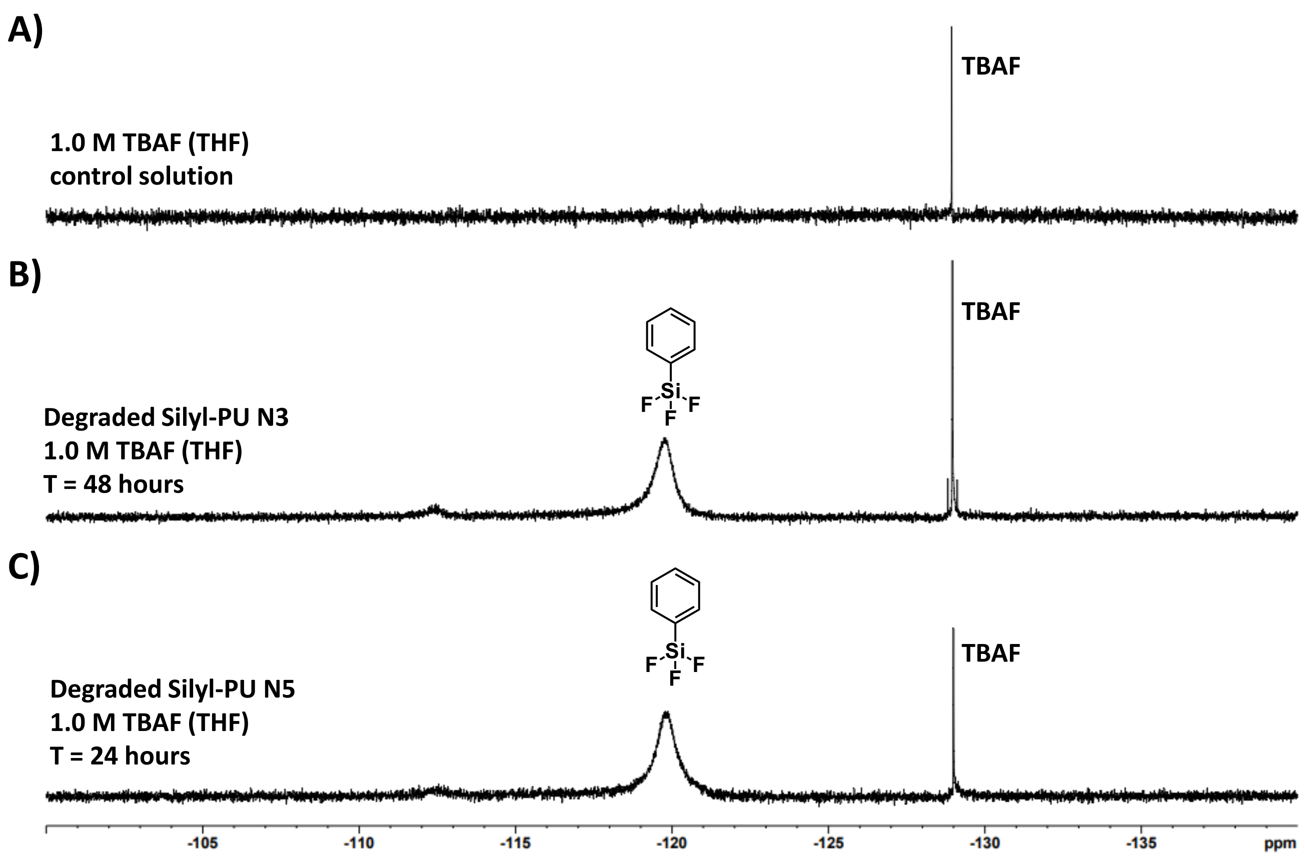


**Figure S18.** $^{19}$F NMR spectra of fluorine-containing molecules. (A) 1.0 M TBAF (THF). (B) Degraded Silyl-PU **N3** after 48 hours in 1.0 M TBAF (THF) showing trifluorophenysilane as the only product. (C) Degraded Silyl-PU **N5** after 24 hours in 1.0 M TBAF (THF) showing trifluorophenysilane as the only product.

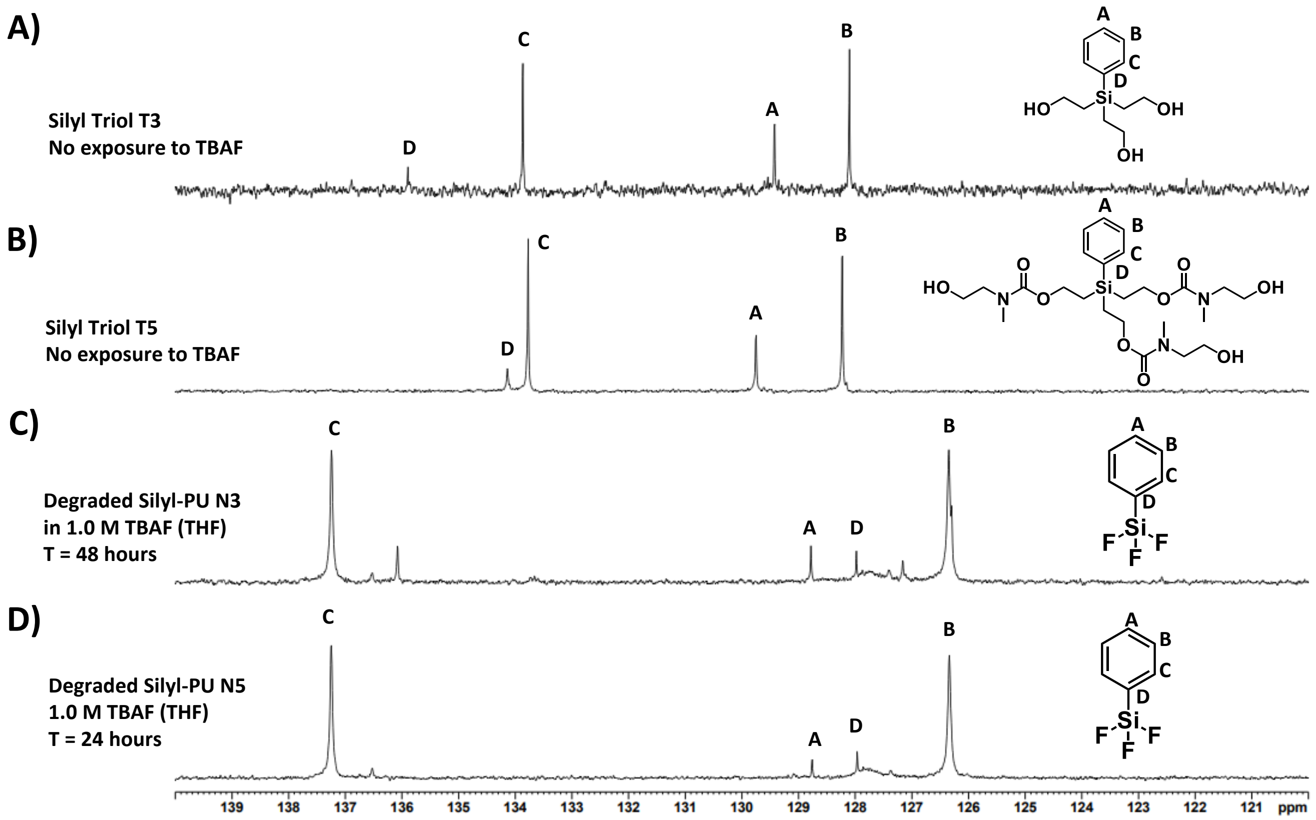


**Figure S19**. $^{13}C$ NMR spectra of aromatic region for silicon-containing molecules. (A) Silyl triol **T3**. (B) Silyl triol **T5**. (C) Silyl-PU **N3** after 48 hours in 1.0 M TBAF (THF) trifluorophenylsilane as only product. (D) Silyl-PU N5 after 24 hours in 1.0 M TBAF (THF) showing trifluorophenylsilane as only product.

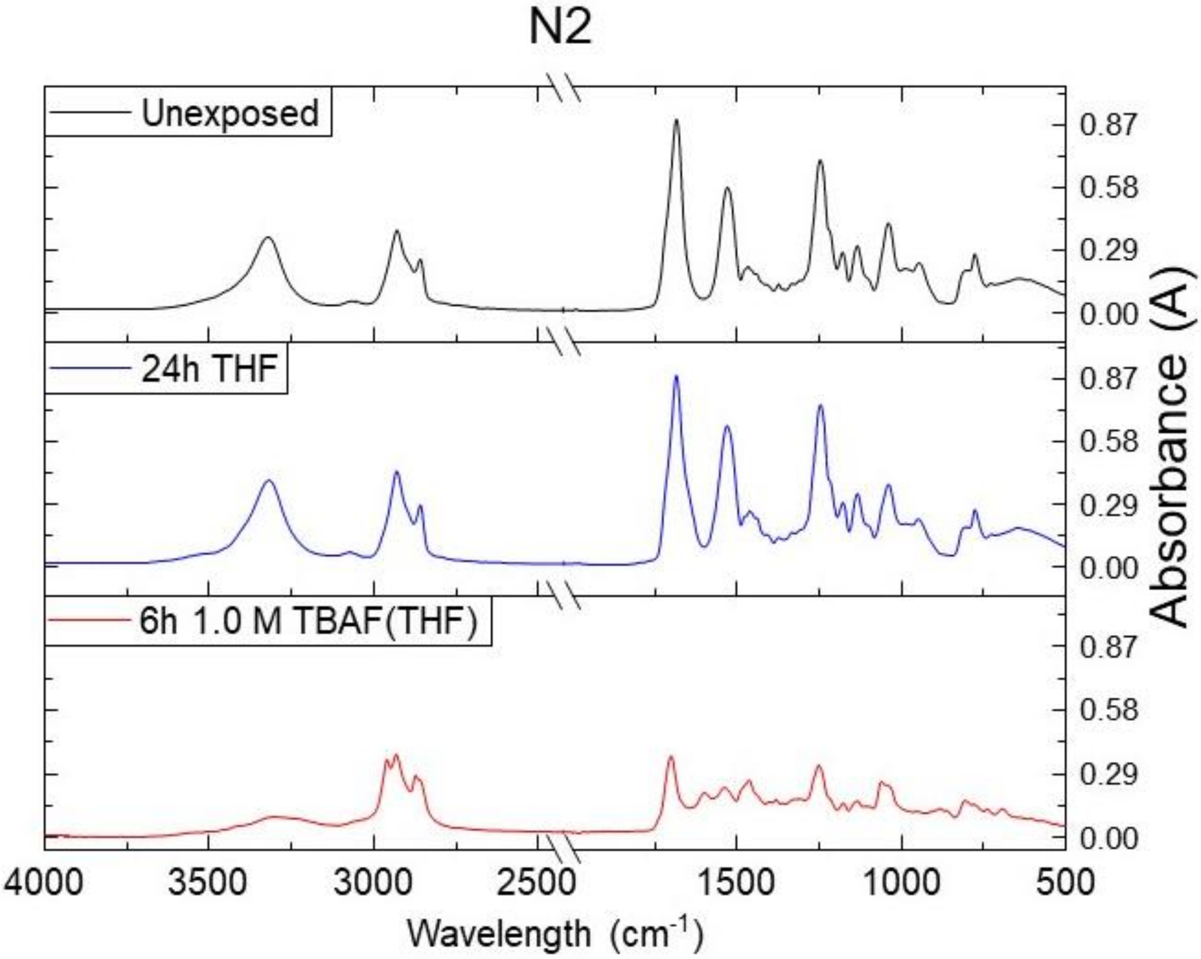


**Figure S20.** ATR-IR spectra of Silyl-PU network **N2**. (Top) Unexposed. (Middle) Exposed in THF for 24 hours. (Bottom) Exposed in 1.0 M TBAF (THF) for 6 hours.

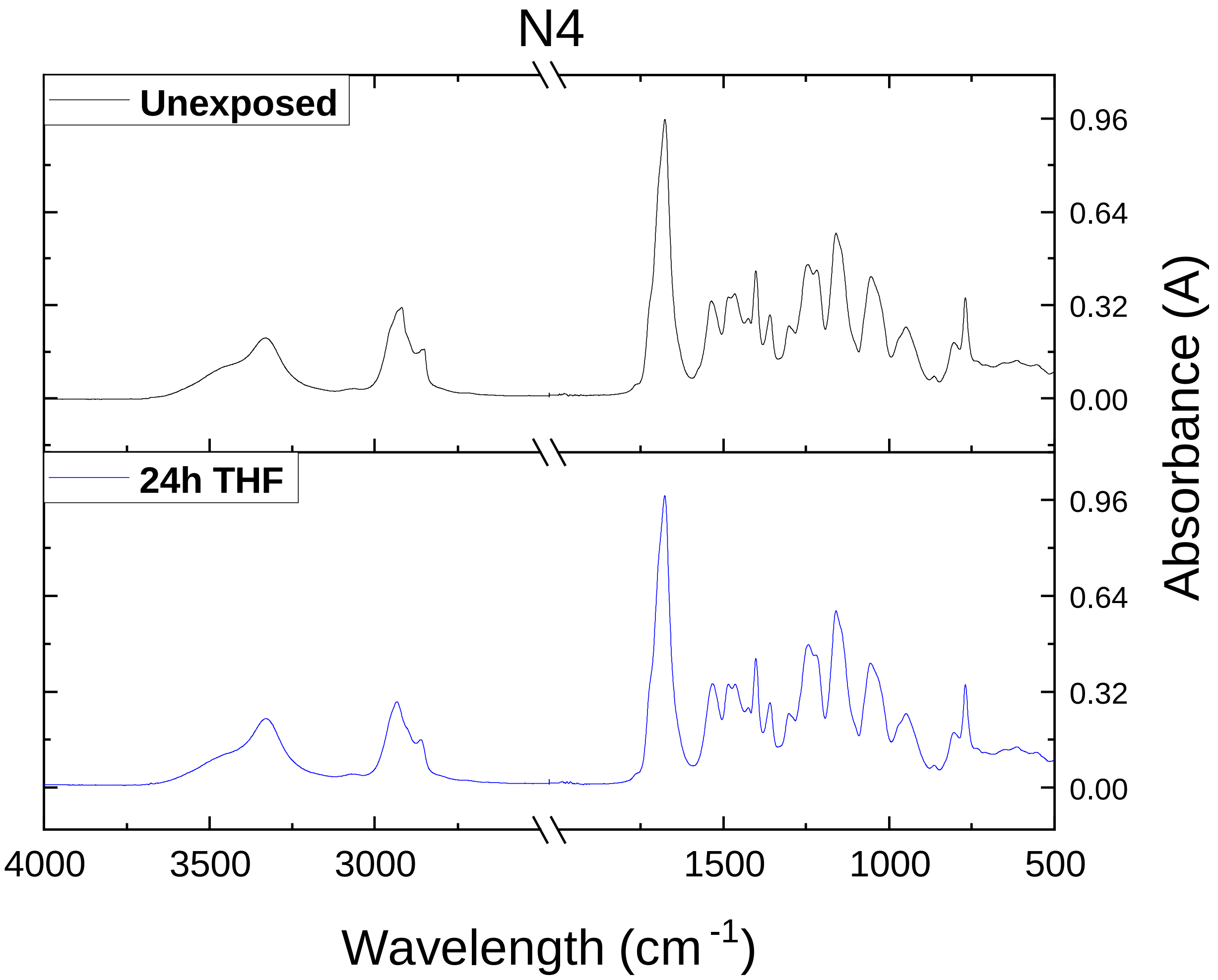


**Figure S21.** ATR-IR spectra of Silyl-PU network **N4**. (Top) Unexposed. (Bottom) Exposed in THF for 24 hours. A spectrum after 6 hours of exposure in 1.0 M TBAF (THF) was not recorded because the network was fully degraded.

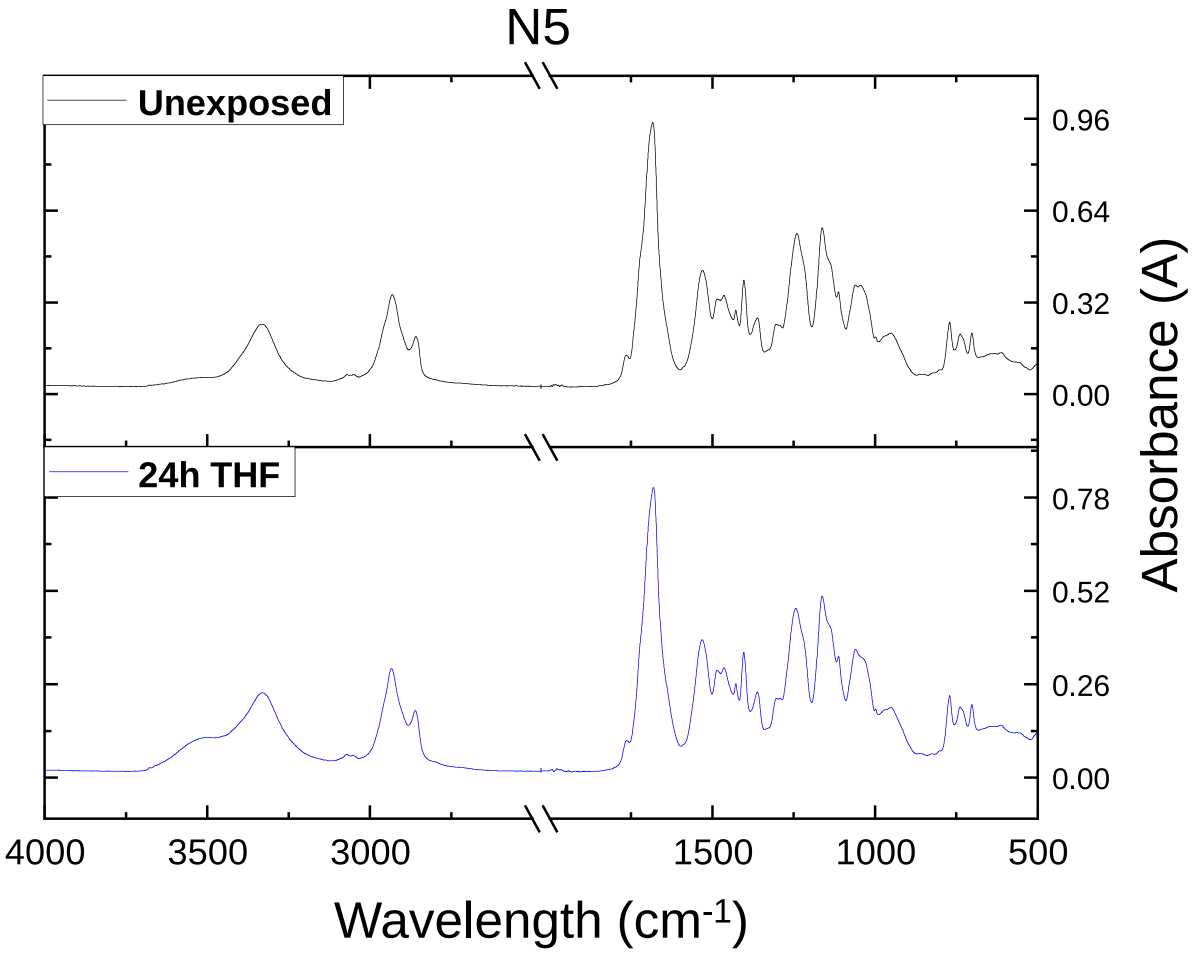


**Figure S22.** ATR-IR spectra of Silyl-PU network **N5**. (Top) Unexposed. (Bottom) Exposed in THF for 24 hours. A spectrum after 6 hours of exposure in 1.0 M TBAF (THF) was not recorded because the network was fully degraded.

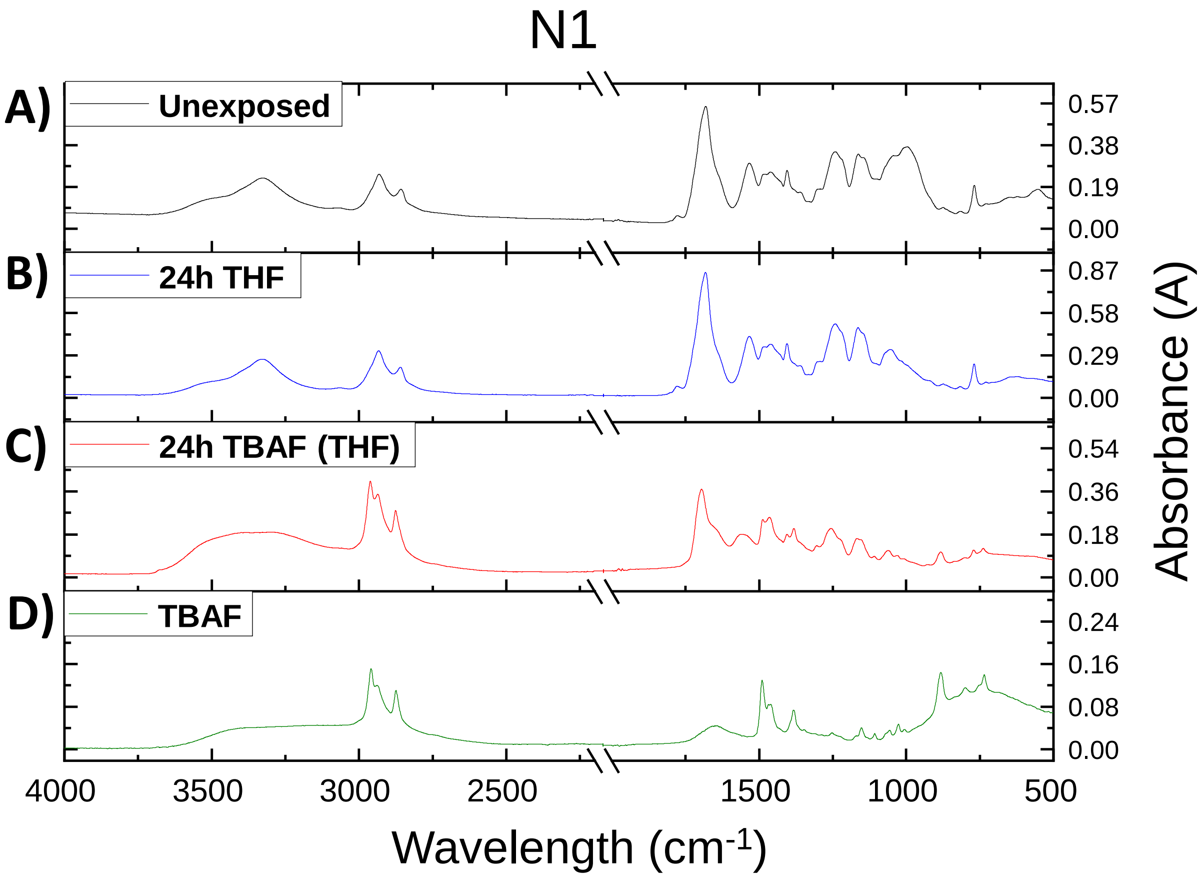


**Figure S23.** ATR-IR spectra of non-silyl containing PU control network (**N1**). (A) Unexposed. (B) Exposed in THF for 24 hours. (C) Exposed in 1.0 M TBAF (THF) for 24 hours. (D) ATR-IR spectrum of neat TBAF.

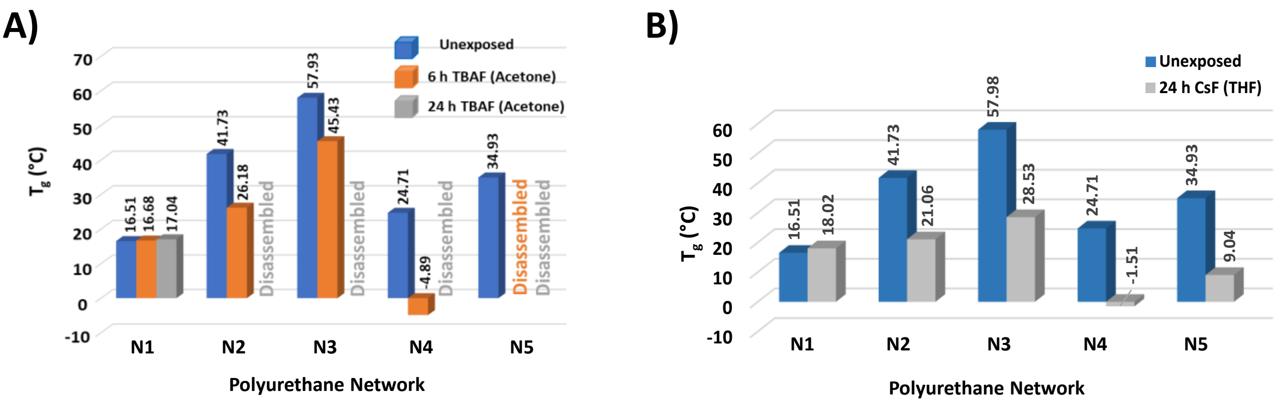


**Figure S24.** Comparison of glass transition temperatures for PU networks **N1-N5** before and after static exposures. (A) Unexposed samples (blue bars). Samples after 6 hours of exposure in 1.0 M TBAF (acetone) (orange bars). Samples after 24 hours of exposure in 1.0 M TBAF (acetone) (gray bars). (B) Unexposed samples (blue bars). Samples after 24 hours of exposure in 0.5 M CsF (THF) (gray bars).

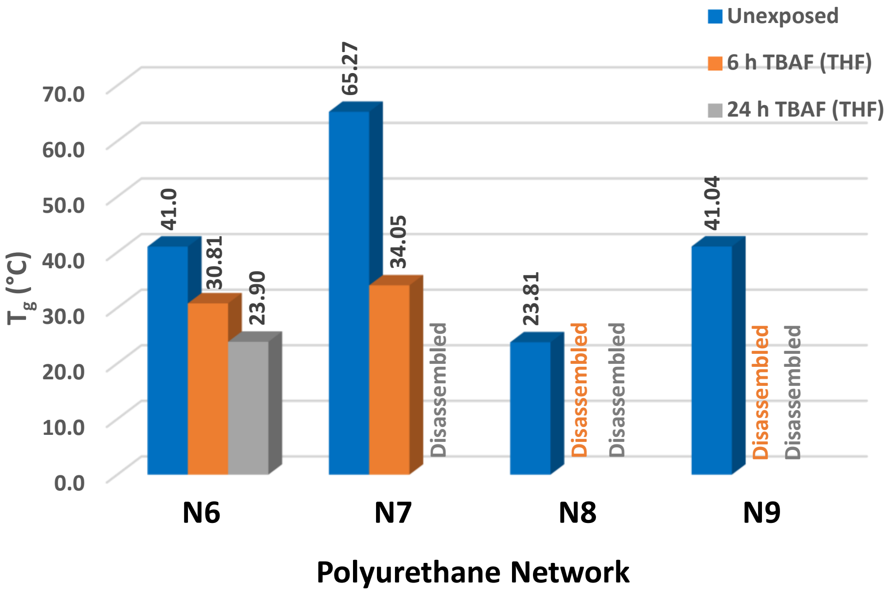


**Figure S25.** Comparison of glass transition temperatures for PU networks **N6-N9** before and after static exposure in 1.0 M TBAF (THF). Unexposed samples (blue bars). Samples after 6 hours of exposure in 1.0 M TBAF (THF) (orange bars). Samples after 24 hours of exposure in 1.0 M TBAF (THF) (gray bars).

**Table S4**. Glass transition temperature ($T_g$), as determined by DSC, for Silyl-PU thermosets **N3** and **N5** before and after 24 hours static immersion in 0.1 M TBAOH (isopropanol/methanol (10:1 v/v)) at room temperature, including **N5** after 24 hours in 1.0 M NaOH (aqueous) and 1.0 M HCl (aqueous) at room temperature.

| Silyl-PU Network | $T_g$ (°C) Prior to Exposure | $T_g$ (°C) After Exposure in 0.1 M TBAOH (IPA/MeOH) | $T_g$ (°C) After Exposure in 1.0 M NaOH (aq.) | $T_g$ (°C) After Exposure in 1.0 M HCl (aq.) |
|---|---|---|---|---|
| N3 | 57.98 ± 4.60 | 55.72 ± 5.86 | N/A | N/A |
| N5 | 34.93 ± 1.29 | 36.51 ± 6.38 | 37.16 ± 1.11 | 35.41 ± 1.29 |

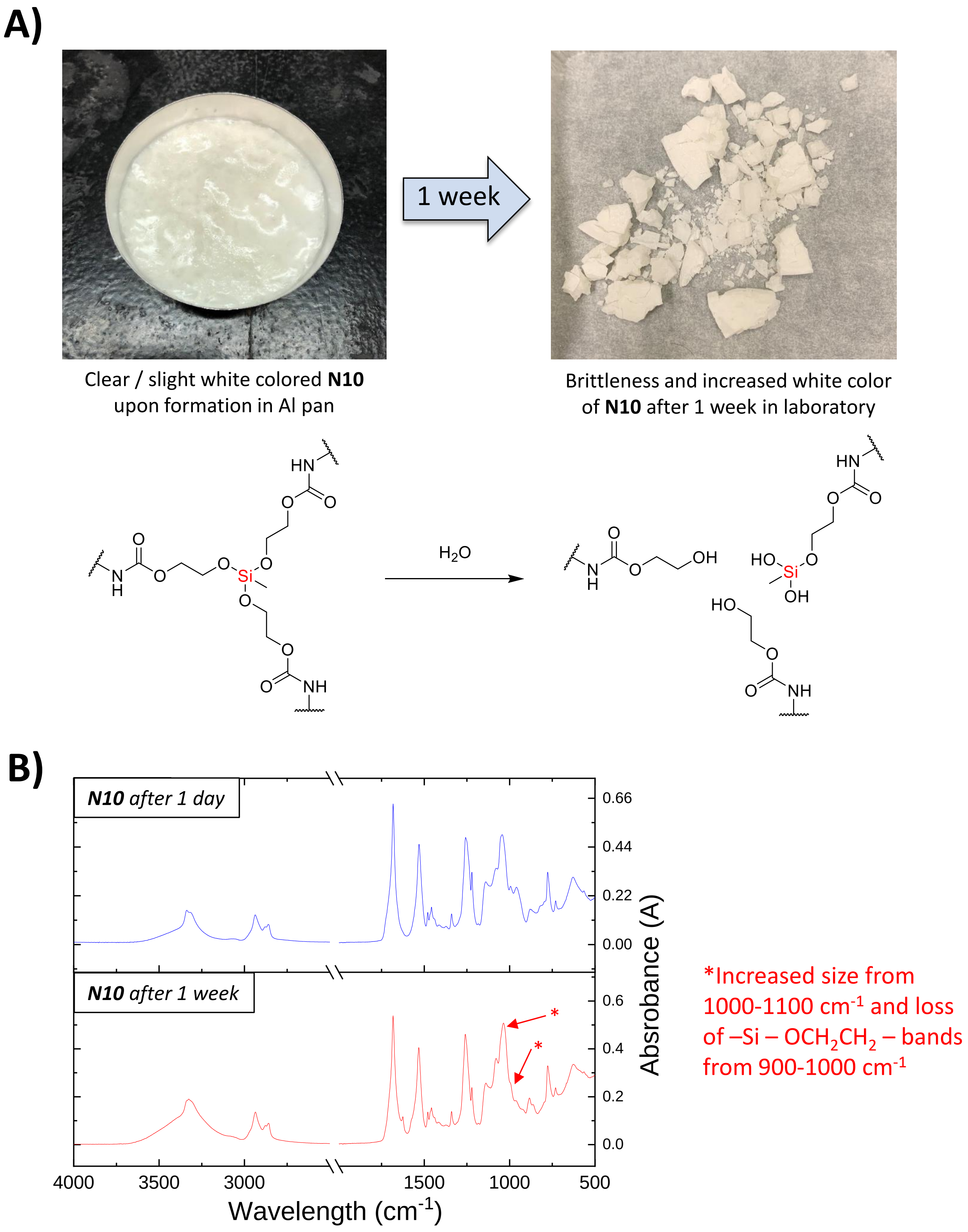


**Figure S26.** Synthesis and degradation of silyl ether containing PU network **N10**. (A) (left) Image and structure of network the day after synthesis, and (right) image and structure of network after 1 week exposure to laboratory conditions. (B) (top) ATR-IR spectra after network formation, and (bottom) ATR-IR spectra 1 week after formation showing signals for hydrolytic degradation.

**Table S5**. Glass transition temperature ($T_g$) of PU thermoset networks before and after 5 days of hygrothermal exposure as determined by DSC.

| Thermoset Network | $T_g$ (°C) prior to exposure | $T_g$ (°C) after exposure |
|---|---|---|
| PU **N1** (control) | 16.51 ± 0.93 | 16.08 ± 2.08 |
| Silyl-PU **N3** | 57.98 ± 4.60 | 55.72 ± 2.09 |
| Silyl-PU **N5** | 34.93 ± 1.29 | 34.29 ± 4.77 |

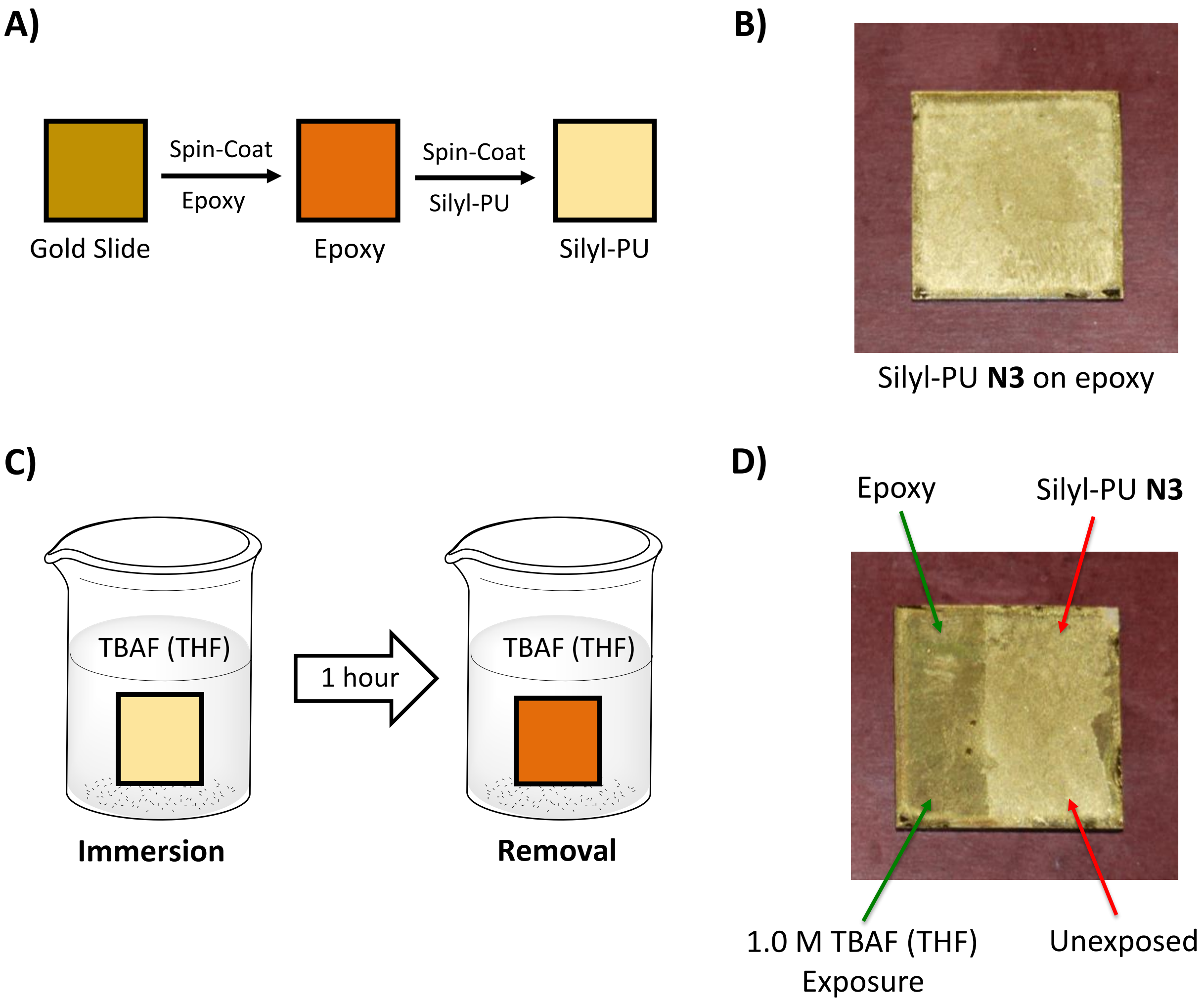


**Figure S27.** PU and epoxy networks on gold slide. (A) Spin-coating of epoxy and Silyl-PU network on gold slide. (B) Visual of sample after spin-coating Silyl-PU **N3** on the epoxy-coated gold slide. (C) Illustration of sample immersion and removal of Silyl-PU network from epoxy. (D, Left) Visual of epoxy network after exposure in 1.0 M TBAF (THF) for 1 hour. (D, Right) Visual of unexposed Silyl-PU **N3** over epoxy on same sample.

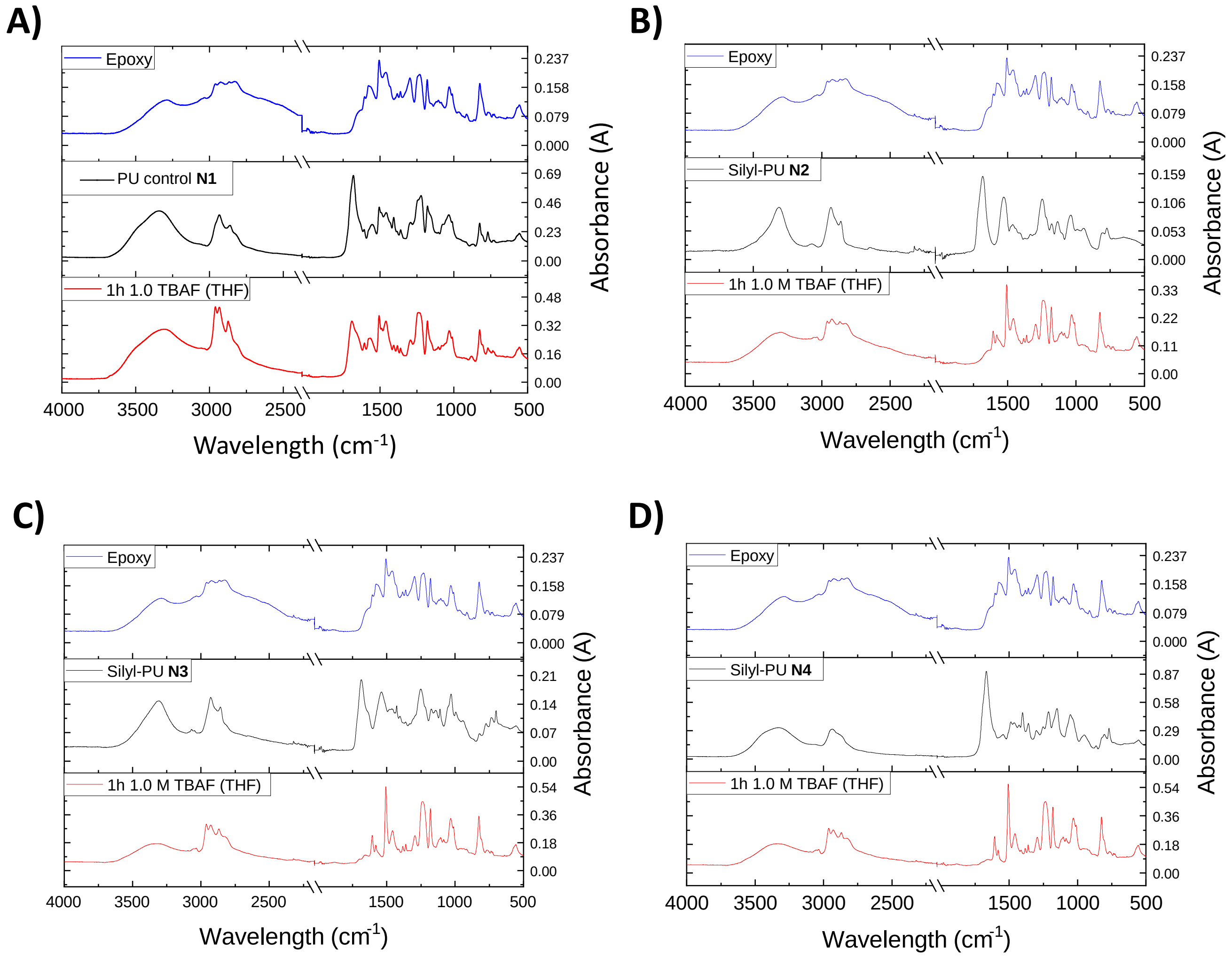


**Figure S28.** ATR-IR spectra of Silyl-PU networks **N1-N4** over epoxy network before and after immersion. (A) (Top) Epoxy over gold slide. (Middle) Non-silyl PU control (**N1**) over epoxy on gold slide. (Bottom) Gold slide after 1 hour static immersion in 1.0 M TBAF (THF) showing **N1** was not removed. (B) (Top) Epoxy over gold slide. (Middle) Silyl-PU **N2** over epoxy on gold slide. (Bottom) Gold slide after 1 hour static immersion in 1.0 M TBAF (THF) showing all of **N2** removed and epoxy chemically unchanged. (C) (Top) Epoxy over gold slide. (Middle) Silyl-PU **N3** over epoxy on gold slide. (Bottom) Gold slide after 1 hour static immersion in 1.0 M TBAF (THF) showing all of **N3** removed and epoxy chemically unchanged. (D) (Top) Epoxy over gold slide. (Middle) Silyl-PU **N4** over epoxy on gold slide. (Bottom) Gold slide after 1 hour static immersion in 1.0 M TBAF (THF) showing all of **N4** removed and epoxy chemically unchanged.

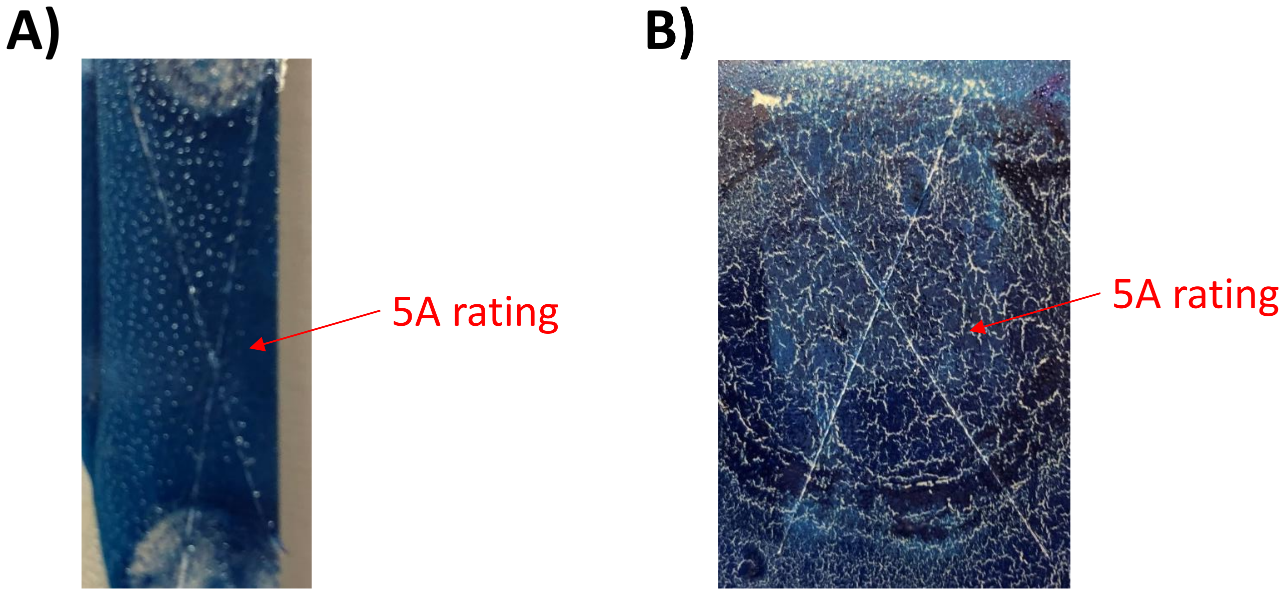


**Figure S29.** Tape adhesion testing of blue-dyed PU networks over epoxy network. (A) 5A tape adhesion rating (no peeling or removal) for Silyl-PU **N5**. (B) 5A tape adhesion rating (no peeling or removal) for non-silyl containing poly(urethane) control (**N1**).

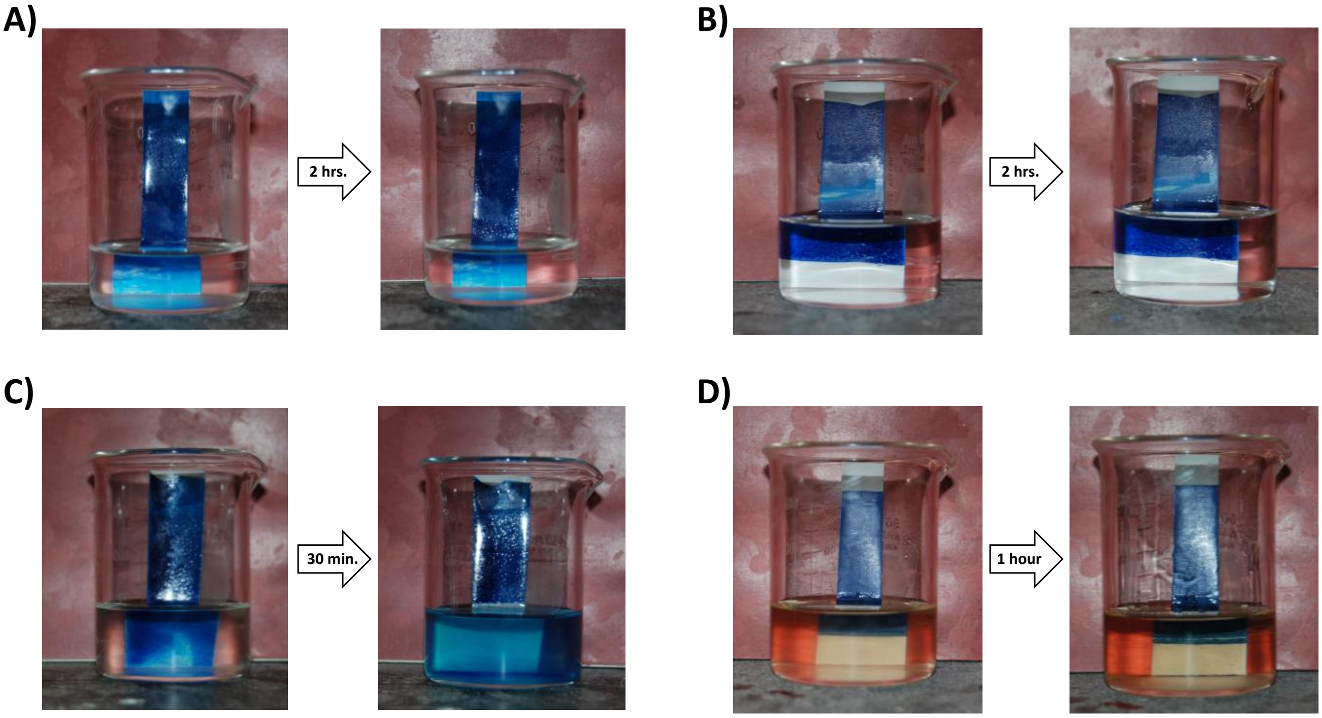


**Figure S30.** Immersion of PU networks over epoxy in static THF and 1.0 M TBAF (THF). (A) Immersion of Silyl-PU **N5** for 2 hours in THF, showing the solution did not change color because degradation did not occur. (B) Immersion of non-silyl PU control (**N1**) for 2 hours in THF, showing the solution did not change color because degradation did not occur. (C) Immersion of Silyl-PU **N5** for 30 min. in 1.0 M TBAF (THF) and resulting color change of the surrounding solution due to degradation. (D) Immersion of non-silyl PU control (**N1**) for 1 hour in 1.0 M TBAF (THF), showing the solution did not change color because degradation did not occur.

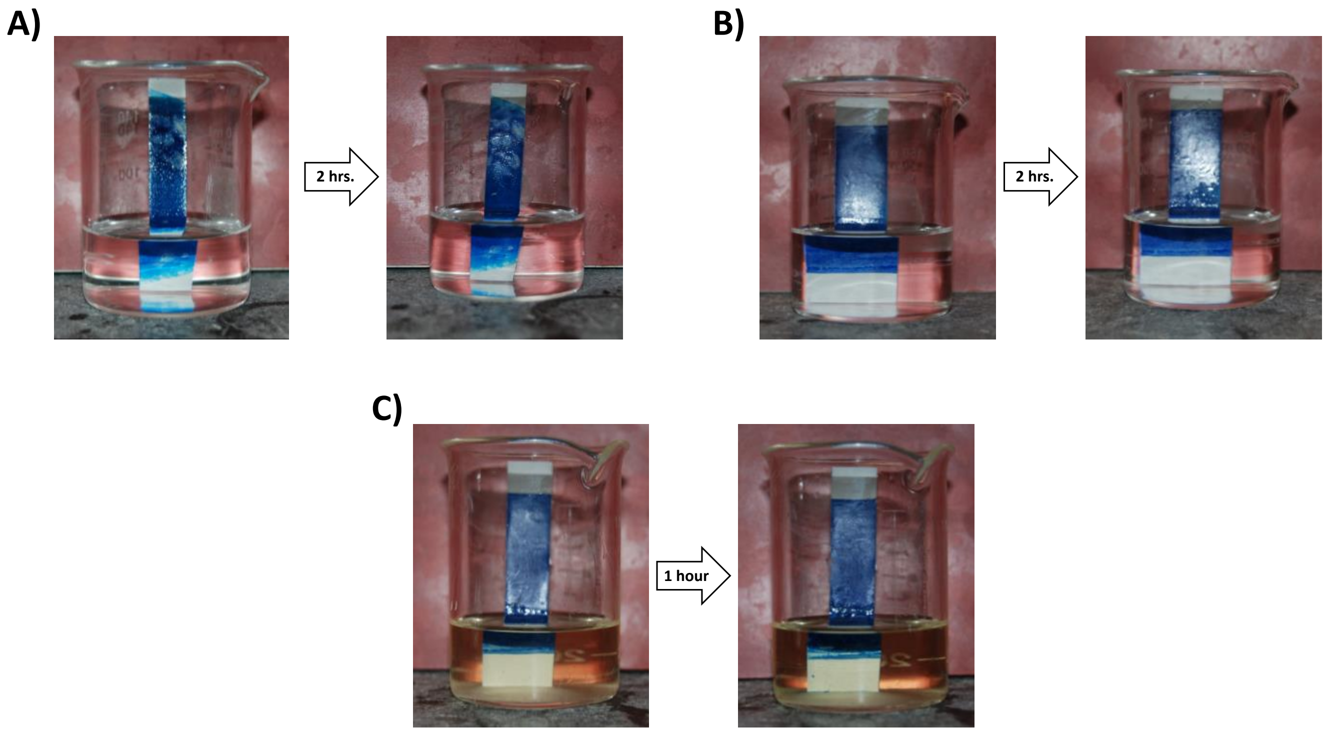


**Figure S31.** Immersion of PU networks over epoxy in static acetone and 1.0 M TBAF (acetone). (A) Immersion of Silyl-PU **N5** for 2 hours in acetone, showing the solution did not change color because degradation did not occur. (B) Immersion of non-silyl PU control (**N1**) for 2 hours in acetone, showing the solution did not change color because degradation did not occur. (C) Immersion of non-silyl PU control (**N1**) for 1 hour in 1.0 M TBAF (acetone), showing the solution did not change color because degradation did not occur.

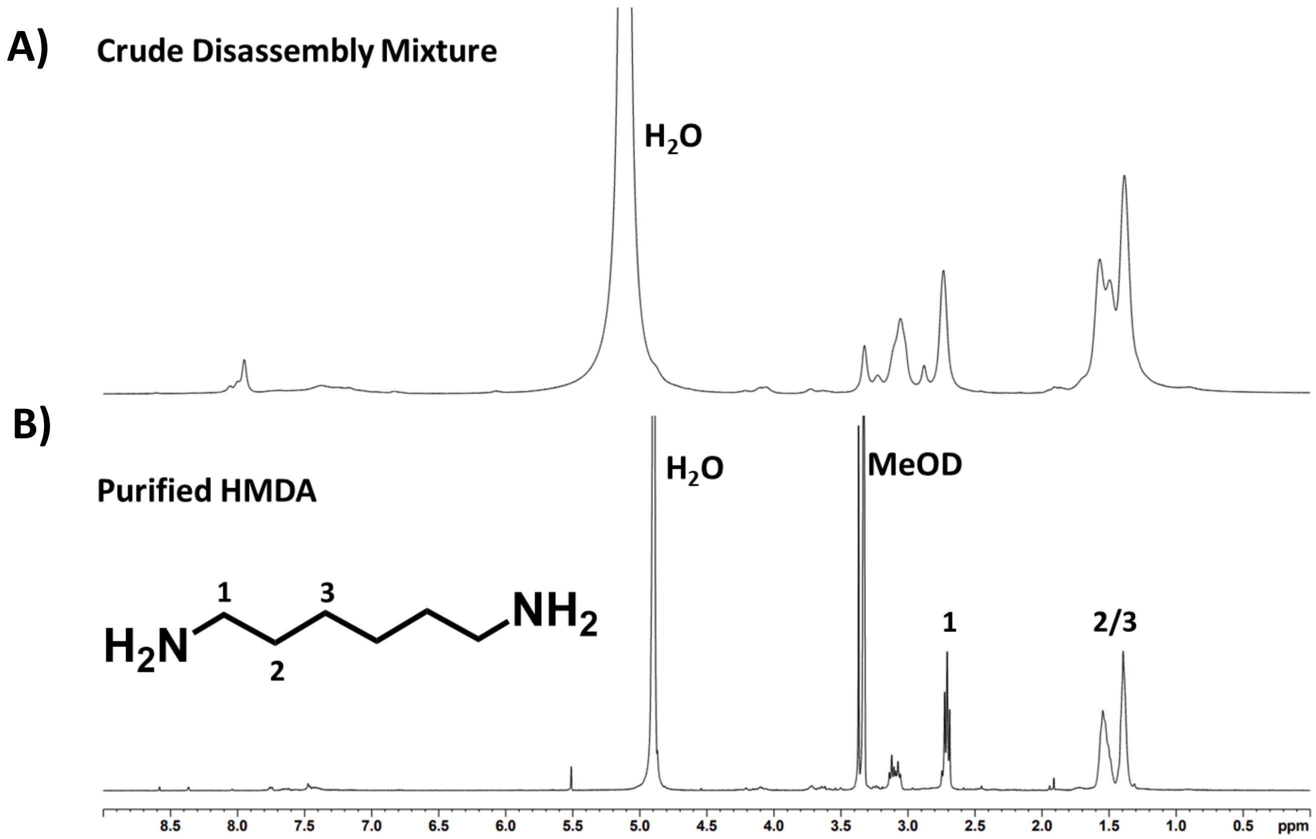


**Figure S32.** $^{1}$H NMR spectra of crude reaction mixture and recovered HMDA. (A) $^{1}$H NMR of the crude reaction mixture from degraded Silyl-PU **N3**. (B) $^{1}$H NMR of purified HMDA via aqueous extraction from crude reaction mixture of degraded **N3**. Analysis was performed in $CD_3OD$.

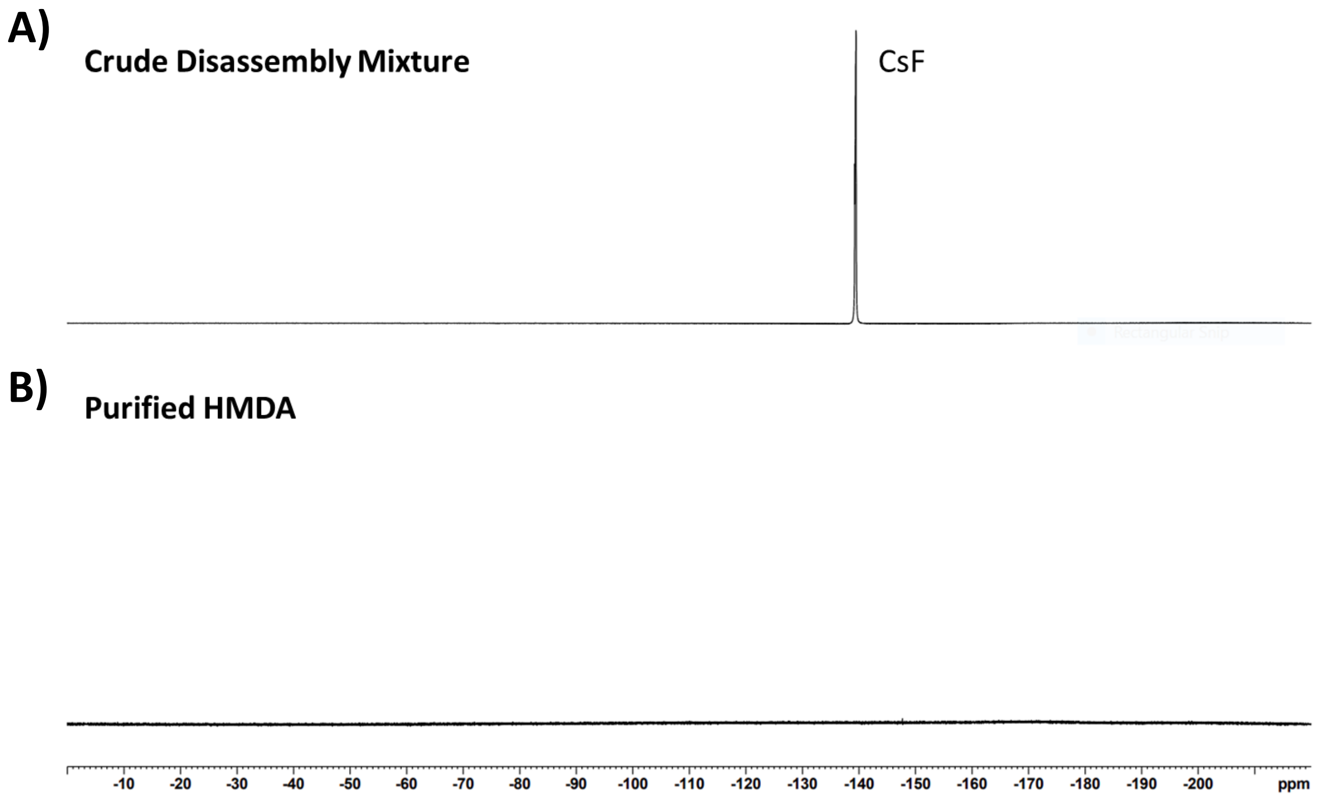


**Figure S33.** $^{19}F$ NMR spectra of crude reaction mixture and recovered HMDA. (A) $^{19}F$ NMR of the crude reaction mixture from degraded Silyl-PU **N3**. (B) $^{19}F$ NMR of purified HMDA via aqueous extraction (from crude reaction mixture) showing all fluoride ion removed. Analysis was performed in $CD_3OD$.

## 9. References

(1) Ghosh, A. K.; Duong, T. T.; McKee, S. P.; Thompson, W. J. N,N'-Disuccinimidyl Carbonate: A Ueseful Reagent for Alkoxycarbonylation of Amines, *Tetrahedron Lett.* **1992**, *33*, 2781-2784.

(2) Schaubach, S.; Gebauer, K.; Ungeheuer, F.; Hoffmeister, L.; Ilg, M. K.; Wirtz, C.; Fürstner, A. A Two-Component Alkyne Metathesis Catalyst System with an Improved Substrate Scope and Functional Group Tolerance: Development and Applications to Natural Product Synthesis. *Chem. Eur. J.* **2016**, *22* (25), 8494–8507.

(3) Coneski, P. N.; Weise, N. K.; Wynne, J. H. Synthesis and Characterization of Poly(Silyl Urethane)s Derived from Glycol-Modified Silanes. *Appl. Polym. Sci.* **2013**, *129* (1), 161–173.

(4) Gite, V. V., Mahulikar, P. P.; Hundiwale, D. G. Preparation and Properties of Polyurethane Coatings Based on Acrylic Polyols and Trimer of Isophorone Diisocyanate. *Prog. Org. Coat.* **2010**, *68*, 307–312.

(5) Trzebiatowska, P. J.; Echart, A. S.; Correas, T. C.; Eceiza, A.; Datta, J. The Changes of Crosslink Density of Polyurethanes Synthesised with using Recycled Component. Chemical Structure and Mechanical Properties Investigations. *Prog. Org. Coat.* **2018**, *115*, 41–48.

(6) Fridrihsone-Girone, A.; Stirna, U.; Marija Misāne, M.; Lazdiņa¸ B.; Deme L. Spray-Applied 100% Volatile Organic Compounds Free Two Component Polyurethane Coatings Based on Rapeseed Oil Polyols. *Prog. Org. Coat.* **2016**, *94*, 90–97.

(7) Desai, S.; Thakore, I. M.; Sarawade, B. D.; Devi, S. Structure-Property Relationship in Polyurethane Elastomers Containing Starch as a Crosslinker. *Polym. Eng. Sci.* **2000**, *40* (5), 1200–1210.

(8) Bao, C.; Zhang, X.; Yu, P.; Li, Q.; Qin, Y.; Xin, Z. Facile Fabrication of Degradable Polyurethane Thermosets with High-Mechanical Strength and Toughness via the Cross-Linking of Triple Boron-Urethane Bonds. *J. Mater. Chem. A,* **2021**, *9*, 22410–22417.

(9) Desai, S.; Thakore, I. M.; Sarawade, B. D.; Devi, S. Effect of Polyols and Diisocyanates on Thermo-Mechanical and Morphological Properties of Polyurethanes. *Eur. Polym. J.* **2000**, *36*, 711–725.

(10) Kumar, R.; Narayan, R.; Aminabhavi, T. M.; Raju, K. V. S. N. Nitrogen Rich Hyperbranched Polyol via $A_3 + B_3$ Polycondensation: Thermal, Mechanical, Anti-Corrosive and Antimicrobial Properties of Poly(urethane-urea). *J. Polym. Res.* **2014**, *21***,** 547.
